# Structure-Function Mutational Analysis and Prediction of the Potential Impact of High Risk Non-Synonymous Single-Nucleotide Polymorphism on Poliovirus 2A Protease Stability Using Comprehensive Informatics Approaches

**DOI:** 10.3390/genes9050228

**Published:** 2018-04-26

**Authors:** Amna Younus, Saba Munawar, Muhammad Faraz Bhatti, Aqsa Ikram, Faryal Mehwish Awan, Ishrat Jabeen, Nasar Virk, Hussnain Ahmed Janjua, Muhammad Arshad

**Affiliations:** 1 Atta-ur-Rahman School of Applied Biosciences (ASAB), National University of Sciences and Technology (NUST), Sector H-12, Kashmir Highway, Islamabad 44000, Pakistan; amna.11.dirphd.07@asab.nust.edu.pk (A.Y.); aqsa_ikram@yahoo.com (A.I.); faryal_mehwish@yahoo.com (F.M.A.); nasarvirk@asab.nust.edu.pk (N.V.); hussnain.janjua@asab.nust.edu.pk (H.A.J.); 2 Research Center for Modeling and Simulation (RCMS), National University of Sciences and Technology (NUST), Sector H-12, Kashmir Highway, Islamabad 44000, Pakistan; sabamunawar@rcms.nust.edu.pk (S.M.); ishrat.jabeen@rcms.nust.edu.pk (I.J.); 3 Department of Bioinformatics and Biotechnology, International Islamic University, Sector H-10, Islamabad 44000, Pakistan; m.arshad@iiu.edu.pk

**Keywords:** poliovirus 2A protease (PV2A^pr^°), docking, single-nucleotide polymorphisms (SNPs), non-synonymous SNPs (nsSNPs), computational analysis, drug binding sites, stability changes

## Abstract

Polio viral proteinase 2A performs several essential functions in genome replication. Its inhibition prevents viral replication, thus making it an excellent substrate for drug development. In this study, the three-dimensional structure of 2A protease was determined and optimized by homology modelling. To predict the molecular basis of the interaction of small molecular agonists, docking simulations were performed on a structurally diverse dataset of poliovirus 2A protease (PV2A^pr^°) inhibitors. Docking results were employed to identify high risk missense mutations that are highly damaging to the structure, as well as the function, of the protease. Intrinsic disorder regions (IDRs), drug binding sites (DBS), and protein stability changes upon mutations were also identified among them. Our results demonstrated dominant roles for Lys 15, His 20, Cys 55, Cys 57, Cys 64, Asp 108, Cys 109 and Gly 110, indicating the presence of various important drug binding sites of the protein. Upon subjecting these sites to single-nucleotide polymorphism (SNP) analysis, we observed that out of 155 high risk SNPs, 139 residues decrease the protein stability. We conclude that these missense mutations can affect the functionality of the 2A protease, and that identified protein binding sites can be directed for the attachment and inhibition of the target proteins.

## 1. Introduction

Pakistan is making significant progress in interrupting and eradicating poliovirus (PV) from the country, which is still currently categorized as endemic for poliovirus transmission. No drugs are yet available to treat infected polio sufferers. Polio antiviral drug development will depend on ongoing strong support for poliovirus research. Different programs have been initiated by World Health Organization (WHO) and Centers for Disease Control and Prevention (CDC) to combat this disease. Global Polio Eradication Initiative (GPEI) was started in 1988 and has successfully eradicated polio in many regions of the world by using vaccines at a large scale level [1]. Poliovirus Antivirals Initiative (PAI) have made the considerable progress in the discovery and development of poliovirus antiviral agents (pocapavir and V-7404) [2].

Poliovirus is a human enterovirus which possesses a ~7.5 kb of positive strand RNA genome and a non-enveloped icosahedral protein capsid [3]. Replication of PV is achieved using a single open reading frame, called a polyprotein, which further splits into three main fragments of structural and non-structural proteins (P1, P2, and P3) by means of 2A and 3A viral proteases. Two cysteine proteases (2A and 3C) are involved in proteolytic processing of polyprotein, making them crucial for the viral life cycle [4]. PV2A^pr^° belongs to the cysteine protease group, comprising 149 amino acids. Combined capsid proteins are considered to be cleaved at their amino terminus by PV2A^pr^° in an autocatalytic cleavage manner. Additionally, PV2A^pr^° is involved in the cleavage of eukaryotic translation initiation factor 4G (eIF4G), thus acting as a multifunctional enzyme [5,6]. Cleavage at eIF4GI by enterovirus 2A proteases (PV, Human Rhinovirus (HRV), and coxsackieviruses) at positions 681/682 is suggested to be conserved of specificity for the substrate determinants [7]. Studies have proved that PV2A^pr^° activity is inhibited by alkylating agents, such as iodoacetamide and *N*-ethylmaleimide, and elastase-specific inhibitors, such as elastatinal and methoxysuccinyl-Ala-Ala-Pro-Val-chloromethylketone (MCPK) [8].

Single-nucleotide polymorphisms (SNPs) are important in understanding the genetics of many complex diseases, and identifying the functional SNPs in a disease-related gene is still an important limiting factor. Non-synonymous single-nucleotide polymorphisms (nsSNPs) cause changes in the amino acid residues, and are important factors contributing to the functional diversity of the encoded proteins [9]. Computer alignments of 2A^pr^° amino acid sequences have shown a common consensus sequence near the active center Cys residue of picornavirus 3C^pr^°, which is highly conserved among enterovirus and rhinovirus 2A^pr^°, as well as certain serine proteases [10,11,12]. Studies suggest that highly conserved residues, including His 20, Asp 38, and Cys 109, may contribute in the complete catalytic triad of 2A^pr^°, thus supporting the hypothesis that poliovirus 2A^pr^° is structurally related to the trypsin-like serine proteases with the substitution of cysteine 109 as the active site nucleophile. Studies showing that these highly conserved cysteine and histidine residues, including Cys 55, Cys 57, Cys l15, and His 117, essentially support the catalytic activity of 2A^pr^° [13]. Moreover, a number of residues are involved in substrate recognition outside the catalytic pocket of PV2A^pr^° [13,14]. In a recent study, poliovirus 2A protease was amplified and sequenced from blood samples of polio. Sequencing results showed some mutations in the protease, which were then employed for structural and drug binding sites analysis using various in silico tools, including Swiss modeling [15], Volume Area Dihedral Angle Reporter (VADAR) [16], COACH/I-Tasser (Interative Threading ASSEmbly Refiniment) [17], subCELlular LOcalization predictor (CELLO) V 2.5 [18], and Tm Finder [19,20]. Important ligand interactions have been identified among conserved regions of the protein, amplified from the two blood samples of poliovirus type 1 [20]. This study aims to identify drug targets of poliovirus 2Aprotease (Type I, II and III) from all sequences retrieved from the National Center for Biotechnology Information (NCBI).

Drug targets are biological targets or in vivo binding sites consisting of receptors, enzymes, ion channels, and amino acids, etc. Drugs bind to their matching targets, thus executing the desirable therapeutic effects [21]. 2A^pr^° and 3C^pr^° have been considered as potential therapeutic targets due to their unique structure and function with respect to viral replication and disease progression [22,23].

**Figure 1:**
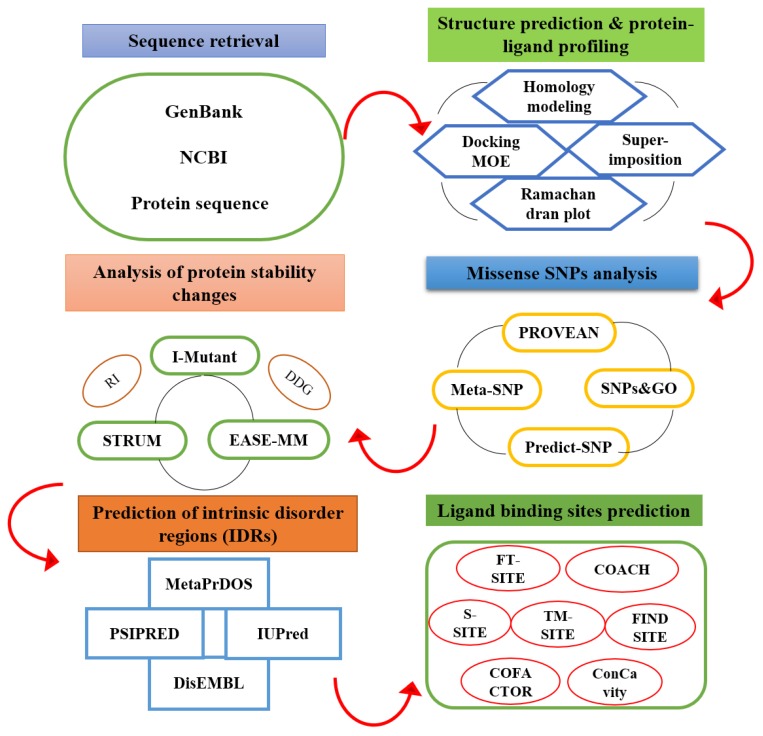
Schematic representation of designed study to find drug binding sites of poliovirus 2A protease along with missense single-nucleotide polymorphisms (SNPs). MOE: Molecular Operating Environment, RI: Reliability Index DDG: Free Energy change value.

The present study aimed to explore PV2A^pr^° as probable drug targets, and to identify the functional and structural impact of nsSNPs of PV2A^pr^°, along with mapping of active sites on PV2A^pr^° using molecular docking protocols, where antiviral agents might potentially bind (Figure 1). Computational analysis of the functional and structural effect of highly damaging SNPs will be helpful in facilitating in finding true disease associations. We propose that this study will help in understanding and predicting highly damaging missense SNPs and potential binding sites to discover the mutation structure–function relationship in PV2A^pr^°. Following such structure-function investigations, it is predicted that novel agents that inhibit PV through interference with proteolytic processing in virus replication might be identified.

## 2. Materials and Methods

### 2.1. Protein Sequence Dataset

The protein sequences of PV2A^pr^° were retrieved from the NCBI GenBank (https://www.ncbi.nlm.nih.gov/genbank/). We retrieved all PV2A^pr^° sequences reported to date, from NCBI. A total of 553 sequences were retrieved from the database. The selected sequences representing all three serotypes (Type 1–3) were selected from various geographical regions, including Africa, the United States of America, Belarus, Brazil, Cameroon, Chad, China, Congo, Egypt, Estonia, Finland, Greece, Guinea, India, Iran, Israel, Kazakhstan, Madagascar, Nigeria, Pakistan, Peru, the Philippines, Russia, Spain, Switzerland, Taiwan, Tajikistan, Turkmenistan, and the United Kingdom. Out of 553 sequences, 250, 172, and 131 were from serotype 1, 2, and 3, respectively. These sequences include all vaccine-derived and wild type genotypes of poliovirus.

### 2.2. Protein Conservation Analysis

For sequence conservation analysis, sequences were aligned to multiple sequence alignment (MSA) and position-wise diversity using the ClustalW [24] CLC sequence viewer v3.6 Workbench [25], BioEdit v. 7.2.3 [26], and Datamonkey [27]. The sequences from each serotype were first aligned to obtain a consensus sequence, and subsequently these consensus sequences were aligned with each other.

### 2.3. Prediction of Intrinsic Disorder Regions

Intrinsic disorder regions are misfolded regions of the viral proteins, with most viruses using these regions to proceed with their viral processes. In our analysis, we included four software packages to identify disordered positions in PV2A^pr^° based on the majority representation by these software tools. In this context, a residue is considered as disordered if its score is above 0.5. Based on the real valued disorder propensities generated by MetaPrDOS [28], PSIPRED [29], and IUPred [30], disordered regions are defined as regions with scores above the grey line (threshold level generated by the software).

### 2.4. Drug Binding Site Predictions

Possible drug binding sites were predicted against polio 2A^pr^°, in order to identify the possible drug target within this protein. Protein Data Bank (PDB) structure was generated by I-Tasser/Modeller [17,31] by the software COACH [17] and FTSite [32]. A meta-server approach and energy-based method for binding ligand site prediction involved the use of COACH [17] and FTSite [32], respectively. The overall results were given in the form of position of potential drug binding sites. Only consensus binding sites predicted by these two software packages were taken.

### 2.5. Structure Prediction and Ligand-Protein Interaction Profiling

The three-dimensional structure of PV2A^pr^° was predicted by homology modeling using Coxsackievirus B4 2A proteinase (pdb 1Z8R) as a template. The homology model was generated with the modeling program Modeller v.9.14 [31] using automated sequence alignment by Clustal Omega [33]. Briefly, 100 independent models were generated, and energy minimized using MMFF94x force field. The final model was selected on the basis of the smallest number of outliers in a Ramachandran plot [34].

A set of four PV2A^pr^° inhibitors, including rupintrivir, MCPK, Z-Val-Ala-Asp-(OMe)-Fluoromethyl Ketone (Z-VAD), and elastatinal were docked into the binding cavity of PV2A^pr^°. An area of 5 Å around already-known interacting amino acids His 20, Asp 38, Cys 109 was considered as a potential binding pocket.

A total of 100 poses per ligand were generated using scoring function London scoring function (dG) and Alpha triangle matcher implemented in software MOE v.2013 [35]. All ligand-protein complexes were refined, and the energy minimized using MMFF94x force field parameters [36]. Final pose selection was performed using consensus scoring using GBVI/WSA dG, London dG scoring functions [37] of already-docked poses. The top ten best scored poses by each scoring function were considered for consensus; however, only those poses that showed maximum overlap of all scoring functions were selected for final ligand-protein interaction profiling.

### 2.6. Retrieval of Missense Single-Nucleotide Polymorphisms Datasets

After docking analysis, certain amino acid residues showed potential binding activities, and all such amino acid residues were subjected to missense SNP analysis. These amino acids include Gly 1, Lys 15, His 20, Cys 55, Cys 57, Cys 64, Asp 108, Cys 109, and Gly 110.

### 2.7. Analysis of Functional Consequences of Missense Single-Nucleotide Polymorphisms

To assess the potential functional effect of the missense SNPs, 15 prediction tools were utilized, which were further distributed into four different methods. All filtered missense SNPs were classified as deleterious by at least three tools in each of the four groups, and these were denominated as convergent deleterious predicted SNPs.

A missense SNP algorithm was achieved by means of the use of four tools: PROVEAN (Protein Variation Effect Analyzer) [38], SNPs & Gene Ontology (GO) [39], Meta-SNP [40] and PredictSNP [41]. SNPs predicted as deleterious by at least three servers were considered damaging.

To predict the functional aspects of SNPs, an alignment-based scoring method was conducted through PROVEAN. In PROVEAN, a threshold of −2.5 was used (a score of −2.5 was considered “deleterious”, while a score > −2.5 was considered “neutral”).

In order to predict whether a certain mutation is disease-related or not, an SNPs&GO algorithm was used [39]; this tool accurately uses protein GO annotation information to predict the relationship of mutation with the disease.

To reduce the bias of a single predictor, Meta-SNP [40] was used since it incorporates four existing methods: PANTHER (Protein ANalysis THrough Evolutionary Relationships) [42], PhD-SNP (Predictor of Human Deleterious Single Nucleotide Polymorphisms) [43], SIFT (Sorting Intolerant From Tolerant) [44], and SNAP (screening for non-acceptable polymorphisms) [45] for the detection of disease-associated nsSNVs. PANTHER, SIFT, and SNAP help in variant annotation in terms of their being disruptive to protein function or equivalent to wild-type, whereas PhD-SNP mainly identifies disease-associated substitutions [43].

To enhance the performance further, PredictSNP was used. This tool characterizes precise and robust alternatives to the predictions delivered by individual tools [41]. For the prediction of the effects of mutations on 2A^pr^° function, PredictSNP operates by allowing easy access to all eight unbiased prediction tools: nsSNPAnalyzer [46], PolyPhen-1(Polymorphism Phenotyping; PPH-1) [47], PolyPhen-2 (PPH-2) [48], SNAP [47], MAPP (Multivariate Analysis of Protein Polymorphism) [49], PhD-SNP [43], SIFT [44], and consensus PredictSNP, thus resulting in significantly improved prediction performance.

### 2.8. Analysis of Protein Stability Changes Due to High Risk Missense Single-Nucleotide Polymorphisms

To quantitatively predict the change in protein stability due to high risk missense mutations, I-Mutant v. 2.0 [50], STRUM [51], and EASE-MM (sequence-based prediction of mutation-induced stability changes with feature-based multiple models) [52] web servers were used.

## 3. Results

### 3.1. Protein Conservation Analysis

PV2A^pr^° sequences were selected from diverse geographical regions, including Africa, the United States of America, Belarus, Brazil, Cameroon, Chad, China, Congo, Egypt, Estonia, Finland, Greece, Guinea, India, Iran, Israel, Kazakhstan, Madagascar, Nigeria, Pakistan, Peru, the Philippines, Russia, Spain, Switzerland, Taiwan, Tajikistan, Turkmenistan, and the United Kingdom. These sequences represent all serotypes of poliovirus, and show many conserved patterns among all the serotypes (File S1).

### 3.2. Intrinsic Disorder Regions of Polio 2A^pr^°

**Figure 2:**
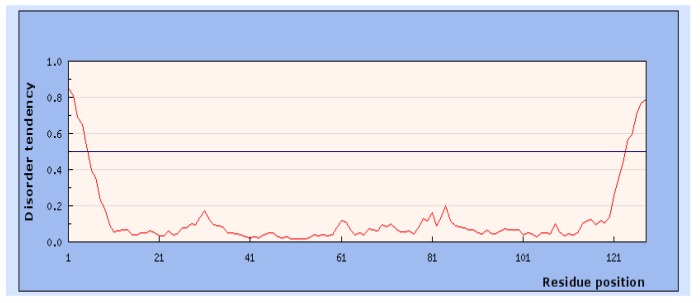
Intrinsic disordered regions (IDRs) of PV2A^pr^°. Grey line indicating threshold line. The regions above the line represents the disordered regions. X-axis: Residue position, Y-axis: Disorder tendency score

For identifying intrinsic disorder regions in polio 2A^pr^°, three software packages were employed. Results were compared with all three software packages. This was performed to ensure that drug binding sites did not lie within this region. In our analysis, it was observed that polio 2A^pr^° possesses only a few intrinsic disorder regions, proposing it as a potential drug target protein (Figure 2). Amino acid residues GFGH (1–4) and EAMEQ (145–149) were found in Intrinsic Disordered regions (IDR).

### 3.3. Drug Binding Site Predictions of Polio 2A^pr^°

**Figure 3:**
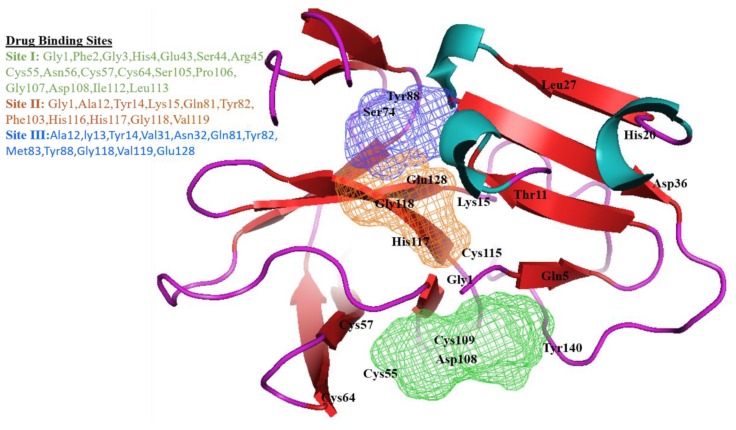
Representation of poliovirus 2A^pr^° model along with binding site predictions. Ligand binding pockets were annotated by FTSite [32]. First binding site is in green color, while second and third are in orange and blue, respectively.

To predict the drug binding sites of polio 2Apro, COACH and FTSite algorithms were employed using PDB file as an input. The consensus drug targets predicted by these two algorithms were taken for further evaluation. In this protein, three potential binding sites were predicted using FTSite. Site one possesses 17 binding residues, including Gly 1, Phe 2, Gly 3, His 4, Glu 43, Ser 44, Arg 45, Cys 55, Asn 56, Cys 57, Cys 64, Ser 105, Pro 106, Gly 107, Asp 108, Ile 112, and Leu 113. The second binding site includes the eleven residues, Gly 1, Ala 12, Tyr 14, Lys 15, Gln 81, Tyr 82, Phe 103, His 116, His 117, Gly 118, and Val 119, while site 3 was predicted to have eleven binding residues (Ala 12, Gly 13, Val 31, Asn 32, Gln 81, Tyr 82, Met 83, Tyr 88, Val 119, and Glu 128; Figure 3). These results showed the drug potential of this viral protein. Further analysis revealed that five high risk missense SNPs were located in these binding sites (Lys 15, Cys 55, Cys 57, Cys 64, and Asp 108).

### 3.4. Structure Prediction and Ligand-Protein Interaction Profiling

**Figure 4:**
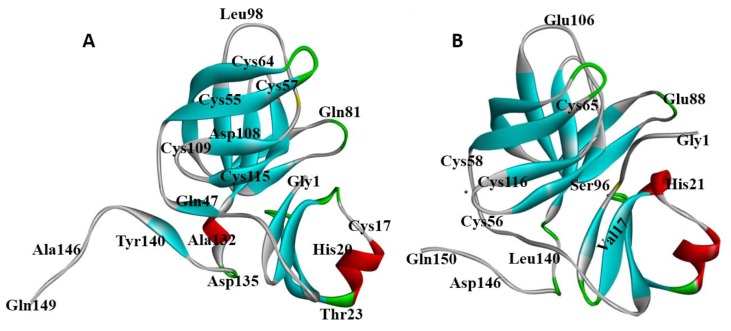
3D and crystalline structures of respectively; (**A**) poliovirus 2A^pr^° and (**B**) Coxsakievirus B4 1z8r as predicted by the Modeller v. 9.14 program [31].

An understanding of the molecular basis of ligand-protein interactions in virus-encoded proteins could pave the way towards development of potential therapeutic agents against PV infection. In the absence of any crystallographic data for PV2A, we used homology modeling to compare it with an equivalent protease from coxsackievirus B4, which has been crystallized and is 56% identical in sequence to PV2A^pr^°. Coxsackievirus B4 belongs to the family *Picornaviridae*, and is a target for natural killer cells and pancreatic islet cells, resulting in cell apoptosis [53]. Following the homology modeling protocol, described similarities between a folding structure and active site catalytic residues were discovered in both template and target proteins (Figure 4). Briefly, following the generation of 100 independent models using Modeller v.9.14, Ramachandran plots of the final model revealed that 90.5% of the residues lie in the most favored position with 6.1% in additional allowed positions, and 3.4% in disallowed regions (Figure S1). The small number of amino acid residues (Pro 90, His 101, Ser 105, Ile 112, Cys 115) in the disallowed region of the Ramachandran plot was selected for further molecular docking and for Root Mean Square Deviation (RMSD)-based comparison with the crystal structure of Coxsackievirus B4.

**Figure 5:**
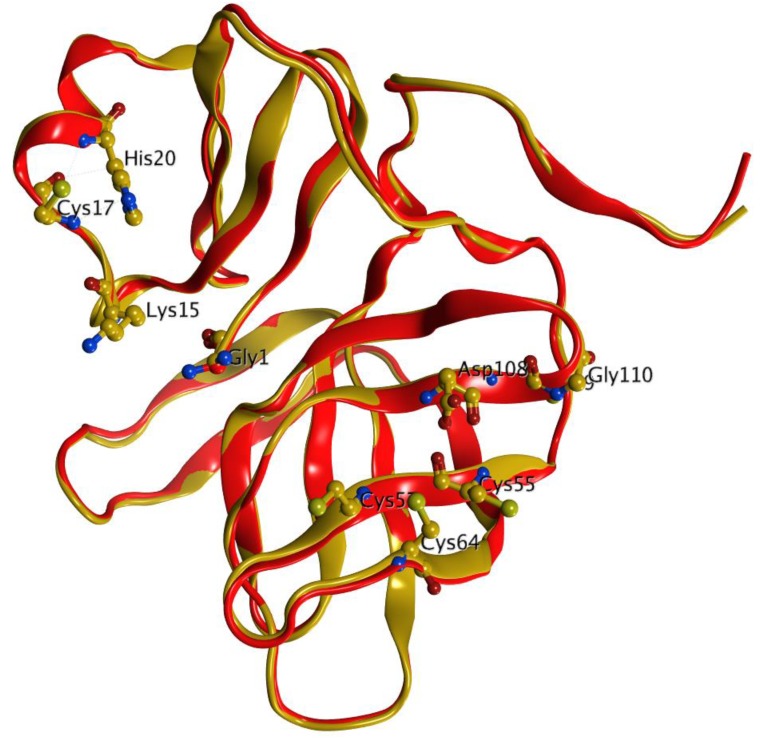
Superimposition of PV2A^pr^° (**red**) and Coxsackievirus B4 1z8r (**gold**) using the MOE program [35].

In order to estimate the overall accuracy of the model, a 3D structural comparison of the PV2A^pr^° and the equivalent Coxsackievirus B4 protein was carried out by measuring RMSD between Cα-atom positions between two proteins using the MOE version 2013 program. After flexible alignment of both structures, an RMSD of 0.08 Å was obtained, that indicated the consistency of the homology model (Figure 5). In PV2A^pr^° the predicted secondary structure consists of 27.52% *alpha* helix, 26.17% extended strand, and 29.53% random coils (Figure S2), while the predicted structure from residues Ser 95 to Ser 105 comprises a long loop of the protein. In order to gain deeper insights into the ligand–protein interaction pattern, four structurally diverse ligands were docked into the binding cavity of the modelled structure of PV2A^pr^°.

**Figure 6:**
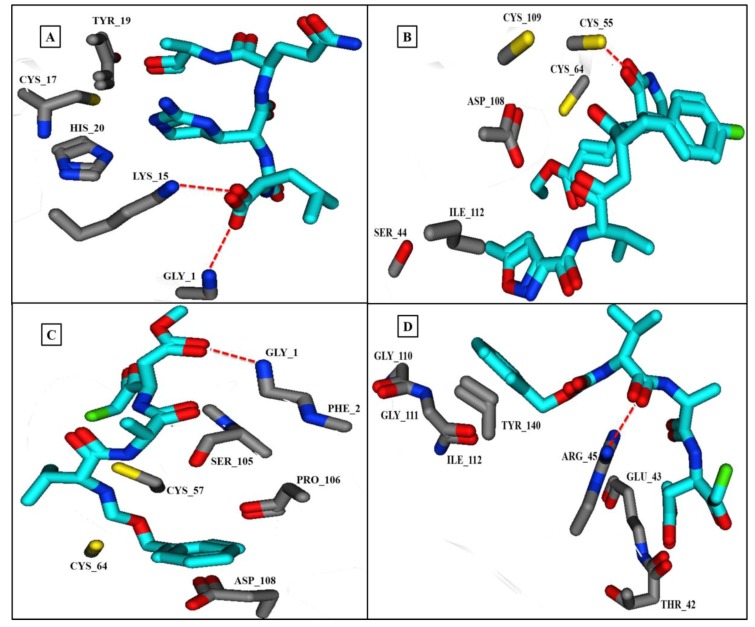
Best docked confirmations of PV2A^pr^° with four inhibitors. Dotted lines show hydrogen bonding and poses between the PV2A^pr^° and four inhibitors and the amino acid residues involved. (**A**) Elastatinal with Gly 1, Lys 15, His 20, and Cys 17; (**B**) Rupintrivir with Cys 55, Ser 66, and Cys 109; (**C**) MCPK with Gly 1, Cys 57, and Cys 64; and (**D**) z-VAD forming a cation–π interaction with residue Gly 111.

Ligands were prepared as a dataset for docking studies against PV2A^pr^° using the ChemDraw program (Figure S3). In order to remove any bias, 100 poses per ligand were generated at 5 Å. All poses were subjected to ranking based on the scoring functions to obtain best score poses. Only those binding solutions that gave maximum overlap of the GBVI/WSA dG, London dG scoring functions were selected for ligand–protein interaction analysis. Poses 91, 193, 287, and 347 were selected as examples of PV2A^pr^° binding, where elastatinal and rupintrivir showed four interactions while MCPK and z-VAD showed three interactions (Figure S4). Elastatinal formed hydrogen bonds with Gly 1, Lys 15, His 20, and Cys 17, while rupintrivir formed bonds with Cys 55, Ser 66, and Cys 109. Some interactions were also observed with Cys 64, Cys 57, Gly 110, and Gly 111 (Figure 6A–D).

### 3.5. Missense Single-Nucleotide Polymorphism Dataset

After docking analysis, a total of ten amino acids were considered to be involved in binding sites. To investigate the effects of these binding site residues on the protein, we performed missense analysis. These ten amino acids (Gly 1, Lys 15, His 20, Cys 55, Cys 57, Cys 64, Asp 108, Cys 109, and Gly 110) were then subjected to missense analysis. All amino acids were mutated to every possible mutation. As a result, a total of 190 mutations were subjected to further analysis. Missense mutations totaled ten, and were employed in a variety of in silico SNP prediction tools, in order to determine the effect of a given missense mutation on the respective gene function.

### 3.6. Missense Single-Nucleotide Polymorphisms Analysis

To obtain higher accuracy results, four in silico SNP prediction tools (PROVEAN, SNPs&GO, Meta-SNP, and PredictSNP) were employed in our study to predict the high risk missense SNPs. A total of 190 SNPs were subjected to analysis using theses algorithms. According to PROVEAN, 169 missense SNPs cause damage, while 21 are neutral (Supplementary Table S1). In the case of SNPs&GO, 113 cause damage while 77 remain neutral. According to Meta-SNP, 159 SNPs are damaging and 31 are neutral. PredictSNP showed nine SNPs (G1A, K15R, C17A, C17I, C17K, C17L, C17M, C17T, and C17V) as neutral, while 181 SNPs are pathological (Supplementary Table S2). A total of 155 SNPs were predicted to be deleterious by all four SNP prediction algorithms (Supplementary Table S2).

We categorized SNPs as damaging if they were predicted to be damaging by three or more SNP prediction algorithms in the case of Meta-SNP, while criteria of five or more for PredictSNP, and two or more for SNPs&GO, were used. After this classification, we further concentrated our analysis to select those SNPs of high risk from these four algorithms (PROVEAN, SNPs&GO, Meta-SNP, and PredictSNP), and they were then observed for their ability to confer damaging effects when using three or more prediction tools (Table S2). Out of a total of 192 SNPs, 155 were considered high risk and were subjected to further stability studies. To predict highly deleterious SNPs, these selected algorithms covered a maximum number of methods, including alignment scores, neural networks, hidden Markov models, support vector machine, and Bayesian classification.

### 3.7. Protein Stability Changes

The protein stability change upon point mutation was estimated using I-Mutant2.0, STRUM ad EASE-MM web-servers. Only highly deleterious missense SNPs were considered for the analysis. SNPs were considered as destabilizing in nature if two or more algorithms showed a decrease in stability upon mutation. Out of 155 high risk SNPs, 17 SNPs (K15L, K15P, H20E, H20L, D108C, D108E, D108F, D108I, D108L, D108M, D108P, D108Q, D108S, D108T, D108V, D108W, and D108Y) were found to be more stable than the wild type residue. The remaining SNPs resulted in decreased protein stability compared to wild type.

## 4. Discussion

PV2A^pr^° is a member of the cysteine protease superfamily of proteins and is involved in various physiological processes in virus infected cells, and is a significant target for therapeutics. However, the lack of PV2A^pr^° crystal structure is a restraint in understanding atomic details. In this study, we took advantage of PV2A^pr^° primary structural similarity to other viral proteases, specifically that from Coxsackievirus B4, and performed an in silico analysis of its structure.

**Figure 7:**
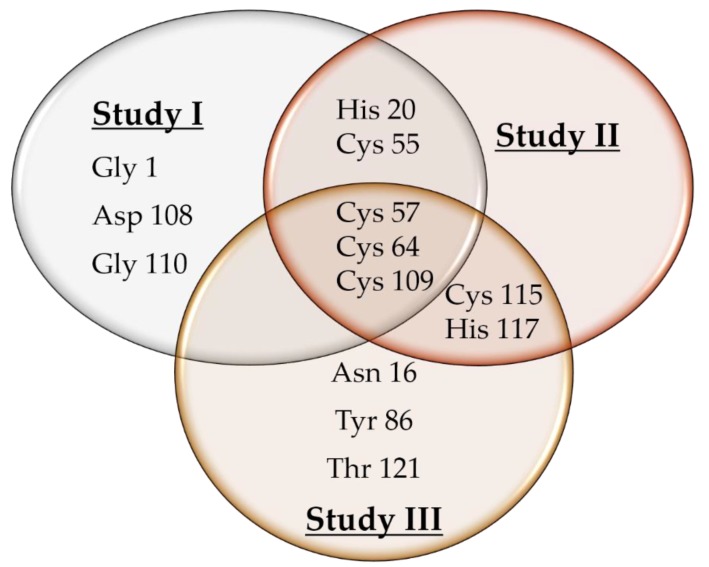
Venn diagram of the active site analysis of viral proteases. **Study I** comprises the results of recent investigation. **Study II** comprises the results analysis of Yu and Lloyd [13], while **study III** comprises the Sommergruber et al. results [58].

Computational techniques have enabled preliminary assessments of the binding capabilities of a range of potential compounds for use in viral therapy, prior to scale-up of their synthesis and further testing. To assess the binding affinity of any potentially useful compounds against their target proteins, molecular docking has been used extensively in both drug design and for screening of novel compounds. Four protease inhibitors, which may be potential therapeutic agents, were docked against PV2A^pr^° to determine any binding interactions. These inhibitors have shown promising in vitro results previously. Rupintrivir is a protease inhibitor which has successfully been used previously to inhibit replication of viruses with 3C proteases, including enterovirus 93 [54,55], and has therapeutic potential for treating PV infections, because its docking behavior with PV2A^pr^° is of a similar profile. Rupintrivir also has potent antiviral activity against all HRV serotypes tested, and has low toxicity [56]. A cation-π interaction observed between PV2A^pr^° Gly 111 and z-VAD, which is a known inhibitor of block eIF4Gase activity in vivo [57], suggests the therapeutic usefulness of this inhibitor. Similarly, MCPK which has hydrogen bonds with residues Gly 1, Cys 57, and Cys 64 (Figure 6) in PV2A^pr^°, may also be a potential therapeutic agent. MCPK is an elastase-specific inhibitor which inhibits PV2A^pr^° both in vivo and in cell-free systems [8]. The presence of fluorine in rupintrivir, which is absent from elastatinal, is thought to increase its binding capacity and its potential as an inhibitor of virus replication. Yu and Lloyd [13] (Figure 7, study II) proposed that in PV2A^pr^°, a catalytic triad comprising His 20 and Cys 109 and conserved residues, including Cys 55, Cys 57, Cys 115, and His 117, are involved in structural maintenance and catalytic activity of the protease. Sommergruber et al. (Figure 7, study III) reported that in human rhinovirus 2 proteinase 2A (HRV2 2A), Cys residues and His 114 are important in coordinating Zn^2+^ binding. Moreover, Gly 123, Gly 124, Thr 121, and Cys 101 are potentially involved in the formation of a substrate binding pocket creating the active site of His 18, Asp 35 and Cys 106. A recent study (Figure 7, study I) confirms the presence of residues His20 and Cys 109 in the active site, while Cys 55, Cys 57, and Cys 64 comprise the Zn^2+^-binding motif. Moreover, Gly 1, Asp 108, and Gly 110 are potential binding sites of the protease. These inhibitors computationally proved to be potential compounds, and could be used as drug targets against poliovirus 2A protease. Recently, a capsid inhibitor, pocapavir (V-073), have demonstrated rapid antiviral activity in an oral poliovirus vaccine human challenge model. Moreover, the 3C protease inhibitor V-7404 has suggested limited oral bioavailability [2].

The docking results indicated that residues Gly 1, His 20, Cys 109, and Cys 57 contribute significantly to strong electrostatic interactions in the active site, particularly His 20 and Cys 109. Apart from these residues, Lys 15, Cys17, Cys 55, Cys 64, Asp 108, and Gly 110 were found to be potentially interacting with the compounds. All these residues were then subjected to SNP analysis to predict the sites which could impair the docking, as well as to diversify protein function.

SNPs may affect protein dynamics by changing protein stability, disrupting salt bridges or hydrogen bonding. They can affect the binding affinity and protein interaction by altering the specificity of the protein, disrupting and blocking the active site. Highly deleterious or damaging SNP identification can be a complicated task in large-scale analyses, and involves testing thousands of SNPs in the respective genes. Using in silico approaches, isolation of disease-associated SNPs from neutral ones could significantly help in identifying disease-related mutations. In the current study, we selected highly damaging mutations in PV2A^pr^° which require further experimental testing to confirm their damaging effects.

Ten potential amino acids (Gly 1, Lys 15, Cys 17, His 20, Cys 55, Cys 57, Cys 64, Asp 108, Cys109, and Gly 110) involved in binding interactions in PV2A^pr^° were subjected to SNP analysis to obtain highly damaging missense SNPs. Except for Gly 1, all SNPs proved to be highly damaging, as Gly 1 resides in the disordered region.

The catalytic triad of PV2A^pr^° comprises His 20, Asp 38, and Cys 109, which are essential for autocatalytic activity; thus, single amino acid substitutions at these sites result in reduced catalytic activity of the protein [59]. Any mutation in the Cys 109 residue would result in the decrease of the protein stability (Table S2). Similar is the case involving His 20, except H20E and H20L SNPs, which increase the protein stability. Previously, it was reported that single amino acid mutations at the His 20 and Cys l09 residues completely lost the proteolytic activity of 2A^pr^° [60].

Complete loss of in cis and in trans activity were observed when single amino acid mutations at Cys 55 and Cys 57 were generated using site-directed mutagenesis followed by analysis of these mutant 2A^pr^° biochemical properties. Substitution of cysteine at residue 64 with serine was found to only slightly alter cis and trans activity. Also, the mutant C64N was suggested to be used to distinguish the in cis and in trans activities of 2A^pr^° [13]. Results of the current study determined that Cys 55, Cys 57, and Cys 64 are highly deleterious missense SNPs, unveiling their significance.

Mutational studies also showed that Gly 110 was hydrolyzed, and blocked eIF-4G [14]. Studies have also revealed that the mutant G110S, along with other mutants, are involved in blocking protein synthesis, as compared to wild type residues [61]. The current work also confirms the G110 residue as a highly missense SNP, as when mutated to any residue, decreases protein stability.

In light of these results, it can be concluded that along with other reported residues (His 20, Cys 55, Cys 57, Cys 64, and Cys 109), three of the residues, Lys 15, Cys 17, and Asp 108, show potential binding activity along with stability functions, unveiling their significance. Computational analysis of the protein proved it to be a better and more suitable drug target against polio. Results are very much in accordance with the author’s previous work using blood mutational samples of 2A^pr^° [62].

Amino acid variations causing changes with respect to protein stability is one of the important setbacks in proteomics, along with the computational techniques used to isolate high risk missense SNPs from significantly large SNP datasets. Important ligand–protein interactions have been identified following docking investigations in these studies to determine candidates for drug targeting, the potency of which may be increased following relatively simple structural alterations.

## 5. Conclusions

In our investigation, we have identified the most deleterious mutation in PV2A^pr^° using various bioinformatics tools. High risk missense SNPs were separated for their deleterious effect on protein function based on these tools. Hence, the in silico analysis we performed proved to be both practical and valuable for developing a thorough understanding of various diseases, thereby providing a valuable resource for pharmacogenomic approaches. Antiviral activity of these inhibitors against polio and non-polio enteroviruses using in vitro assays would help us in better understanding of protein-inhibitor interactions.

The global effort has expanded capacities to tackle other infectious diseases by building effective surveillance and immunization systems. An antiviral drug targeting viral replication, rather than the symptoms of the infection, could also be of great advantage.

## Supplementary Materials

The following are available online at http://www.mdpi.com/2073-4425/9/5/228/s1. File S1: Alignment and consensus sequence of all 553 sequences of poliovirus 2A protease using BioEdit Sequence Alignment Editor; Figure S1: Ramachandran plot analysis of PV2A^pr^°; Figure S2: Secondary structure prediction of PV2A^pr^°; Figure S3: Chemical structures of ligands prepared as dataset for docking studies against poliovirus 2A^pr^°; Figure S4: Final pose selection of inhibitors; Table S1: Missense SNPs in PV2A^pr^° predicted to be damaging or neutral using PROVEAN, SNPs&Go, MetaSNP and PredictSNP. I-Mutant2.0 was used to calculate DDG score, RI and stability; Table S2: Missense SNPs in poliovirus 2A protease predicted to be deleterious using PROVEAN, SNPs & GO Meta-SNP and Predict SNP. I-Mutant2.0, STRUM and EASE-MM web servers were used for stability analysis.
