# Supplementary material for: Structure-Function Mutational Analysis and Prediction of the Potential Impact of High Risk Non-Synonymous Single-Nucleotide Polymorphism on Poliovirus 2A Protease Stability Using Comprehensive Informatics Approaches
Source: Genes (Basel). 2018 Apr 26;9(5):228. doi: 10.3390/genes9050228 (PMC5977168; doi:10.3390/genes9050228)
Supplement: Supplementary file 1 [file genes-09-00228-s001.zip › Supplementary material/Supplementary Alignment file.docx]

Alignment and consensus sequence of all 553 sequences of poliovirus 2A protease using BioEdit Sequence Alignment Editor.

1. GFGHQNKAVYTAGYKICNYHLATQEDLQNAVNVMWNRDLLVTESRAQGTDSIARCNCNAGVYYCESRRKYYPVSFVGPTFQYMEANNYYPARYQSHMLIGHGFASPGDCGGILRCHHGVIGIITAGGEGLVAFSDIRDLYAYEEEAMEQ
2. GFGHQNKAVYTAGYKICNYHLATQEDLQNAVAVMWNRDLLVAESKAQGTDSIARCNCKTGVYYCESRRKYYPVSFVGPTFQYMEANDYYPARYQSHMLIGHGFASPGDCGGILRCQHGVVGIITAGGEGLVAFSDIRDLYAYEEEAMEQ
3. GFGHQNKAVYTAGYKICNYHLATQEDLQNAVSVMWNRDLLVTESKAQGTDSIARCNCNAGVYYCESRRKYYPVSFVGPTFQYMEANDYYPARYQSHMLIGHGFASPGDCGGILRCQHGVIGIITAGGEGLVAFSDIRDLYAYEEEAMEQ
4. GFGHQNKAVYTAGYKICNYHLATQEDLQNAVAVMWNRDLLVTESKAQGTDSIARCNCNTGVYYCESKRKYYPVSFIGPTFQYMEANDYYPARYQSHMLIGHGFASPGDCGGILRCQHGAIGIITAGGEDLVAFSDIRDLYAYEEEAMEQ
5. GFGHQNKAVYTAGYKICNYHLATQEDLQNAVAVMWNRDLLVTESKAQGTDSIARCNCNTGVYYCESKRKYYPVSFIGPTFQYMEANDYYPARYQSHMLIGHGFASPGDCGGILRCQHGVIGIITAGGEDLVAFSDIRDLYAYEEEAMEQ
6. GFGHQNKAVYTAGYKICNYHLATQEDLQNAVAVMWNRDLLVTESKAQGTDSIARCNCNTGVYYCESKRKYYPVSFIGPTFQYMEANDYYPARYQSHMLIGHGFASPGDCGGILRCQHGVIGIITAGGEDLVAFSDIRDLYAYEEEAMEQ
7. GFGHQNKAVYTAGYKICNYHLATQEDLQNAVAVMWNRDLLVTESKAQGTDLIARCNCNTGVYYCESKRKYYPVSFIGPTFQYMEANDYYPARYQSHMLIGHGFASPGDCGGILRCQHGVIGIITAGGEGLVAFSDIRDLYAYEEEAMEQ
8. GFGHQNKAVYTAGYKICNYHLATQEDLQNAVAVMWNRDLLVTESKAQGTDSIARCNCNTGVYYCESKRKYYPVSFIGPTFQYMEANDYYPARYQSHMLIGHGFASPGDCGGILRCQHGVIGIITAGGEDLVAFSDIRDLYAYEEEAMEQ
9. GFGHQNKAVYTAGYKICNYHLATQEDLQNAVAVMWNRDLLVTESKAQGTDSIARCNCNTGVYYCESKRKYYPVSFIGPTFQYMEANDYYPARYQSHMLIGHGFASPGDCGGILRCQHGVIGIITAGGEGLVAFSDIRDLYAYEEEAMEQ
10. GFGHQNKAVYTAGYKICNYHLATQEDLQNAVAVMWNRDLLVTESKAQGTDSIARCNCNTGVYYCESKRKYYPVSFIGPTFQYMEANDYYPARYQSHMLIGHGFASPGDCGGILRCQHGVIGIITAGGEGLVAFSDIRDLYAYEEEAMEQ
11. GFGHQNNAVYTAGYKICNYHLATQEDLQNAVAVMWNRDLLVTESKAQGTDSIARCNCNTGVYYCESKRKYYPVSFIGPTFQYMEANDYYPARYQSHMLIGHGFASPGDCGGILRCLHGVIGIITAGGEGLVAFSDIRDLYAYEEEAMEQ
12. GFGHQNKAVYTAGYKICNYHLATQEDLQNAVAVMWNRDLLVTESKAQGTDSIARCNCNTGVYYCESKRKYYPVTFIGPTFQYMEANDYYPARYQSHMLIGYGFASPGDCGGILRCQHGVIGIITAGGEDLVAFSDIRDLYAYEEEAMEQ
13. GFGHQNKAVYTAGYKICNYHLATQEDLQNAVAVMWNRDLLVTESKAQGTDSIARCNCYTGVYYCESKRKYYPVSFIGPTFQYMEANDYYPARYQSHMLIGHGFASPGDCGGILRCQHGVIGIITAGGEGLVAFSDIRDLYAYEEEAMEQ
14. GFGHQNKAVYTAGYKICNYHLATQEDLQNAVNVMWDRDLLVTESRSQGTDSIARCNCNTGVYYCESRRKYYPVSFVGPTFQYMEANDYYPARYQSHMLIGHGFASPGDCGGILRCRHGVIGIITAGGEGLVAFSDIRDLYAYEEEAMEQ
15. GFGHQNKAVYTAGYKICNYHLATQEDLQNAVNVMWDRDLLVTESRSQGTDSIARCNCNTGVYYCESRRKYYPVSFVDPTFQYMEANDYYPARYQSHMLIGHGFASPGDCGGILRCRHGVIGIITAGGEGLVAFSDIRDLYAYEEEAMEQ
16. GFGHQNKAVYTAGYKICNYHLATQEDLQNAVNVMWDRDLLITESRSQGTDSIARCNCNTGVYYCESRRKYYPVSFVGPTFQYMEANDYYPARYQSHMLIGHGFASPGDCGGILRCRHGVIGIITAGGEGLVAFSDIRDLYAYEEEAMEQ
17. GFGHQNKAVYTAGYKICNYHLATQEDLQNAVNVMWDRDLLITESRSQGTDSIARCNCNTGVYYCESRRKYYPVSFVGPTFQYMEANDYYPARYQSHMLIGHGFASPGDCGGILRCRHGVIGIITAGGEGLVAFSDIRDLYAYEEEAMEQ
18. GFGPQNKAVYTAGYKICNYHLATQEDLQNAVNVMWDRDLLVTESKAQGTDSIARCNCKAGVYYCESRRKYYPVSFVGPTFQYMEANDYYPARYQSHMLIGHGFASPGDCGGILRCQHGVIGIITAGGEGLVAFSDIRDLYAYEEEAMEQ
19. GFGHQNKAVYTAGYKICNYHLATQEDLQNAVNVMWDRDLLVTESKAQGTDSIARCNCKAGVYYCESRRKYYPVSFVGPTFQYMEANDYYPARYQSHMLIGHGFASPGDCGGILRCQHGVIGIITAGGEGLVAFSDIRDLYAYEEEAMEQ
20. GFGHQNKAVYTAGYKICNYHLATQEDLQNAVNVMWDRDLLVTESRSQGTDSIARCNCNTGVYYCESRRKYYPVSFVGPTFQYMEANDYYPARYQSHMLIGHGFASPGDCGGILRCRHGVIGIITAGGEGLVAFSDIRDLYAYEEEAMEQ
21. GFGHQNKAVYTAGYKICNYHLATQEDLQNAVNVMWNRDLLVTESRAQGTDSIARCNCNAGVYYCESRRKYYPVSFVGPTFQYMEANNYYPARYQSHMLIGHGFASPGDCGGILRCHHGVIGIITAGGEGLVAFTDIRDLYAYEEEAMEQ
22. GFGHQNKAVYTAGYKICNYHLATQEDLQNAVHVMWNRDLLVTESRAQGTDSIARCNCNAGVYYCESRRKYYPVSFVGPTFQYMEANNYYPARYQSHMLIGHGFASPGDCGGILRCHHGVIGIITAGGEGLVAFTDIRDLYAYEEEAMEQ
23. GFGHQNKAVYTAGYKICNYHLATQEDLQNAVNVMWNRDLLVTESKAQGTDSIARCNCNVVYYCESRRKYYPVSFVGPTFQYMEANNYYPARYQSHMLIGHGFASPGDCGGILRCHHGVIGIITAGGEGLVAFTDIRDLYAYEEEAMEQ
24. GFGHQNKAVYTAGYKICNYHLATQEDLQNAVNVMWNRDLLVTESRAQGTDSIARCNCNAGVYYCESRRKYYPVSFVGPTFQYMEANNYYPARYQSHMLIGHGFASPGDCGGILRCHHGVIGIITAGGEGLVAFTDIRDLYAYEEEAMEQ
25. GFGHQNKAVYTAGYKICNYHLATQEDLQNAVNVMWNRDLLVTESRAQGTDSIARCNCNAGVYYCESRRKYYPVSFVGPTFQYMEANNYYPARYQSHMLIGHGFASPGDCGGILRCHHGVIGIITAGGEGLVAFTDIRDLYAYEEEAMEQ
26. GFGHQNKAVYTAGYKICNYHLATQEDLQNAVNVMWNRDLLVTESRAQGTDSIARCNCNAGVYYCESRRKYYPVSFVGPTFQYMEANNYYPARYQSHMLIGHGFASPGDCGGILRCHHGVIGIITAGGEGLVAFTDIRDLYAYEEEAMEQ
27. GFGHQNKAVYTAGYKICNYHLATQEDLQNAVNVMWNRDLLVTESRAQGTDSIARCNCNAGVYYCESRRKYYPVSFVGPTFQYMEANNYYPARYQSHMLIGHGFASPGDCGGILRCHHGVIGIITAGGEGLVAFTDIRDLYAYEEEAMEQ
28. GFGHQNKAVYTAGYKICNYHLATQEDLQNAVNVMWNRDLLVTESRAQGTDSIARCNCNAGVYYCESRRKYYPVSFVGPTFQYMEANNYYPARYQSHMLIGHGFASPGDCGGILRCHHGVIGIITAGGEGLVAFTDIRDLYAYEEEAMEQ
29. GFGHQNKAVYTAGYKICNYHLATQEDLQNAVNVMWNRDLLVTESRAQGTDSIARCNCNAGVYYCESRRKYYPVSFVGPTFQYMEANNYYPARYQSHMLIGHGFASPGDCGGILRCHHGVIGIITAGGEGLVAFTDIRDLYAYEEEAMEQ
30. GFGHQNKAVYTAGYKICNYHLATQEDLQNAVNVMWNRDLLVTESRAQGTDSIARCNCNAGVYYCESRRKYYPVSFVGPTFQYMEANNYYPARYQSHMLIGHGFASPGDCGGILRCHHGVIGIITAGGEGLVAFTDIRDLYAYEEEAMEQ
31. GFGHQNKAVYTAGYKICNYHLATQEDLQNAVNVMWNRDLLVTESRAQGTDSIARCNCNAGVYYCESRRKYYPVSFVGPTFQYMEANNYYPARYQSHMLIGHGFASPGDCGGILRCHHGVIGIITAGGEGLVAFTDIRDLYAYEEEAMEQ
32. GFGHQNKAVYTAGYKICNYHLATQEDLQNAVNVMWNRDLLVTESRAQGTDSIARCNCNAGVYYCESRRKYYPVSFVGPTFQYMEANNYYPARYQSHMLIGHGFASPGDCGGILRCHHGVIGIITAGGEGLVAFTDIRDLYAYEEEAMEQ
33. GFGHQNKAVYTAGYKICNYHLATQEDLQNAVNVMWNRDLLVTESRAQGTDSIARCNCNAGVYYCESRRKYYPVSFVGPTFQYMEANNYYPARYQSHMLIGHGFASPGDCGGILRCHHGVIGIITAGGEGLVAFTDIRDLYAYEEEAMEQ
34. GFGHQNKAVYTAGYKICNYHLATQEDLQNAVNVMWNRDLLVTESRAQGTDSIARCNCNAGVYYCESRRKYYPVSFVGPTFQYMEANNYYPARYQSHMLIGHGFASPGDCGGILRCHHGVIGIITAGGEGLVAFTDIRDLYAYEEEAMEQ
35. GFGHQNKAVYTAGYKICNYHLATQEDLQNAVNVMWNRDLLVTESRAQGTDSIARCNCNAGVYYCESRRKYYPVSFVGPTFQYMEANNYYPARYQSHMLIGHGFASPGDCGGILRCHHGVIGIITAGGEGLVAFTDIRDLYAYEEEAMEQ
36. GFGHQNKAVYTAGYKICNYHLATQEDLQNAVNVMWNRDLLVTESRAQGTDSIARCNCNAGVYYCESRRKYYPVSFVGPTFQYMEANNYYPARYQSHMLIGHGFASPGDCGGILRCHHGVIGIITAGGEGLVAFTDIRDLYAYEEEAMEQ
37. GFGHQNKAVYTAGYKICNYHLATQEDLQNAVNVMWNRDLLVTESRAQGTDSIARCNCNAGVYYCESRRKYYPVSFVGPTFQYMEANNYYPARYQSHMLIGHGFASPGDCGGILRCHHGVIGIITAGGEGLVAFTDIRDLYAYEEEAMEQ
38. GFGHQNKAVYTAGYKICNYHLATQEDLQNAVNVMWNRDLLVTESRAQGTDSIARCNCNAGVYYCESRRKYYPVSFVGPTFQYMEANNYYPARYQSHMLIGHGFASPGDCGGILRCHHGVIGIITAGGEGLVAFTDIRDLYAYEEEAMEQ
39. GFGHQNKAVYTAGYKICNYHLATQEDLQNAVNVMWNRDLLVTESRAQGTDSIARCNCNAGVYYCESRRKYYPVSFVGPTFQYMEANNYYPARYQSHMLIGHGFASPGDCGGILRCHHGVIGIITAGGEGLVAFTDIRDLYAYEEEAMEQ
40. GFGHQNKAVYTAGYKICNYHLATQEDLQNAVNVMWNRDLLVTESRAQGTDSIARCNCNAGVYYCESRRKYYPVSFVGPTFQYMEANNYYPARYQSHMLIGHGFASPGDCGGILRCHHGVIGIITAGGEGLVAFTDIRDLYAYEEEAMEQ
41. GFGHQNKAVYTAGYKICNYHLATQEDLQNAVNVMWNRDLLVTESRAQGTDSIARCNCNAGVYYCESRRKYYPVSFVGPTFQYMEANNYYPARYQSHMLIGHGFASPGDCGGILRCHHGVIGIITAGGEGLVAFTDIRDLYAYEEEAMEQ
42. GFGHQNKAVYTAGYKICNYHLATQEDLQNAVNVMWNRDLLVTESRAQGTDSIARCNCNAGVYYCESRRKYYPVSFVGPTFQYMEANNYYPARYQSHMLIGHGFASPGDCGGILRCHHGVIGIITAGGEGLVAFTDIRDLYAYEEEAMEQ
43. GFGHQNKAVYTAGYKICNYHLATQEDLQNAVNVMWNRDLLVTESRAQGTDSIARCNCNAGVYYCESRRKYYPVSFVGPTFQYMEANNYYPARYQSHMLIGHGFASPGDCGGILRCHHGVIGIITAGGEGLVAFTDIRDLYAYEEEAMEQ
44. GFGHQNKAVYTAGYKICNYHLATQEDLQNAVNVMWNRDLLVTESRAQGTDSIARCNCNAGVYYCESRRKYYPVSFVGPTFQYMEANDYYPARYQSHMLIGHGFASPGDCGGILRCHHGVIGIITAGGEGLVAFTDIRDLYAYEEEAMEQ
45. GFGHQNKAVYTAGYKICNYHLATQEDLQNAVNVMWNRDLLVTESRAQGTDSIARCNCNAGVYYCESRRKYYPVSFVGPTFQYMEANNYYPARYQSHMLIGHGFASPGDCGGILRCHHGVIGIITAGGEGLVAFTDIRDLYAYEEEAMEQ
46. GFGHQNKAVYTAGYKICNYHLATQEDLQNAVNVMWNRDLLVTESRAQGTDSIARCNCNAGVYYCESRRKYYPVSFVGPTFQYMEANNYYPARYQSHVLIGHGFASPGDCGGILRCHHGVIGIITAGGEGLVAFTDIRDLYAYEEEAMEQ
47. GFGHQNKAVYTAGYKICNYHLATQEDLQNAVNVMWNRDLLVTESRAQGTDSIARCNCNAGVYYCESRRKYYPVSFVGPTFQYMEANNYYPARYQSHMLIGHGFASPGDCGGILRCHHGVIGIITAGGEGLVAFTDIRDLYAYEEEAMEQ
48. GFGHQNKAVYTAGYKICNYHLATQEDLQNAVNVMWNRDLLVTESRAQGTDSIARCNCNAGVYYCESRRKYYPVSFVGPTFQYMEANNYYPARYQSHMLIGHGFASPGDCGGILRCHHGVIGIITAGGEGLVAFTDIRDLYAYEEEAMEQ
49. GFGHQNKAVYTAGYKICNYHLATQEDLQNAVNVMWNRDLLVTESRAQGTDSIARCNCNAGVYYCESRRKYYPVSFVGPTFQYMEANNYYPARYQSHMLIGHGFASPGDCGGILRCHHGVIGIITAGGEGLVAFTDIRDLYAYEEEAMEQ
50. GFGHQNKAVYTAGYKICNYHLATQEDLQNAVNVMWNRDLLVTESRAQGTDSIARCNCNAGVYYCESRRKYYPVSFVGPTFQYMEANNYYPARYQSHMLIGHGFASPGDCGGILRCHHGVIGIITAGGEGLVAFTDIRDLYAYEEEAMEQ
51. GFGHQNKAVYTAGYKICNYHLATQEDLQNAVNVMWNRDLLVTESRAQGTDSIARCNCNAGVYYCESRRKYYPVSFVGPTFQYMEANNYYPARYQSHMLIGHGFASPGDCGGILRCHHGVIGIITAGGEGLVAFTDIRDLYAYEEEAMEQ
52. GFGHQNKAVYTAGYKICNYHLATQEDLQNAVNVMWNRDLLVTESRAQGTDSIARCNCNAGVYYCESRRKYYPVSFVGPTFQYMEANNYYPARYQSHMLIGHGFASPGDCGGILRCHHGVIGIITAGGEGLVAFTDIRDLYAYEEEAMEQ
53. GFGHQNKAVYTAGYKICNYHLATQEDLQNAVNVMWNRDLLVTESRAQGTDSIARCNCNAGVYYCESRRKYYPVSFVGPTFQYMEANNYYPARYQSHMLIGHGFASPGDCGGILRCHHGVIGIITAGGEGLVAFTDIRDLYAYEEEAMEQ
54. GFGHQNKAVYTAGYKICNYHLATQEDLQNAVNVMWNRDLLVTESRAQGTDSIARCNCNAGVYYCESRRKYYPVSFVGPTFQYMEANNYYPARYQSHMLIGHGFASPGDCGGILRCHHGVIGIITAGGEGLVAFTDIRDLYAYEEEAMEQ
55. GFGHQNKAVYTAGYKICNYHLATQEDLQNAVNVMWNRDLLVTESRAQGTDSIARCNCNAGVYYCESRRKYYPVSFVGPTFQYMEANNYYPARYQSHMLIGHGFASPGDCGGILRCHHGVIGIITAGGEGLVAFTDIRDLYAYEEEAMEQ
56. GFGHQNKAVYTAGYKICNYHLATQEDLQNAVNVMWNRDLLVTESRAQGTDSIARCNCNAGVYYCESRRKYYPVSFVGPTFQYMEANNYYPARYQSHMLIGHGFASPGDCGGILRCHHGVIGIITAGGEGLVAFTDIRDLYAYEEEAMEQ
57. GFGHQNKAVYTAGYKICNYHLATQEDLQNAVNVMWNRDLLVTESRAQGTDSIARCNCNAGVYYCESRRKYYPVSFVGPTFQYMEANNYYPARYQSHMLIGHGFASPGDCGGILRCHHGVIGIITAGGEGLVAFTDIRDLYAYEEEAMEQ
58. GFGHQNKAVYTAGYKICNYHLATQEDLQNAVNVMWNRDLLVTESRAQGTDSIARCNCNAGVYYCESRRKYYPVSFVGPTFQYMEANNYYPARYQSHMLIGHGFASPGDCGGILRCHHGVIGIITAGGEGLVAFTDIRDLYAYEEEAMEQ
59. GFGHQNKAVYTAGYKICNYHLATQEDLQNAVNVMWNRDLLVTESRAQGTDSIARCNCNAGVYYCESRRKYYPVSFVGPTFQYMEANNYYPARYQSHMLIGHGFASPGDCGGILRCHHGVIGIITAGGEGLVAFTDIRDLYAYEEEAMEQ
60. GFGHQNKAVYTAGYKICNYHLATQEDLQNAVNVMWNRDLLVTESRAQGTDSIARCNCNAGVYYCESRRKYYPVSFVGPTFQYMEANNYYPARYQSHMLIGHGFASPGDCGGILRCHHGVIGIITAGGEGLVAFTDIRDLYAYEEEAMEQ
61. GFGHQNKAVYTAGYKICNYHLATQEDLQNAVNVMWNRDLLVTESRAQGTDSIARCNCNAGVYYCESRRKYYPVSFVGPTFQYMEANNYYPARYQSHMLIGHGFASPGDCGGILRCHHGVIGIITAGGEGLVAFTDIRDLYAYEEEAMEQ
62. GFGHQNKAVYTAGYKICNYHLATQEDLQNAVNVMWNRDLLVTESRAQGTDSIARCNCNAGVYYCESRRKYYPVSFVGPTFQYMEANNYYPARYQSHMLIGHGFASPGDCGGILRCHHGVIGIITAGGEGLVAFTDIRDLYAYEEEAMEQ
63. GFGHQNKAVYTAGYKICNYHLATQEDLQNAVNVMWNRDLLVTESRAQGTDSIARCNCNAGVYYCESRRKYYPVSFVGPTFQYMEANNYYPARYQSHMLIGHGFASPGDCGGILRCHHGVIGIITAGGEGLVAFTDIRDLYAYEEEAMEQ
64. GFGHQNKAVYTAGYKICNYHLATQEDLQNAVNVMWNRDLLVTESRAQGTDSIARCNCNAGVYYCESRRKYYPVSFVGPTFQYMEANNYYPARYQSHMLIGHGFASPGDCGGILRCHHGVIGIITAGGEGLVAFTDIRDLYAYEEEAMEQ
65. GFGHQNKAVYTAGYKICNYHLATQEDLQNAVNVMWNRDLLVTESRAQGTDSIARCNCNAGVYYCESRRKYYPVSFVGPTFQYMEANNYYPARYQSHMLIGHGFASPGDCGGILRCHHGVIGIITAGGEGLVAFTDIRDLYAYEEEAMEQ
66. GFGHQNKAVYTAGYKICNYHLATQEDLQNAVNVMWNRDLLVTESRAQGTDSIARCNCNAGVYYCESRRKYYPVSFVGPTFQYMEANNYYPARYQSHMLIGHGFASPGDCGGILRCHHGVIGIITAGGEGLVAFTDIRDLYAYEEEAMEQ
67. GFGHQNKAVYTAGYKICNYHLATQEDLQNAVNVMWNRDLLVTESRAQGTDSIARCNCNAGVYYCESRRKYYPVSFVGPTFQYMEANNYYPARYQSHMLIGHGFASPGDCGGILRCHHGVIGIITAGGEGLVAFTDIRDLYAYEEEAMEQ
68. GFGHQNKAVYTAGYKICNYHLATQEDLQNAVNVMWNRDLLVTESRAQGTDSIARCNCNAGVYYCESRRKYYPVSFVGPTFQYMEANNYYPARYQSHMLIGHGFASPGDCGGILRCHHGVIGIITAGGEGLVAFTDIRDLYAYEEEAMEQ
69. GFGHQNKAVYTAGYKICNYHLATQEDLQNAVNVMWNRDLLVTESRAQGTDSIARCNCNAGVYYCESRRKYYPVSFVGPTFQYMEANNYYPARYQSHMLIGHGFASPGDCGGILRCHHGVIGIITAGGEGLVAFTDIRDLYAYEEEAMEQ
70. GFGHQNKAVYTAGYKICNYHLATQEDLQNAVNVMWNRDLLVTESRAQGTDSIARCNCNAGVYYCESRRKYYPVSFVGPTFQYMEANNYYPARYQSHMLIGHGFASPGDCGGILRCHHGVIGIITAGGEGLVAFTDIRDLYAYEEEAMEQ
71. GFGHQNKAVYTAGYKICNYHLATQEDLQNAVNVMWNRDLLVTESRAQGTDSIARCNCNAGVYYCESRRKYYPVSFVGPTFQYMEANNYYPARYQSHMLIGHGFASPGDCGGILRCHHGVIGIITAGGEGLVAFTDIRDLYAYEEEAMEQ
72. GFGHQNKAVYTAGYKICNYHLATQEDLQNAVNVMWNRDLLVTESRAQGTDSIARCNCNAGVYYCESRRKYYPVSFVGPTFQYMEANNYYPARYQSHMLIGHGFASPGDCGGILRCHHGVIGIITAGGEGLVAFTDIRDLYAYEEEAMEQ
73. GFGHQNKAVYTAGYKICNYHLATQEDLQNAVNVMWNRDLLVTESRAQGTDSIARCNCNAGVYYCESRRKYYPVSFVGPTFQYMEANNYYPARYQSHMLIGHGFASPGDCGGILRCHHGVIGIITAGGEGLVAFTDIRDLYAYEEEAMEQ
74. GFGHQNKAVYTAGYKICNYHLATQEDLQNAVNVMWNRDLLVTESRAQGTDSIARCNCNAGVYYCESRRKYYPVSFVGPTFQYMEANNYYPARYQSHMLIGHGFASPGDCGGILRCHHGVIGIITAGGEGLVAFTDIRDLYAYEEEAMEQ
75. GFGHQNKAVYTAGYKICNYHLATQEDLQNAVNVMWNRDLLVTESRAQGTDSIARCNCNAGVYYCESRRKYYPVSFVGPTFQYMEANNYYPARYQSHMLIGHGFASPGDCGGILRCHHGVIGIITAGGEGLVAFTDIRDLYAYEEEAMEQ
76. GFGHQNKAVYTAGYKICNYHLATQEDLQNAVNVMWNRDLLVTESRAQGTDSIARCNCNAGVYYCESRRKYYPVSFVGPTFQYMEANNYYPARYQSHMLIGHGFASPGDCGGILRCHHGVIGIITAGGEGLVAFTDIRDLYAYEEEAMEQ
77. GFGHQNKAVYTAGYKICNYHLATQEDLQNAVNVMWNRDLLVTESRAQGTDSIARCNCNAGVYYCESRRKYYPVSFVGPTFQYMEANNYYPARYQSHMLIGHGFASPGDCGGILRCHHGVIGIITAGGEGLVAFTDIRDLYAYEEEAMEQ
78. GFGHQNKAVYTAGYKICNYHLATQEDLQNAVNVMWNRDLLVTESRAQGTDSIARCNCNAGVYYCESRRKYYPVSFVGPTFQYMEANNYYPARYQSHMLIGHGFASPGDCGGILRCHHGVIGIITAGGEGLVAFTDIRDLYAYEEEAMEQ
79. GFGHQNKAVYTAGYKICNYHLATQEDLQNAVNVMWNRDLLVTESRAQGTDSIARCNCNAGVYYCESRRKYYPVSFVGPTFQYMEANNYYPARYQSHMLIGHGFASPGDCGGILRCHHGVIGIITAGGEGLVAFTDIRDLYAYEEEAMEQ
80. GFGHQNKAVYTAGYKICNYHLATQEDLQNAVNVMWNRDLLVTESRAQGTDSIARCNCNAGVYYCESRRKYYPVSFVGPTFQYMEANNYYPARYQSHMLIGHGFASPGDCGGILRCHHGVIGIITAGGEGLVAFTDIRDLYAYEEEAMEQ
81. GFGHQNKAVYTAGYKICNYHLATQEDLQNAVNVMWNRDLLVTESRAQGTDSIARCNCNAGVYYCESRRKYYPVSFVGPTFQYMEANNYYPARYQSHMLIGHGFASPGDCGGILRCHHGVIGIITAGGEGLVAFTDIRDLYAYEEEAMEQ
82. GFGHQNKAVYTAGYKICNYHLATQEDLQNAVNVMWNRDLLVTESRAQGTDSIARCNCNAGVYYCESRRKYYPVSFVGPTFQYMEANNYYPARYQSHMLIGHGFASPGDCGGILRCHHGVIGIITAGGEGLVAFTDIRDLYAYEEEAMEQ
83. GFGHQNKAVYTAGYKICNYHLATQEDLQNAVNVMWNRDLLVTESRAQGTDSIARCNCNAGVYYCESRRKYYPVSFVGPTFQYMEANNYYPARYQSHMLIGHGFASPGDCGGILRCHHGVIGIITAGGEGLVAFTDIRDLYAYEEEAMEQ
84. GFGHQNKAVYTAGYKICNYHLATQEDLQNAVNVMWNRDLLVTESRAQGTDSIARCNCNAGVYYCESRRKYYPVSFVGPTFQYMEANNYYPARYQSHMLIGHGFASPGDCGGILRCHHGVIGIITAGGEGLVAFTDIRDLYAYEEEAMEQ
85. GFGHQNKAVYTAGYKICNYHLATQEDLQNAVNVMWNRDLLVTESRAQGTDSIARCNCNAGVYYCESRRKYYPVSFVGPTFQYMEANNYYPARYQSHMLIGHGFASPGDCGGILRCHHGVIGIITAGGEGLVAFTDIRDLYAYEEEAMEQ
86. GFGHQNKAVYTAGYKICNYHLATQEDLQNAVNVMWNRDLLVTESRAQGTDSIARCNCNAGVYYCESRRKYYPVSFVGPTFQYMEANNYYPARYQSHMLIGHGFASPGDCGGILRCHHGVIGIITAGGEGLVAFTDIRDLYAYEEEAMEQ
87. GFGHQNKAVYTAGYKICNYHLATQEDLQNAVNVMWNRDLLVTESRAQGTDSIARCNCNAGVYYCESRRKYYPVSFVGPTFQYMEANNYYPARYQSHMLIGHGFASPGDCGGILRCHHGVIGIITAGGEGLVAFTDIRDLYAYEEEAMEQ
88. GFGHQNKAVYTAGYKICNYHLATQEDLQNAVNVMWNRDLLVTESRAQGTDSIARCNCNAGVYYCESRRKYYPVSFVGPTFQYMEANNYYPARYQSHMLIGHGFASPGDCGGILRCHHGVIGIITAGGEGLVAFTDIRDLYAYEEEAMEQ
89. GFGHQNKAVYTAGYKICNYHLATQEDLQNAVNVMWNRDLLVTESRAQGTDSIARCNCNAGVYYCESRRKYYPVSFVGPTFQYMEANNYYPARYQSHMLIGHGFASPGDCGGILRCHHGVIGIITAGGEGLVAFTDIRDLYAYEEEAMEQ
90. GFGHQNKAVYTAGYKICNYHLATQEDLQNAVNVMWNRDLLVTESRAQGTDSIARCNCNAGVYYCESRRKYYPVSFVGPTFQYMEANNYYPARYQSHMLIGHGFASPGDCGGILRCHHGVIGIITAGGEGLVAFTDIRDLYAYEEEAMEQ
91. GFGHQNKAVYTAGYKICNYHLATQEDLQNAVNVMWNRDLLVTESRAQGTDSIARCNCNAGVYYCESRRKYYPVSFVGPTFQYMEANNYYPARYQSHMLIGHGFASPGDCGGILRCHHGVIGIITAGGEGLVAFTDIRDLYAYEEEAMEQ
92. GFGHQNKAVYTAGYKICNYHLATQEDLQNAVNVMWNRDLLVTESRAQGTDSIARCNCNAGVYYCESRRKYYPVSFVGPTFQYMEANNYYPARYQSHMLIGHGFASPGDCGGILRCHHGVIGIITAGGEGLVAFTDIRDLYAYEEEAMEQ
93. GFGHQNKAVYTAGYKICNYHLATQEDLQNAVNVMWNRDLLVTESRAQGTDSIARCNCNAGVYYCESRRKYYPVSFVGPTFQYMEANNYYPARYQSHMLIGHGFASPGDCGGILRCHHGVIGIITAGGEGLVAFTDIRDLYAYEEEAMEQ
94. GFGHQNKAVYTAGYKICNYHLATQEDLQNAVNVMWNRDLLVTESRAQGTDSIARCNCNAGVYYCESRRKYYPVSFVGPTFQYMEANNYYPARYQSHMLIGHGFASPGDCGGILRCHHGVIGIITAGGEGLVAFTDIRDLYAYEEEAMEQ
95. GFGHQNKAVYTAGYKICNYHLATQEDLQNAVNVMWNRDLLVTESRAQGTDSIARCNCNAGVYYCESRRKYYPVSFVGPTFQYMEANNYYPARYQSHMLIGHGFASPGDCGGILRCHHGVIGIITAGGEGLVAFTDIRDLYAYEEEAMEQ
96. GFGHQNKAVYTAGYKICNYHLATQEDLQNAVNVMWNRDLLVTESRAQGTDSIARCNCNAGVYYCESRRKYYPVSFVGPTFQYMEANNYYPARYQSHMLIGHGFASPGDCGGILRCHHGVIGIITAGGEGLVAFTDIRDLYAYEEEAMEQ
97. GFGHQNKAVYTAGYKICNYHLATQEDLQNAVNVMWNRDLLVTESRAQGTDSIARCNCNAGVYYCESRRKYYPVSFVGPTFQYMEANNYYPARYQSHMLIGHGFASPGDCGGILRCHHGVIGIITAGGEGLVAFTDIRDLYAYEEEAMEQ
98. GFGHQNKAVYTAGYKICNYHLATQEDLQNAVNVMWNRDLLVTESRAQGTDSIARCNCNAGVYYCESRRKYYPVSFVGPTFQYMEANNYYPARYQSHMLIGHGFASPGDCGGILRCHHGVIGIITAGGEGLVAFTDIRDLYAYEEEAMEQ
99. GFGHQNKAVYTAGYKICNYHLATQEDLQNAVNVMWNRDLLVTESRAQGTDSIARCNCNAGVYYCESRRKYYPVSFVGPTFQYMEANNYYPARYQSHMLIGHGFASPGDCGGILRCHHGVIGIITAGGEGLVAFTDIRDLYAYEEEAMEQ
100. GFGHQNKAVYTAGYKICNYHLATQEDLQNAVNVMWNRDLLVTESRAQGTDSIARCNCNAGVYYCESRRKYYPVSFVGPTFQYMEANNYYPARYQSHMLIGHGFASPGDCGGILRCHHGVIGIITAGGEGLVAFTDIRDLYAYEEEAMEQ
101. GFGHQNKAVYTAGYKICNYHLATQEDLQNAVNVMWNRDLLVTESRAQGTDSIARCNCNAGVYYCESRRKYYPVSFVGPTFQYMEANNYYPARYQSHMLIGHGFASPGDCGGILRCHHGVIGIITAGGEGLVAFTDIRDLYAYEEEAMEQ
102. GFGHQNKAVYTAGYKICNYHLATQEDLQNAVNVMWNRDLLVTESRAQGTDSIARCNCNAGVYYCESRRKYYPVSFVGPTFQYMEANNYYPARYQSHMLIGHGFASPGDCGGILRCHHGVIGIITAGGEGLVAFTDIRDLYAYEEEAMEQ
103. GFGHQNKAVYTAGYKICNYHLATQEDLQNAVNVMWNRDLLVTESRAQGTDSIARCNCNAGVYYCESRRKYYPVSFVGPTFQYMEANNYYPARYQSHMLIGHGFASPGDCGGILRCHHGVIGIITAGGEGLVAFTDIRDLYAYEEEAMEQ
104. GFGHQNKAVYTAGYKICNYHLATQEDLQNAVNVMWNRDLLVTESRAQGTDSIARCNCNAGVYYCESRRKYYPVSFVGPTFQYMEANNYYPARYQSHMLIGHGFASPGDCGGILRCHHGVIGIITAGGEGLVAFTDIRDLYAYEEEAMEQ
105. GFGHQNKAVYTAGYKICNYHLATQEDLQNAVNVMWNRDLLVTESRAQGTDSIARCNCNAGVYYCESRRKYYPVSFVGPTFQYMEANNYYPARYQSHMLIGHGFASPGDCGGILRCHHGVIGIITAGGEGLVAFTDIRDLYAYEEEAMEQ
106. GFGHQNKAVYTAGYKICNYHLATQEDLQNAVNVMWNRDLLVTESRAQGTDSIARCNCNAGVYYCESRRKYYPVSFVGPTFQYMEANNYYPARYQSHMLIGHGFASPGDCGGILRCHHGVIGIITAGGEGLVAFTDIRDLYAYEEEAMEQ
107. GFGHQNKAVYTAGYKICNYHLATQEDLQNAVNVMWNRDLLVTESRAQGTDSIARCNCNAGVYYCESRRKYYPVSFVGPTFQYMEANNYYPARYQSHMLIGHGFASPGDCGGILRCHHGVIGIITAGGEGLVAFTDIRDLYAYEEEAMEQ
108. GFGHQNKAVYTAGYKICNYHLATQEDLQNAVNVMWNRDLLVTESRAQGTDSIARCNCNAGVYYCESRRKYYPVSFVGPTFQYMEANNYYPARYQSHMLIGHGFASPGDCGGILRCHHGVIGIITAGGEGLVAFTDIRDLYAYEEEAMEQ
109. GFGHQNKAVYTAGYKICNYHLATQEDLQNAVNVMWNRDLLVTESRAQGTDSIARCNCNAGVYYCESRRKYYPVSFVGPTFQYMEANNYYPARYQSHMLIGHGFASPGDCGGILRCHHGVIGIITAGGEGLVAFTDIRDLYAYEEEAMEQ
110. GFGHQNKAVYTAGYKICNYHLATQEDLQNAVNVMWNRDLLVTESRAQGTDSIARCNCNAGVYYCESRRKYYPVSFVGPTFQYMEANNYYPARYQSHMLIGHGFASPGDCGGILRCHHGVIGIITAGGEGLVAFTDIRDLYAYEEEAMEQ
111. GFGHQNKAVYTAGYKICNYHLATQEDLQNAVNVMWNRDLLVTESRAQGTDSIARCNCNAGVYYCESRRKYYPVSFVGPTFQYMEANNYYPARYQSHMLIGHGFASPGDCGGILRCHHGVIGIITAGGEGLVAFTDIRDLYAYEEEAMEQ
112. GFGHQNKAVYTAGYKICNYHLATQEDLQNAVNVMWNRDLLVTESRAQGTDSIARCNCNAGVYYCESRRKYYPVSFVGPTFQYMEANNYYPARYQSHMLIGHGFASPGDCGGILRCHHGVIGIITAGGEGLVAFTDIRDLYAYEEEAMEQ
113. GFGHQNKAVYTAGYKICNYHLATQEDLQNAVNVMWNRDLLVTESRAQGTDSIARCNCNAGVYYCESRRKYYPVSFVGPTFQYMEANNYYPARYQSHMLIGHGFASPGDCGGILRCHHGVIGIITAGGEGLVAFTDIRDLYAYEEEAMEQ
114. GFGHQNKAVYTAGYKICNYHLATQEDLQNAVNVMWNRDLLVTESRAQGTDSIARCNCNAGVYYCESRRKYYPVSFVGPTFQYMEANNYYPARYQSHMLIGHGFASPGDCGGILRCHHGVIGIITAGGEGLVAFTDIRDLYAYEEEAMEQ
115. GFGHQNKAVYTAGYKICNYHLATQEDLQNAVNVMWNRDLLVTESRAQGTDSIARCNCNAGVYYCESRRKYYPVSFVGPTFQYMEANNYYPARYQSHMLIGHGFASPGDCGGILRCHHGVIGIITAGGEGLVAFTDIRDLYAYEEEAMEQ
116. GFGHQNKAVYTAGYKICNYHLATQEDLQNAVNVMWNRDLLVTESRAQGTDSIARCNCNAGVYYCESRRKYYPVSFVGPTFQYMEANNYYPARYQSHMLIGHGFASPGDCGGILRCHHGVIGIITAGGEGLVAFTDIRDLYAYEEEAMEQ
117. GFGHQNKAVYTAGYKICNYHLATQEDLQNAVNVMWNRDLLVTESRAQGTDSIARCNCNAGVYYCESRRKYYPVSFVGPTFQYMEANNYYPARYQSHMLIGHGFASPGDCGGILRCHHGVIGIITAGGEGLVAFTDIRDLYAYEEEAMEQ
118. GYGHQNKAVYTAGYKICNYHLATQEDLQNAVNVMWNRDLIVVESKAQGTDSIARCNCNKGVYYCESRRKYYPVTFVGPTFRYMEANDYYPARYQSHMLIGHGFASPGDCGGILRCQHGVIGIITAGGEGLVAFSDIRDLYAYEEEAMEQ
119. GYGHQNKAVYTAGYKICNYHLATQEDLQNAVSVMWNRDLIVVESKAQGTDSIARCNCNTGVYYCESRRKYYPVSFVGPTFQYMEANDYYPARYQSHMLIGHGFASPGDCGGILRCQHGVIGIITAGGEGLVAFSDIRDLYAYEEEAMEQ
120. GYGHQNKAVYTAGYKICNYHLATQEDLQNAVNVMWNRDLIVVESKAQGTDSIARCNCNKGVYYCESRRKYYPVTFVGPTFQYMEANDYYPARYQSHMLIGHGFASPGDCGGILRCQHGVIGIITAGGEGLVAFSDIRDLYAYEEEAMEQ
121. GYGHQNKAVYTAGYKICNYHLATQEDLQNAVNVMWNRDLIVVESKAQGTDSIARCNCNKGVYYCESRRKYYPVTFVGPTFQYMEANDYYPARYQSHMLIGHGFASPGDCGGILRCQHGVIGIITAGGEGLVAFSDIRDLYAYEEEAMEQ
122. GYGHQNKAVYTAGYKICNYHLATQEDLQNAVSIMWNRDLIVVESRAQGIDSIARCSCNTGVYYCESRRRYYPVSFVGPTFQYMEANDYYPARYQSHMLIGHGFASPGDCGGILRCQHGVIGIITAGGEGLVAFSDIRDLYAYEEEAMEQ
123. GFGHQNKAVYTAGYKICNYHLATQEDLQNAVNVMWSRDLLVTESRAQGTDSIARCNCNAGVYYCESRRKYYPVSFVGPTFQYMEANNYYPARYQSHMLIGHGFASPGDCGGILRCHHGVIGIITAGGEGLVAFSDIRDLYAYEEEAMEQ
124. GFGHQNKAVYTAGYKICNYHLATQEDLQNAVNVMWNRDLLVTESRAQGTDSIARCNCNAGVYYCESRRKYYPVSFVGPTFQYMEANNYYPARYQSHMLIGHGFASPGDCGGILRCHHGVIGIITAGGEGLVAFTDIRDLYAYEEEAMEQ
125. GHGHQNKAVYTAGYKICNYHLATQEDLQNAVSIMWNRDLIVVESKAQGTDSIARCNCNTGVYYCESRRKYYPVSFVGPTFQYMEANDYYPARYQSHMLIGHGFASPGDCGGILRCQHGVIGIVTAGGEGLVAFSDIRDLYAYEEEAMEQ
126. GYGHQNKAVYTAGYKICNYHLATQEDLQNAVNVMWNRDLIVVESKAQGTDSIARCNCNKGVYYCESRRKYYPVTFVGPTFQYMEANDYYPARYQSHMLIGHGFASPGDCGGILRCQHGVIGIITAGGEGLVAFSDIRDLYAYEEEVMEQ
127. GYGHQNKAVYTAGYKICNYHLATQEDLQNAVNVMWNRDLIVVESKAQGTDSIARCNCNKGVYYCESRRKYYPVTFVGPTFQYMEANDYYPARYQSHMLIGHGFASPGDCGGILRCQHGVIGIITAGGEGLVAFSDIRDLYAYEEEVMEQ
128. GYGHQNKAVYTAGYKICNYHLATQEDLQNAVNVMWNRDLIVVESKAQGTDSIARCNCNKGVYYCESRRKYYPVTFVGPTFQYMEANDYYPARYQSHMLIGHGFASPGDCGGILRCQHGVIGIITAGGEGLVAFSDIRDLYAYEEEVMEQ
129. GYGHQNKAVYTAGYKICNYHLATQEDLQNAVNVMWNRDLIVVESKAQGTDSIARCNCNKGVYYCESRRKYYPVTFVGPTFQYMEANDYYPARYQSHMLIGHGFASPGDCGGILRCQHGVIGIITAGGEGLVAFSDIRDLYAYEEEVMEQ
130. GFGHQNKAVYTAGYKICNYHLATQEDLQNAVNIMWNRDLLVTESRAQGTDSIARCNCNTGVYYCESRRKYYPVSFIGPTFQYMEANDYYPARYQSHMLIGHGFASPGDCGGILRCRHGVIGIITAGGEGLVAFSDIRDLYAYEEEAMEQ
131. GHGHQNKAVYTAGYKICNYHLATQEDLQNAVSIMWNRDLIVVESKAQGTDSIARCNCSAGVYYCESRRKYYPVSFIGPTFQYMEANDYYPARYQSHMLIGHGFASPGDCGGILRCQHGVIGIVTAGGEGLVAFSDIRDLYAYEEEAMEQ
132. GHGHQNKAVYTAGYKICNYHLATQEDLQNAVSIMWNRDLIVVESKAQGTDSIARCNCSAGVYYCESRRKYYPVSFIGPTFQYMEANDYYPARYQSHMLIGHGFASPGDCGGILRCQHGVIGIVTAGGEGLVAFSDIRDLYAYEEEAMEQ
133. GHGHQNKAVYTAGYKICNYHLATQEDLQNAVSIMWNRDLIVVESKAQGTDSIARCNCSAGVYYCESRRKYYPVSFIGPTFQYMEANDYYPARYQSHMLIGHGFASPGDCGGILRCQHGVIGIVTAGGEGLVAFSDIRDLYAYEEEAMEQ
134. GHGHQNKAVYTAGYKICNYHLATQEDLQNAVSIMWNRDLIVVESKAQGTDSIARCNCSAGVYYCESRRKYYPVSFIGPTFQYMEANDYYPARYQSHMLIGHGFASPGDCGGILRCQHGVIGIVTAGGEGLVAFSDIRDLYAYEEEAMEQ
135. GHGHQNKAVYTAGYKICNYHLATQEDLQNAVSIMWNRDLIVVESKAQGTDSIARCNCSAGVYYCESRRKYYPVSFIGPTFQYMEANDYYPARYQSHMLIGHGFASPGDCGGILRCQHGVIGIVTAGGEGLVAFSDIRDLYAYEEEAMEQ
136. GHGHQNKAVYTAGYKICNYHLATQEDLQNAVSIMWNRDLIVVESKAQGTDSIARCNCSAGVYYCESRRKYYPVSFIGPTFQYMEANDYYPARYQSHMLIGHGFASPGDCGGILRCQHGVIGIVTAGGEGLVAFSDIRDLYAYEEEAMEQ
137. GHGHQNKAVYTAGYKICNYHLATQEDLQNAVSIMWNRDLIVVESKAQGTDSIARCNCSAGVYYCESRRKYYPVSFIGPTFQYMEANDYYPARYQSHMLIGHGFASPGDCGGILRCQHGVIGIVTAGGEGLVAFSDIRDLYAYEEEAMEQ
138. GHGHQNKAVYTAGYKICNYHLATQEDLQNAVSIMWNRDLIVVESKAQGTDSIARCNCSAGVYYCESRRKYYPVSFIGPTFQYMEANDYYPARYQSHMLIGHGFASPGDCGGILRCQHGVIGIVTAGGEGLVAFSDIRDLYAYEEEAMEQ
139. GHGHQNKAVYTAGYKICNYHLATQEDLQNAVSIMWNRDLIVVESKAQGTDSIARCNCSAGVYYCESRRKYYPVSFIGPTFQYMEANDYYPARYQSHMLIGHGFASPGDCGGILRCQHGVIGIVTAGGEGLVAFSDIRDLYAYEEEAMEQ
140. GHGHQNKAVYTAGYKICNYHLATQEDLQNAVSIMWNRDLIVVESKAQGTDSIARCNCSAGVYYCESRRKYYPVSFIGPTFQYMEANDYYPARYQSHMLIGHGFASPGDCGGILRCQHGVIGIVTAGGEGLVAFSDIRDLYAYEEEAMEQ
141. GHGHQNKAVYTAGYKICNYHLATQEDLQNAVSIMWNRDLIVVESKAQGTDSIARCNCSAGVYYCESRRKYYPVSFIGPTFQYMEANDYYPARYQSHMLIGHGFASPGDCGGILRCQHGVIGIVTAGGEGLVAFSDIRDLYAYEEEAMEQ
142. GHGHQNKAVYTAGYKICNYHLATQEDLQNAVSIMWNRDLIVVESKAQGTDSIARCNCSAGVYYCESRRKYYPVSFIGPTFQYMEANDYYPARYQSHMLIGHGFASPGDCGGILRCQHGVIGIVTAGGEGLVAFSDIRDLYAYEEEAMEQ
143. GHGHQNKAVYTAGYKICNYHLATQEDLQNAVSIMWNRDLIVVESKAQGTDSIARCNCSAGVYYCESRRKYYPVSFIGPTFQYMEANDYYPARYQSHMLIGHGFASPGDCGGILRCQHGVIGIVTAGGEGLVAFSDIRDLCAYEEEAMEQ
144. GHGHQNKAVYTAGYKICNYHLATQEDLQNAVSIMWNRDLIVVESKAQGTDSIARCNCSAGVYYCESRRKYYPVSFIGPTFQYMEANDYYPARYQSHMLIGHGFASPGDCGGILRCQHGVIGIVTAGGEGLVAFSDIRDLYAYEEEAMEQ
145. GHGHQNKAVYTAGYKICNYHLATQEDLQNAVSIMWNRDLIVVESKAQGTDSIARCNCSAGVYYCESRRKYYPVSFIGPTFQYMEANDYYPARYQSHMLIGHGFASPGDCGGILRCQHGVIGIITAGGEGLVAFSDIRDLYAYEEEAMEQ
146. GHGHQNKAVYTAGYKICNYHLATQEDLQNAVSIMWNRDLIVVESKAQGTDSIARCNCSAGVYYCESRRKYYPVSFIGPTFQYMEANDYYPARYQSHMLIGHGFASPGDCGGILRCQHGVIGIVTAGGEGLVAFSDIRDLYAYEEEAMEQ
147. GHGHQNKAVYTAGYKICNYHLATQEDLQNAVSIMWNRDLIVVESKAQGTDSIARCNCSAGVYYCESRRKYYPVSFIGPTFQYMEANDYYPARYQSHMLIGHGFASPGDCGGILRCQHGVIGIVTAGGEGLVAFSDIRDLYAYEEEAMEQ
148. GHGHQNKAVYTAGYKICNYHLATQEDLQSAVSIMWNRDLIVVESKAQGTDSIARCNCSAGVYYCESRRKYYPVSFIGPTFQYMEANDYYPARYQSHMLIGHGFASPGDCGGILRCQHGVIGIVTAGGEGLVAFSDIRDLYAYEEEAMEQ
149. GFGHQNKAVYTAGYKICNYHLATQEDLQNAVSVMWNRDLLVAESRALGTDSIARCSCNTGVYYCESRRKYYPVSFIGPTFQYMEANEYYPARYQSHMLIGHGFASPGDCGGILRCQHGVIGIITAGGEGLVAFSDIRDLYAYEEEAMEQ
150. GFGHQNKAVYTAGYKICNYHLATQEDLQNAVSVMWNRDLLVAESRALGTDSIARCSCNTGVYYCESRRKYYPVSFIGPTFQYMEANEYYPARYQSHMLIGHGFASPGDCGGILRCQHGVIGIITAGGEGLVAFSDIRDLYAYEEEAMEQ
151. GFGHQNKAVYTAGYKICNYHLATQEDLQNAVNVMWDRDLLVTESRAQGTDSIARCNCNAGVYYCESRRKYYPVSFVGPTFQYMEANDYYPARYQSHMLIGHGFASPGDCGGILRCNHGVIGIITAGGEGLVAFSDIRDLYAYEEEAMEQ
152. GFGHQNKAVYTAGYKICNYHLATQEDLQNAVNVMWDRDLLVTESRAQGTDSIARCNCNAGVYYCESRRKYYPVSFVGPTFQYMEANDYYPARYQSHMLIGHGFASPGDCGGILRCHHGVIGIITAGGEGLVAFADIRDLYAYEEEAMEQ
153. GFGHQNKAVYTAGYKICNYHLATQEDLQNAVNVMWDRDLLVTESRAQGTDSIARCNCNAGVYYCESRRKYYPVSFVGPTFQYMEANNYYPARYQSHMLIGHGFASPGDCGGILRCNHGVIGIITAGGEGLVAFSDIRDLYAYEEEAMEQ
154. GFGHQNKAVYTAGYKICNYHLATQEDLQNAVNVMWDRDLLVTESRAQGTDSIARCNCNAGVYYCESRRKYYPVSFVGPTFQYMEANNYYPARYQSHMLIGHGFASPGDCGGILRCHHGVIGIITAGGEGLVAFADIRDLYAYEEEAMEQ
155. GFGHQNKAVYTAGYKICNYHLATQEDLQNAVNVMWDRDLLVTESRAQGTDSIARCNCNAGVYYCESRRKYYPVSFVGPTFQYMEANNYYPARYQSHMLIGHGFASPGDCGGILRCHHGVIGIITAGGEGLVAFSDIRDLYAYEEEAMEQ
156. GFGHQNKAVYTAGYKICNYHLATQEDLQNAVNVMWDRDLLVTESRAQGTDSIARCNCNAGVYYCESRRKYYPVSFVGPTFQYMEANNYYPARYQSHMLIGHGFASPGDCGGILRCHHGVIGIITAGGEGLVAFSDIRDLYAYEEEAMEQ
157. GFGHQNKAVYTAGYKICNYHLATQEDLQNAVNVMWDRDLLVTESRAQGTDSIARCNCNAGVYYCESRRKYYPVSFVGPTFQYMEANNYYPARYQSHMLIGHGFASPGDCGGILRCHHGVIGIITAGGEGLVAFADIRDLYAYEEEAMEQ
158. GFGHQNKAVYTAGYKICNYHLATQEDLQNAVNVMWDRDLLVTESRAQGTDSIARCNCNAGVYYCESRRKYYPVSFVGPTFQYMEANNYYPARYQSHMLIGHGFASPGDCGGILRCHHGVIGIITAGGEGLVAFSDIRDLYAYEEEAMEQ
159. GFGHQNKAVYTAGYKICNYHLATQEDLQNAVNVMWDRDLLVTESRAQGTDSIARCNCNAGVYYCESRRKYYPVSFVGPTFQYMEANNYYPARYQSHMLIGHGFASPGDCGGILRCHHGVIGIITAGGEGLVAFADIRDLYAYEEEAMEQ
160. GFGHQNKAVYTAGYKICNYHLATQEDLQNAVNVMWNRDLLVTESRAQGTDSIARCNCNAGVYYCESRRKYYPVSFVGPTFQYMEANNYYPARYQSHMLIGHGFASPGDCGGILRCHHGVIGIITAGGEGLVAFSDIRDLYAYEEEAMEQ
161. GFGHQNKAVYTAGYKICNYHLATQEDLQNAVNVMWNRDLLVTESRAQGTDSIARCNCNAGVYYCESRRKYYPVSFVGPTFQYMEANNYYPARYQSHMLIGHGFASPGDCGGILRCHHGVIGIITAGGEGLVAFSDIRDLYAYEEEAMEQ
162. GFGHQNKAVYTAGYKICNYHLATQEDLQNAVNVMWNRDLLVTESRAQGTDSIARCNCNAGVYYCESRRKYYPVSFVGPTFQYMEANNYYPARYQSHMLIGHGFASPGDCGGILRCHHGVIGIITAGGEGLVAFTDIRDLYAYEEEAMEQ
163. GFGHQNKAVYTAGYKICNYHLATQEDLQNAVSVMWNRDLLVVESRALGTDSIARCSCNTGVYYCESRRKYYPVSFIGPTFQYMEANEYYPARYQSHMLIGHGFASPGDCGGILRCQHGVIGIITAGGEGLVAFSDIRDLYAYEEEAMEQ
164. GFGHQNKAVYTAGYKICNYHLATQEDLQNAVSVMWNRDLLVAESRALGTDSIARCSCNTGVYYCESRRKYYPVSFIGPTFQYMEANEYYPARYQSHMLIGHGFASPGDCGGILRCQHGVIGIITAGGEGLVAFSDIRDLYAYEEEAMEQ
165. GFGHQNKAVYTAGYKICNYHLATQEDLQNAVSVMWNRDLLVVESRALGTDSIARCSCNTGVYYCESRRKYYPVSFIGPTFQYMEANEYYPARYQSHMLIGHGFASPGDCGGILRCQHGVIGIITAGGEGLVAFSDIRDLYAYEEEAMEQ
166. GFGHQNKAVYTAGYKICNYHLATQEDLQNAVSVMWNRDLLVAESRALGTDSIARCSCNTGVYYCESRRKYYPVSFIGPTFQYMEANEYYPARYQSHMLIGHGFASPGDCGGILRCQHGVIGIITAGGEGLVAFSDIRDLYAYEEEAMEQ
167. GFGHQNKAVYTAGYKICNYHLATQEDLQNAVSVMWNRDLLVAESRALGTDSIARCSCNTGVYYCESRRKHYPVSFIGPTFQYMEANEYYPARYQSHMLIGHGFASPGDCGGILRCQHGVIGIITAGGEGLVAFSDIRDLYAYEEEAMEQ
168. GFGHQNKAVYTAGYKICNYHLATQEDLQNAVSVMWNRDLLVAESRALGTDSIARCSCNTGVYYCESRRKYYPVSFIGPTFQYMEANEYYPARYQSHMLIGHGFASPGDCGGILRCQHGVIGIITAGGEGLVAFSDIRDLYAYEEEAMEQ
169. GFGHQNKAVYTAGYKICNYHLATQEDLQNAVSVMWNRDLLVAESRALGTDSIARCSCNTGVYYCESRRKYYPVSFIGPTFQYMEANEYYPARYQSHMLIGHGFASPGDCGGILRCQHGVIGIITAGGEGLVAFSDIRDLYAYEEEAMEQ
170. GFGHQNKAVYTAGYKICNYHLATQEDLQNAVNVMWDRDLLVTESRAQGTDTIARCSCNAGVYYCESRRKYYPVSFVGPTFQYMEANDYYPARYQSHMLIGHGFASPGDCGGILRCHHGVIGIITAGGEGLVAFADIRDLYAYEEEAMEQ
171. GFGHQNKAVYTAGYKICNYHLATQEDLQNAVNVMWDRDLLVTESKAQGTDTIARCSCNAGVYYCESRRKYYPVSFVGPTFQYMEANDYYPARYQSHMLIGHGFASPGDCGGILRCHHGVIGIITAGGEGLVAFADIRDLYAYEEEAMEQ
172. GFGHQNKAVYTAGYKICNYHLATQEDLQNAVNVMWDRDLLVTESRAQGTDTIARCSCNAGVYYCESRRKYYPVSFVGPTFQYMEANDYYPARYQSHMLIGHGFASPGDCGGILRCHHGVIGIITAGGEGLVAFADIRDLYAYEEEAMEQ
173. GFGHQNKAVYTAGYKICNYHLATQEDLQNAVNVMWDRDLLVTESRAQGTDTIARCSCNAGVYYCESRRKYYPVSFVGPTFQYMEANDYYPARYQSHMLIGHGFASPGDCGGILRCHHGVIGIITAGGEGLVAFADIRDLYAYEEEAMEQ
174. GFGHQNKAVYTAGYKICNYHLATQEDLQNAVNVMWDRDLLVTESRAQGTDTIARCSCNAGVYYCESRRKYYPVSFVGPTFQYMEANDYYPARYQSHMLIGHGFASPGDCGGILRCHHGVIGIITAGGEGLVAFADIRDLYAYEEEAMEQ
175. GFGHQNKAVYTAGYKICNYHLATQEDLQNAVNVMWDRDLLVTESRAQGTDTIARCSCNAGVYYCESRRKYYPVSFVGPTFQYMEANDYYPARYQSHMLIGHGFASPGDCGGILRCHHGVIGIITAGGEGLVAFADIRDLYAYEEEAMEQ
176. GFGHQNKAVYTAGYKICNYHLATQEDLQNAVNVMWDRDLLVTESRAQGTDTIARCSCNAGVYYCESRRKYYPVSFVGPTFQYMEANDYYPARYQSHMLIGHGFASPGDCGGILRCHHGVIGIITAGGEGLVAFADIRDLYAYEEEAMEQ
177. GFGHQNKAVYTAGYKICNYHLATQEDLQNAVNVMWDRDLLVTESRAQGTDTIARCSCNAGVYYCESRRKYYPVSFVGPTFQYMEANDYYPARYQSHMLIGHGFASPGDCGGILRCHHGVIGIITAGGEGLVAFADIRDLYAYEEEAMEQ
178. GFGHQNKAVYTAGYKICNYHLATQEDLQNAVNVMWDRDLLVTESRAQGTDTIARCSCNAGVYYCESRRKYYPVSFVGPTFQYMEANDYYPARYQSHMLIGHGFASPGDCGGILRCHHGVIGIITAGGEGLVAFADIRDLYAYEEEAMEQ
179. GFGHQNKAVYTAGYKICNYHLATQEDLQNAVNVMWDRDLLVTESRAQGTDTIARCSCNAGVYYCESRRKYYPVSFVGPTFQYMEANDYYPARYQSHMLIGHGFASPGDCGGILRCHHGVIGIITAGGEGLVAFADIRDLYAYEEEAMEQ
180. GFGHQNKAVYTAGYKICNYHLATQEDLQNAVNVMWDRDLLVTESRAQGTDTIARCSCNAGVYYCESRRKYYPVSFVGPTFQYMEANDYYPARYQSHMLIGHGFASPGDCGGILRCHHGVIGIITAGGEGLVAFADIRDLYAYEEEAMEQ
181. GFGHQNKAVYTAGYKICNYHLATQEDLQNAVNVMWDRDLLVTESKAQGTDTIARCSCNAGVYYCESRRKYYPVSFVGPTFQYMEANDYYPARYQSHMLIGHGFASPGDCGGILRCHHGVIGIITAGGEGLVAFADIRDLYAYEEEAMEQ
182. GFGHQNKAVYTAGYKICNYHLATQEDLQNAVNVMWDRDLLVTESRAQGTDTIARCSCNAGVYYCESRRKYYPVSFVGPTFQYMEANDYYPARYQSHMLIGHGFASPGDCGGILRCHHGVIGIITAGGEGLVAFADIRDLYAYEEEAMEQ
183. GFGHQNKAVYTAGYKICNYHLATQEDLQNAVNVMWDRDLLVTESKAQGTDTIARCSCNAGVYYCESRRKYYPVSFVGPTFQYMEANDYYPARYQSHMLIGHGFASPGDCGGILRCHHGVIGIITAGGEGLVAFADIRDLYAYEEEAMEQ
184. GFGHQNKAVYTAGYKICNYHLATQEDLQNAVNVMWDRDLLVTESRAQGTDTIARCSCNAGVYYCESRRKYYPVSFVGPTFQYMEANDYYPARYQSHMLIGHGFASPGDCGGILRCHHGVIGIITAGGEGLVAFADIRDLYAYEEEAMEQ
185. GFGHQNKAVYTAGYKICNYHLATQEDLQNAVNVMWDRDLLVTESRAQGTDTIARCSCNAGVYYCESRRKYYPVSFVGPTFQYMEANDYYPARYQSHMLIGHGFASPGDCGGILRCHHGVIGIITAGGEGLVAFADIRDLYAYEEEAMEQ
186. GFGHQNKAVYTAGYKICNYHLATQEDLQNAVNVMWDRDLLVTESRAQGTDTIARCSCNAGVYYCESRRKYYPVSFVGPTFQYMEANDYYPARYQSHMLIGHGFASPGDCGGILRCHHGVIGIITAGGEGLVAFADIRDLYAYEEEAMEQ
187. GFGHQNKAVYTAGYKICNYHLATQEDLQNAVNVMWNRDLLVTESRAQGTDLIARCNCNAGVYYCESRRKYYPVSFVGPTFQYMEANDYYPARYQSHMLIGHGFASPGDCGGILRCHHGVIGIITAGGEGLVAFADIRDLYAYEEEAMEQ
188. GFGHQNKAVYTAGYKICNYHLATQEDLQNAVNVMWNRDLLVTESRAQGTDSIARCNCNAGVYYCESRRKYYPVSFVGPTFQYMEANDYYPARYQSHMLIGHGFASPGDCGGILRCHHGVIGIITAGGEGLVAFADIRDLYVYEEEAMEQ
189. GFGHQNKAVYTAGYKICNYHLATQEDLQNAVNVMWDRDLLVTESRAQGTDSIARCNCNAGVYYCESRRKYYPVSFIGPTFQYMEANDYYPARYQSHMLIGHGFASPGDCGGILRCHHGVIGIITAGGEGLVAFADIRDLYAYEEEAMEQ
190. GFGHQNKAVYTAGYKICNYHLATQEDLQNAVNVMWNRDLLVTESRAQGTDLIARCNCNAGVYYCESRRKYYPVSFVGPTFQYMEANDYYPARYQSHMLIGHGFASPGDCGGILRCHHGVIGIITAGGEGLVAFADIRDLYAYEEEAMEQ
191. GFGHQNKAVYTAGYKICNYHLATQEDLQNAVNVMWNRDLLVTESRAQGTDLIARCNCNAGVYYCESRRKYYPVSFVGPTFQYMEANDYYPARYQSHMLIGHGFASPGDCGGILRCHHGVIGIITAGGEGLVAFADIRDLYAYEEEAMEQ
192. GFGHQNKAVYTAGYKICNYHLATQEDLQNAVNVMWNRDLLVTESRAQGTDLIARCNCNAGVYYCESRRKYYPVSFVGPTFQYMEANDYYPARYQSHMLIGHGFASPGDCGGILRCHHGVIGIITAGGEGLVAFADIRDLYAYEEEAMEQ
193. GFGHQNKAVYTAGYKICNYHLATQEDLQNAVNIMWNRDLLVTESRAQGTDLIARCNCNAGVYYCESRRKYYPVSFVGPTFQYMEANDYYPARYQSHMLIGHGFASPGDCGGILRCHHGVIGIITAGGEGLVAFADIRDLYAYEEEAMEQ
194. GFGHQNKAVYTAGYKICNYHLATQEDLQNAVNIMWNRDLLVTESRAQGTDLIARCNCNAGVYYCESRRKYYPVSFVGPTFQYMEANDYYPARYQSHMLIGHGFASPGDCGGILRCHHGVIGIITAGGEGLVAFADIRDLYAYEEEAMEQ
195. GFGHQNKAVYTAGYKICNYHLATQEDLQNAVNVMWDRDLLVTESRAQGTDSIARCNCNAGVYYCESRRKYYPVSFVGPTFQYMEANDYYPARYQSHLLIGHGFASPGDCGGILRCHHGVIGIVTAGGEGLVAFADIRDLYAYEDEAMEQ
196. GFGHQNKAVYTAGYKICNYHLATQEDLQNAVNVMWDRDLLVTESRAQGTDSIARCNCNAGVYYCESRRKYYPVSFVGPTFQYMEANDYYPARYQSHLLIGHGFASPGDCGGILRCHHGVIGIVTAGGEGLVAFADIRDLYAYEDEAMEQ
197. GFGHQNKAVYTAGYKICNYHLATQEDLQNAVNVMWDRDLLVTESRAQGTDSIARCNCNAGVYYCESRRKYYPVSFVGPTFQYMEANDYYPARYQSHLLIGCGFASPGDCGGILRCHHGVIGIVTAGGEGLVAFADIRDLYAYEDEAMEQ
198. GFGHQNKAVYTAGYKICNYHLATQEDLQNAVNVMWDRDLLVTESRAQGTDSIARCNCNAGVYYCESRRKYYPVSFVGPTFQYMEANDYYPARYQSHLLIGCGFASPGDCGGILRCHHGVIGIVTAGGEGLVAFADIRDLYAYEDEAMEQ
199. GFGHQNKAVYTAGYKICNYHLATQEDLQNAVNVMWDRDLLVTESRAQGTDSIARCNCNAGVYYCESRRKYYPVSFVGPTFQYMEANDYYPARYQSHLLIGCGFASPGDCGGILRCHHGVIGIVTAGGEGLVAFADIRDLYAYEDEAMEQ
200. GFGHQNKAVYTAGYKICNYHLATQEDLQNAVSVMWDRDLLVTESRAQGTDSIARCNCNAGVYYCESRRKYYPVSFVGPTFQYMEANDYYPARYQSHLLIGCGFASPGDCGGILRCHHGVIGIVTAGGEGLVAFADIRDLYAYEDEAMEQ
201. GFGHQNKAVYTAGYKICNYHLATQEDLQNAVNVMWDRDLLVTESRAQGTDSIARCNCNAGVYYCESRRKYYPVSFVGPTFQYMEANDYYPARYQSHLLIGCGFASPGDCGGILRCHHGVIGIVTAGGEGLVAFADIRDLYAYEDEAMEQ
202. GFGHQNKAVYTAGYKICNYHLATQEDLQNAVNVMWDRDLLVTESRAQGTDSIARCNCNAGVYYCESRRKYYPVSFVGPTFQYMEANDYYPARYQSHLLIGCGFASPGDCGGILRCHHGVIGIVTAGGEGLVAFADIRDLYAYEEEAMEQ
203. GFGHQNKAVYTAGYKICNYHLATQEDLQNAVNVMWDRDLLVTESRAQGTDSIARCNCNAGVYYCESRRKYYPVSFVGPTFQYMEANDYYPARYQSHLLIGCGFASPGDCGGILRCHHGVIGIVTAGGEGLVAFADIRDLYAYEEEAMEQ
204. GFGHQNKAVYTAGYKICNYHLATQEDLQNAVNVMWDRDLLVTESRAQGTDSIARCNCNAGVYYCESRRKYYPVSFVGPTFQYMEANDYYPARYQSHLLIGCGFASPGDCGGILRCHHGVIGIVTAGGEGLVAFADIRDLYAYEDEAMEQ
205. GFGHQNKAVYTAGYKICNYHLATQEDLQNAVNVMWDRDLLVTESRAQGTDSIARCNCNAGVYYCESRRKYYPVSFVGPTFQYMEANDYYPARYQSHLLIGCGFASPGDCGGILRCHHGVIGIVTAGGEGLVAFADIRDLYAYEEEAMEQ
206. GFGHQNKAVYTAGYKICNYHLATQEDLQNAVNVMWDRDLLVTESRAQGTDSIARCNCNAGVYYCESRRKYYPVSFVGPTFQYMEANDYYPARYQSHLLIGCGFASPGDCGGILRCHHGVIGIVTAGGEGLVAFADIRDLYAYEEEAMEQ
207. GFGHQNKAVYTAGYKICNYHLATQEDLQNAVNVMWDRDLLVTESRAQGTDSIARCNCNAGVYYCESRRKYYPVSFVGPTFQYMEANDYYPARYQSHLLIGCGFASPGDCGGILRCHHGVIGIVTAGGEGLVAFADIRDLYAYEEEAMEQ
208. GFGHQNKAVYTAGYKICNYHLATQEDLQNAVNVMWDRDLLVTESRAQGTDSIARCNCNAGVYYCESRRKYYPVSFVGPTFQYMEANDYYPARYQSHLLIGCGFASPGDCGGILRCHHGVIGIVTAGGEGLVAFADIRDLYAYEEEAMEQ
209. GFGHQNKAVYTAGYKICNYHLATQEDLQNAVNVMWDRDLLVTESRAQGTDSIARCNCNAGVYYCESRRKYYPVSFVGPTFQYMEANDYYPARYQSHLLIGCGFASPGDCGGILRCHHGVIGIVTAGGEGLVAFADIRDLYAYEEEAMEQ
210. GFGHQNKAVYTAGYKICNYHLATQEDLQNAVNVMWDRDLLVTESRAQGTDSIARCNCNAGVYYCESRRKYYPVSFVGPTFQYMEANDYYPARYQSHLLIGCGFASPGDCGGILRCHHGVIGIVTAGGEGLVAFADIRDLYAYEEEAMEQ
211. GFGHQNKAVYTAGYKICNYHLATQEDLQNAVNVMWDRDLLVTESRAQGTDSIARCNCNAGVYYCESRRKYYPVSFVGPTFQYMEANDYYPARYQSHLLIGRGFASPGDCGGILRCHHGVIGIVTAGGEGLVAFADIRDLYAYEEEAMEQ
212. GFGHQNKAVYTAGYKICNYHLATQEDLQNAVNVMWDRDLLVTESRAQGTDSIARCNCNAGVYYCESRRKYYPVSFVGPTFQYMEANDYYPARYQSHLLIGCGFASPGDCGGILRCHHGVIGIVTAGGEGLVAFADIRDLYAYEEEAMEQ
213. GFGHQNKAVYTAGYKICNYHLATQEDLQNAVNVMWDRDLLVTESRAQGTDSIARCNCNAGVYYCESRRKYYPVSFVGPTFQYMEANDYYPARYQSHLLIGCGFASPGDCGGILRCHHGVIGIVTAGGEGLVAFADIRDLYAYEEEAMEQ
214. GFGHQNKAVYTAGYKICNYHLATQEDLQNAVNVMWDRDLLVTESRAQGTDSIARCNCNAGVYYCESRRKYYPVSFVGPTFQYMEANDYYPARYQSHLLIGCGFASPGDCGGILRCHHGVIGIVTAGGEGLVAFADIRDLCAYEEEAMEQ
215. GFGHQNKAVYTAGYKICNYHLATQEDLQNAVNIMWDRDLLVTESRAQGTDSIARCNCNAGVYYCESRRRYYPVSFVGPTFQYMEANDYYPARYQSHLLIGCGFASPGDCGGILRCHHGVIGIVTAGGEGLVAFADIRDLYAYEDEAMEQ
216. GFGHQNKAVYTAGYKICNYHLATQEDLQNAVNVMWDRDLLVTESRAQGTDSIARCNCNAGVYYCESRRKYYPVSFVGPTFQYMEANDYYPARYQSHLLIGHGFASPGDCGGILRCHHGVIGIVTAGGEGLVAFADIRDLYAYEDEAMEQ
217. GFGHQNKAVYTAGYKICNYHLATQEDLQNAVNVMWDRDLLVTESRAQGTDSIARCNCNAGVYYCESRRKYYPVSFVGPTFQYMEANDYYPARYQSHLLIGHGFASPGDCGGILRCHHGVIGIVTAGGEGLVAFADIRDLYAYEDEAMEQ
218. GFGHQNKAVYTAGYKICNYHLATQEDLQNAVNVMWDRDLLVTESRAQGTDSIARCNCNTGVYYCESRRKYYPVSFVGPTFQYMEANDYYPARYQSHLLIGHGFASPGDCGGILRCHHGVIGIVTAGGEGLVAFADIRDLYAYEDEAMEQ
219. GFGHQNKAVYTAGYKICNYHLATQEDLQNAVNVMWDRDLLVTESRAQGTDSIARCNCNAGVYYCESRRKYYPVSFVGPTFQYMEANDYYPARYQSHLLIGHGFASPGDCGGILRCHHGVIGIVTAGGEGLVAFADIRDLYAYEDEAMEQ
220. GFGHQNKAVYTAGYKICNYHLATQEDLQNAVNVMWDRDLLVTESRAQGTDSIARCNCNAGVYYCESRRKYYPVSFVGPTFQYMEANDYYPARYQSHLLIGCGFASPGDCGGILRCHHGVIGIVTAGGEGLVAFADIRDLYAYEDEAMEQ
221. GFGHQNKAVYTAGYKICNYHLATQEDLQNAVNVMWDRDLLVTESRAQGTDSIARCNCNAGVYYCESRRKYYPVSFVGPTFQYMEANDYYPARYQSHLLIGCGFASPGDCGGILRCHHGVIGIVTAGGEGLVAFADIRDLYAYEEEAMEQ
222. GFGHQNKAVYTAGYKICNYHLATQEDLQNAVNVMWDRDLLVTESRAQGTDSIARCNCNAGVYYCESRRKYYPVSFVGPTFQYMEANDYYPARYQSHLLIGHGFASPGDCGGILRCHHGVIGIITAGGEGLVAFADIRDLYAYEDEAMEQ
223. GFGHQNKAVYTAGYKICNYHLATQEDLQNAVNVMWDRDLLVTESRAQGTDSIARCNCNAGVYYCESRRKYYPVSFVGPTFQYMEANDYYPARYQSHLLIGHGFASPGDCGGILRCHHGVIGIVTAGGEGLVAFADIRDLHAYEDEAMEQ
224. GFGHQNKAVYTAGYKICNYHLATQEDLQNAVKVMWNRDLLVTESRAQGTDSIARCNCNAGVYYCESRRKYYPVSFVGPTFQYMEANNYYPARYQSHMLIGHGFASPGDCGGILRCHHGVIGIITAGGEGLVAFTDIRDLYAYEEEAMEQ
225. GFGHQNKAVYTAGYKICNYHLATQEDLQNAVNVMWDRDLLVTESRAQGTDSIARCNCNAGVYYCESRRKYYPVSFVGPTFQYMEANDYYPARYQSHLLIGHGFASPGDCGGILRCHHGVIGIVTAGGEGLVAFADIRDLYAYEDEAMEQ
226. GFGHQNKAVYTAGYKICNYHLATQEDLQNAVNVMWDRDLLVTESRAQGTDSIARCSCNAGVYYCESRRKYYPVSFVGPTFQYMEANDYYPARYQSHLLIGHGFASPGDCGGILRCHHGVIGIVTAGGEGLVAFADIRDLYAYEDEAMEQ
227. GFGHQNKAVYTAGYKICNYHLATQEDLQNAVNVMWNRDLLVTESRAQGTDLIARCNCNAGVYYCESRRKYYPVSFVGPTFQYMEANDYYPARYQSHMLIGHGFASPGDCGGVLRCHHGVIGIITAGGEGLVAFADIRDLYAYEEEAMEQ
228. GFGHQNKAVYTAGYKICNYHLATQEDLQNAVNVMWNRDLLVTESRAQGTDSIARCNCNAGVYYCESRRKYYPVSFVGPTFQYMEANNYYPARYQSHMLIGHGFASPGDCGGILRCHHGVIGIITAGGEGLVAFTDIRDLYAYEEEAMEQ
229. GFGHQNKAVYTAGYKICNYHLATQEDLQNAVNVMWNRDLLVTESKAQGTDSIARCSCNAGVYYCESRRKYYPISFVGPTFQYMEANDYYPARYQSHMLIGHGFASPGDCGGILRCQHGVIGIVTAGGEGLVAFSDIRDLYAYEEEAMEQ
230. GFGHQNKAVYTAGYKICNYHLATQEDLQNAVNVMWDRDLLVTESRAQGTDSIARCNCNAGVYYCESKRKYYPVSFVGPTFQYMEANNYYPARYQSHMLIGRGFASPGDCGGILRCHHGVIGIITAGGERLVAFSDIRDLYAYEEEAMEQ
231. GFGHQNKAVYTAGYKICNYHLATQEDLQNAVNVMWDRDLLVTESRAQGTDSIARCNCNAGVYYCESKRKYYPVSFVGPTFQYMEANNYYPARYQSHMLIGHGFASPGDCGGILRCHHGVIGIITAGGEGLVAFSDIRDLYAYEEEAMEQ
232. GFGHQNKAVYTAGYKICNYHLATQEDLQNAVNVMWDRDLLVTESRAQGTDSIARCNCNAGVYYCESKRKYYPVSFVGPTFQYMEANNYYPARYQSHMLIGHGFASPGDCGGILRCHHGVIGIITAGGEGLVAFSDIRDLYAYEEEAMEQ
233. GFGHQNKAVYTAGYKICNYHLATQEDLQNAVNVMWDRDLLVTESRAQGTDSIARCNCNAGVYYCESKRKYYPVSFVGPTFQYMEANNYYPARYQSHMLIGHGFASPGDCGGILRCHHGVIGIITAGGEGLVAFSDIRDLYAYEEEAMEQ
234. GFGHQNKAVYTAGYKICNYHLATQEDLQNAVNVMWNRDLLVTESRAQGTDSIARCNCNAGVYYCESRRKYYPVSFVGPTFQYMEANNYYPARYQSHMLIGHGFASPGDCGGILRCHHGVIGIITAGGEGLVAFSDIRDLYAYEEEAMEQ
235. GFGHQNKAVYTAGYKICNYHLATQDDLQNAVNVMWSRDLLVTESRAQGTDSIARCNCNAGVYYCESRRKYYPVSFVGPTFQYMEANNYYPARYQSHMLIGHGFASPGDCGGILRCHHGVIGIITAGGEGLVAFSDIRDLYAYEEEAMEQ
236. GFGHQNKAVYTAGYKICNYHLATQEDLQNAVNVMWNRDLLVTESRAQGTDSIARCNCNAGVYYCESRRKYYPVSFVGPTFQYMEANNYYPARYQSHMLIGHGFASPGDCGGILRCHHGVIGIITAGGEGLVAFTDIRDLYAYEEEAMEQ
237. GLGHQNKAVYTAGYKICNYHLATPEDLQNAVNIMWNRDLIVVESKAQGTDSIARCSCNAGVYYCESKRKYYPVSFVGPTFQYMEANDYYPARYQSHMLIGYGFASPGDCGGILRCHHGVIGIITAGGEGLVAFSDIRDLYAYEEEAMEQ
238. GFGHQNKAVYTAGYKICNYHLATQEDLQNAVNVMWNRDLLVTESRAQGTDSIARCNCNAGVYYCESRRKYYPVSFVGPTFQYMEANEYYPARYQSHMLIGHGFAAPGDCGGILRCQHGVIGIVTAGGEGLVAFSDIRDLYAYEDEAMEQ
239. GFGHQNKAVYTAGYKICNYHLATQEDLQNAVNVMWNRDLLVTESRAQGIDSIARCNCNAGVYYCESRRKYYPVSFVGPTFQYMEANEYYPARYQSHMLIGHGFAAPGDCGGILRCQHGVIGIVTAGGEGLVAFSDIRDLYAYEDEAMEQ
240. GFGHQNKAVYTAGYKICNYHLATQEDLQNAVNVMWNRDLLVTESRAQGTDSIARCNCNAGVYYCESRRKYYPVSFVGPTFQYMEANGYYPARYQSHMLIGHGFAAPGDCGGILRCQHGVIGIITAGGEGLVAFSDIRDLYAYEDEAMEQ
241. GFGHQNKAVYTAGYKICNYHLATQEDLQNAVNVMWNRDLLVTESRAQGTDSIARCNCNAGVYYCESRRKYYPVSFVGPTFQYMEANEYYPARYQSHMLIGHGFAAPGDCGGILRCQHGVIGIITAGGEGLVAFSDIRDLYAYEDEAMEQ
242. GFGHQNKAVYTAGYKICNYHLATQEDLQNAVNVMWNRDLLVTESRAQGTDSIARCNCNAGVYYCESRRKYYPVSFVGPTFQYMEANEYYPARYQSHMLIGHGFAAPGDCGGILRCQHGVIGIVTAGGEGLVAFSDIRDLYAYEDEAMEQ
243. GFGHQNKAVYTAGYKICNYHLATQEDLQNAVNVMWNRDLIVVESKAQGIDSIARCSCSAGVYYCESRRKYYPVSFIGPTFQYMEANDYYPARYQSHMLIGHGFASPGDCGGILRCHHGVIGIITAGGEGLVAFSDIRDLYAYEEEAMEQ
244. GLGHQNKAVYTAGYKICNYHLATPDDLQNAVNIMWNRDLIIVESKAQGIDSIARCTCNTGVYYCESKRKYYPVSFVGPTFQYMEANDYYPARYQSHMLIGHGFASPGDCGGILRCQHGVIGIITAGGEGLVAFSDIRDLYAYEEEAMEQ
245. GFGHQNKAVYTAGYKICNYHLATQDDLQNAVNVMWSRDLLVTESRAQGTDSIARCNCNAGVYYCESRRKYYPVSFVGPTFQYMEANNYYPVRYQSHMLIGHGFESPGDCGGILRCHHGVIGIITAGGEGLVAFSDIRDLYAYEEEAMEQ
246. GFGHQNKAVYTAGYKICNYHLATQDDLQNAVNVMWSRDLLVTESRAQGTDSIARCNCNAGVYYCESRRKYYPVSFVGPTFQYMEANNYYPVRYQSHMLIGHGFESPGDCGGILRCHHGVIGIITAGGEGLVAFSDIRDLYAYEEEAMEQ
247. GFGHQNKAVYTAGYKICNYHLATQDDLQNAVNVMWSRDLLVTESRAQGTDSIARCNCNAGVYYCESRRKYYPVSFVGPTFQYMEANNYYPVRYQSHMLIGHGFESPGDCGGILRCHHGVIGIITAGGEGLVAFSDIRDLYAYEEEAMEQ
248. GFGHQNKAVYTAGYKICNYHLATQDDLQNAVNVMWSRDLLVTESRAQGTDSIARCNCNAGVYYCESRRKYYPVSFVGPTFQYMEANNYYPARYQSHMLIGHGFASPGDCGGILRCHHGVIGIITAGGEGLVAFSDIRDLYAYEEEAMEQ
249. GFGHQNKAVYTAGYKICNYHLATQEDLQNAVNVMWNRDLLVTESRAQGTDSIARCNCNGVYYCESRRKYYPVSFVGPTFQYMEANNYYPARYQSHMLIGHGFASPGDCGGILRCHHGVIGIITAGGEGLVAFTDIRDLYAYEEEAMEQ
250. GFGHQNKAVYTAGYKICNYHLATQDDLQNAVNVMWSRDLLVTESRAQGTDSIARCNCNAGVYYCESRRKYYPVSFVGPTFQYMEANNYYPARYQSHMLIGHGFASPGDCGGILRCHHGVIGIITAGGEGLVAFSDIRDLYAYEEEAMEQ
251. GFGHQNKAVYTAGYKICNYHLATQEDLQNAVSVMWNRDLLVAESRALGTDSIARCSCNTGVYYCESRRKYYPVSFIGPTFQYMEANEYYPARYQSHMLIGHGFASPGDCGGILRCQHGVIGIITAGGEGLVAFSDIRDLYAYEEEAMEQ
252. GFGHQNKAVYTAGYKICNYHLATQEDLQNAVSVMWNRDLLVAESRALGTDSIARCSCNTGVYYCESRRKYYPVSFIGPTFQYMEANEYYPARYQSHMLIGHGFASPGDCGGILRCQHGVIGIITAGGEGLVAFSDIRDLYAYEEEAMEQ
253. GFGHQNKAVYTAGYKICNYHLATQEDLQNAVSVLWNRDLLVAESRAQGTDSIARCNCNTGVYYCESRRKYYPVTFIGPTFQYMEANEYYPARYQSHMLIGHGFASPGDCGGILRCQHGVIGIVTAGGEGLVAFSDIRDLYAYEEEAMEQ
254. GFGHQNKAVYTAGYKICNYHLATQEDLQNAVSVMWNRDLLVTESRAQGTDSIARCTCKAGVYYCESRRKYYPITFIGPTFQYMEANDYYPARYQSHILIGHGFASPGDCGGILRCQHGVIGIVTAGGEGLVAFSDIRDLYAYEEEAMEQ
255. GFGHQNKAVYTAGYKICNYHLATQEDLQNAVSVMWNRDLLVTESRAQGTDSIARCTCKAGVYYCESRRKYYPVTFIGPTFQYMEANDYYPARYQSHILIGHGFASPGDCGGILRCQHGVIGIITAGGEGLVAFSDIRDLYAYEEEAMEQ
256. GFGHQNKAVYTAGYKICNYHLATQEDLQNAVSVMWNRDLLVAESRAQGTDSIARCTCNVGVYYCESRRKYYPVSFVGPTFQYMEANDYYPARYQSHMLIGHGFASPGDCGGILRCQHGVIGIITAGGEGLVAFSDIRDLYAYEEEAMEQ
257. GFGHQNKAVYTAGYKICNYHLATQEDLQNAVSVMWNRDLLVAESRAQGTDSIARCTCNSGVYYCESRRKYYPVSFVGPTFQYMEANDYYPARYQSHMLIGHGFASPGDCGGILRCQHGVIGIITAGGDGLVAFSDIRDLYAYEEEAMEQ
258. GFGHQNKAVYTAGYKICNYHLATQEDLQNAVSVMWNRDLLVAESRAQGTDSIARCTCNSGVYYCESRRKYYPVSFVGPTFQYMEANDYYPARYQSHMLIGYGFASPGDCGGILRCQHGVIGIITAGGDGLVAFSDIRDLYAYEEEAMEQ
259. GFGHQNKAVYTAGYKICNYHLATQEDLQNAVSVMWNRDLLVAESRALGTDSIARCSCNTGVYYCESRRKYYPVSFIGPTFQYMEANEYYPARYQSHMLIGHGFASPGDCGGILRCQHGVIGIITAGGEGLVAFSDIRDLYAYEEEAMEQ
260. GFGHQNKAVYTAGYKICNYHLATQEDLQNAVSVMWNRDLLVAESRAQGTDSIARCNCNTGVYYCESRRKYYPVTFIGPTFQYMEANEYYPARYQSHMLIGHGFASPGDCGGILRCQHGVIGIVTAGGEGLVAFSDIRDLYAYEEEAMEQ
261. GFGHQNKAVYTAGYKICNYHLATQEDLQNAVSVMWNRDLLVAESRAQGTDSIARCNCNTGVYYCESRRKYYPVTFIGPTFQYMEANEYYPARYQSHMLIGHGFASPGDCGGILRCQHGVIGIVTAGGEGLVAFSDIRDLYAYEEEAMEQ
262. GFGHQNKAVYTAGYKICNYHLATQEDLQNAVSVMWNRDLLVAESRAQGTDSIARCNCNTGVYYCESRRKYYPVTFIGPTFQYMEANEYYPARYQSHMLIGHGFASPGDCGGILRCQHGVIGIVTAGGEGLVAFSDIRDLYAYEEEAMEQ
263. GFGHQNKAVYTAGYKICNYHLATQEDLQNAVSVLWNRDLLVAESRAQGTDSIARCNCNTGVYYCESRRKYYPVTFIGPTFQYMEANEYYPARYQSHMLIGHGFASPGDCGGILRCQHGVIGIVTAGGEGLVAFSDIRDLYAYEEEAMEQ
264. GFGHQNKAVYTAGYKICNYHLATQEDLQNAVSVMWNRDLLVAESRAQGTDSIARCNCNTGVYYCESRRKYYPVTFIGPTFQYMEANEYYPARYQSHMLIGHGFASPGDCGGILRCQHGVIGIVTAGGEGLVAFSDIRDLYAYEEEAMEQ
265. GFGHQNKAVYTAGYKICNYHLATQEDLQNAVSVMWNRDLLVAESRAQGTDSIARCNCNTGVYYCESRRKYYPVTFIGPTFQYMEANEYYPARYQSHMLIGHGFASPGDCGGILRCQHGVIGIVTAGGEGLVAFSDIRDLYAYEEEAMEQ
266. GFGHQNKAVYTAGYKICNYHLATQEDLQNAVSVMWNRDLLVAESRAQGTDSIARCNCNTGVYYCESRRKYYPVTFIGPTFQYMEANEYYPARYQSHMLIGHGFASPGDCGGILRCQHGVIGIVTAGGEGLVAFSDIRDLYAYEEEAMEQ
267. GFGHQNKAVYTAGYKICNYHLATQEDLQNAVSVMWNRDLLVAESRALGTDSIARCSCNTGVYYCESRRKYYPVSFIGPTFQYMEANEYYPARYQSHMLIGHGFASPGDCGGILRCQHGVIGIITAGGEGLVAFSDIRDLYAYEEEAMEQ
268. GFGHQNKAVYTAGYKICNYHLATQEDLQNAVSVMWSRDLLVAESRALGTDSIARCSCNTGVYYCESRRKYYPVSFIGPTFQYMEANDYYPARYQSHMLIGHGFASPGDCGGILRCQHGVIGIITAGGEGLVAFSDIRDLYAYEEEAMEQ
269. GFGHQNKAVYTAGYKICNYHLATQEDLQNAVSVMWNRDLLVAESRALGTDSIARCSCSTGVYYCESRRKYYPVSFIGPTFQYMEANEYYPARYQSHMLIGHGFASPGDCGGILRCQHGVIGIITAGGEGLVAFSDIRDLYAYEEEAMEQ
270. GFGHQNKAVYTAGYKICNYHLATQEDLQNAVSVMWNRDLLVVESRAQGTDSIARCNCNTGIYYCESRRKYYPVTFIGPTFQYMEANEYYPARYQSHMLIGHGFASPGDCGGILRCQHGVIGIVTAGGEGLVAFSDIRDLYAYEEEAMEQ
271. GFGHQNKAVYTAGYKICNYHLATQEDLQNAVSVMWNRDLLVAESKAQGTDSIARCACNTGVYYCESRRKYYPVSFVGPTFQYMEANEYYPARYQSHMLIGHGFASPGDCGGILRCQHGVIGIITAGGEGLVAFSDIRDLYAYEEEAMEQ
272. GFGHQNKAVYTAGYKICNYHLATQEDLQNAVSVMWNRDLLVAESRALGTDSIARCSCNTGVYYCESRRKYYPVSFIGPTFQYMEANEFYPARYQSHMLIGHGFASPGDCGGILRCQHGVIGIITAGGEGLVAFSDIRDLYAYEEEAMEQ
273. GFGHQNKAVYTAGYKICNYHLATQEDLQNAVSVMWNRDLLVAESKAQGTDSIARCACNTGVYYCESRRKYYPVSFVGPTFQYMEANEYYPARYQSHMLIGHGFASPGDCGGILRCQHGVIGIITAGGEGLVAFSDIRDLYAYEEEAMEQ
274. GFGHQNKAVYTAGYKICNYHLATQEDLQNAVSVMWNRDLLVTESKAQGTDSIARCACNTGVYYCESRRKYYPVSFVGPTFQYMEANEYYPARYQTHMLIGHGFASPGDCGGILRCQHGVIGIITAGGEGLVAFSDIRDLYAYEEEAMEQ
275. GFGHQNKAVYTAGYKICNYHLATQEDLQNAVSVMWNRDLLVAESKAQGTDSIARCACNTGVYYCESRRKYYPVSFVGPTFQYMEANEYYPARYQSHMLIGHGFASPGDCGGILRCQHGVIGIITAGGEGLVAFSDIRDLYAFEEEAMEQ
276. GFGHQNKAVYTAGYKICNYHLATQEDLQNAVSVMWNRDLLVAESKAQGTDSIARCACNTGVYYCESRRKYYPVSFVGPTFQYMEANEYYPARYQSHMLIGHGFASPGDCGGILRCQHGVIGIITAGGEGLVAFSDIRDLYAYEEEAMEQ
277. GFGHQNKAVYTAGYKICNYHLATQEDLQNAVSVMWNRDLLVAESKAQGTDSIARCACNTGVYYCESRRKYYPVSFVGPTFQYMEANEYYPARYQSHMLIGHGFASPGDCGGILRCQHGVIGIITAGGEGLVAFSDIRDLYAYEEEAMEQ
278. GFGHQNKAVYTAGYKICNYHLATQEDLQNAVSVMWNRDLLVAESRALGTDSIARCSCNTGVYYCESRRKYYPVSFIGPTFQYMEANEYYPARYQSHMLIGHGFASPGDCGGILRCQHGVIGIITAGGEGLVAFSDIRDLYAYEEEAMEQ
279. GFGHQNKAVYTAGYKICNYHLATQEDLQNAVSVMWNRDLLVAESRALGTDSIARCSCNTGVYYCESRRKYYPVSFIGPTFQYMEANEYYPARYQSHMLIGHGFASPGDCGGILRCQHGVIGIITAGGEGLVAFSDIRDLYAYEEEAMEQ
280. GFGHQNKAVYTAGYKICNYHLATQEDLQNAVSVMWNRDLLVAESRALGTDSIARCSCNTGVYYCESRRKYYPVSFIGPTFQYMEANEYYPARYQSHMLIGYGFASPGDCGGILRCQHGVIGIITAGGEGLVAFSDIRDLYAYEEEAMEQ
281. GFGHQNKAVYTAGYKICNYHLATQEDLQNAVSVMWNRDLLVAESRALGTDSIARCSCNTGVYYCESRRKYYPVSFIGPTFQYMEANEYYPARYQSHMLIGHGFASPGDCGGILRCQHGVIGIITAGGEGLVAFSDIRDLYAYEEEAMEQ
282. GFGHQNKAVYTAGYKICNYHLATQEDLQNAVNVMWNRDLLVAESRALGTDSIARCSCNTGVYYCESRRKYYPVSFIGPTFQYMEANEYYPARYQSHMLIGHGFASPGDCGGILRCQHGVIGIITAGGEGLVAFSDIRDLYAYEEEAMEQ
283. GFGHQNKAVYTAGYKICNYHLATQEDLQNAVSVMWNRDLLVAESRALGTDSIARCSCNTGVYYCESRRKYYPVSFIGPTFQYMEANEYYPARYQSHMLIGHGFASPGDCGGILRCQHGVIGIITAGGEGLVAFSDIRDLYAYEEEAMEQ
284. GFGYQNKAVYTAGYKICNYHLATQEDLQNAVSVMWNRDLLVAESRALGTDSIARCSCNTGVYYCESRRKYYPVSFIGPTFQYMEANEYYPARYQSHMLIGHGFASPGDCGGILRCQHGVIGIITAGGEGLVAFSDIRDLYAYEEEAMEQ
285. GFGHQNKAVYTAGYKICNYHLATQEDLQNAVSVMWNRDLLVAESRALGTDSIARCSCNTGVYYCESRRKYYPVSFIGPTFQYMEANEYYPARYQSHMLIGHGFASPGDCGGILRCQHGVIGIITAGGEGLVAFSDIRDLYAYEEEAMEQ
286. GFGHQNKAVYTAGYKICNYHLATQEDLQNAVSVMWNRDLLVAESRALGTDSIARCSCNTGVYYCESRRKYYPVSFIGPTFQYMEANEYYPARYQSHMLIGHGFASPGDCGGILRCQHGVIGIITAGGEGLVAFSDIRDLYAYEEEAMEQ
287. GFGHQNKAVYTAGYKICNYHLATQEDLQNAVSVMWNRDLLVAESRALGTDSIARCSCNTGVYYCESRRKYYPVSFIGPTFQYMEANEYYPARYQSHMLIGHGFASPGDCGGILRCQHGVIGIITAGGEGLVAFSDIRDLYAYEEEAMEQ
288. GFGHQNKAVYTAGYKICNYHLATQEDLQNAVSVMWNRDLLVAESRALGTDSIARCSCNTGVYYCESRRKYYPVSFIGPTFQYMEANEYYPARYQSHMLIGHGFASPGDCGGILRCQHGVIGIITAGGEGLVAFSDIRDLYAYEEEAMEQ
289. GFGHQNKAVYTAGYKICNYHLATQEDLQNAVSVMWNRDLLVAESRALGTDSIARCSCNTGVYYCESRRKYYPVSFIGPTFQYMEANEYYPARYQSHMLIGHGFASPGDCGGILRCQHGVIGIITAGGEGLVAFSDIRDLYAYEEEAMEQ
290. GFGHQNKAVYTAGYKICNYHLATQEDLQNAVSVMWNRDLLVAESRALGTDSIARCSCNTGVYYCESRRKYYPVSFIGPTFQYMEANEYYPARYQSHMLIGHGFASPGDCGGILRCQHGVIGIITAGGEGLVAFSDIRDLYAYEEEAMEQ
291. GFGHQNKAVYTAGYKICNYHLATQEDLQNAVSVMWNRDLLVAESRALGTDSIARCSCNTGVYYCESRRKYYPVSFIGPTFQYMEANEYYPARYQSHMLIGHGFASPGDCGGILRCQHGVIGIITAGGEGLVAFSDIRDLYAYEEEAMEQ
292. GFGHQNKAVYTAGYKICNYHLATQEDLQNAVSVMWNRDLLVAESRALGTDSIARCSCNTGVYYCESRRKYYPVSFIGPTFQYMEANEYYPARYQSHMLIGHGFASPGDCGGILRCQHGVIGIITAGGEGLVAFSDIRDLYAYEEEAMEQ
293. GFGHQNKAVYTAGYKICNYHLATQEDLQNAVSVMWNRDLLVAESRALGTDSIARCSCNTGVYYCESRRKYYPVSFIGPTFQYMEANEYYPARYQSHMLIGHGFASPGDCGGILRCQHGVIGIITAGGEGLVAFSDIRDLYAYEEEAMEQ
294. GFGHQNKAVYTAGYKICNYHLATQEDLQNAVSVMWNRDLLVAESRALGTDSIARCSCNTGVYYCESRRKYYPVSFIGPTFQYMEANEYYPARYQSHMLIGHGFASPGDCGGILRCQHGVIGIITAGGEGLVAFSDIRDLYAYEEEAMEQ
295. GFGHQNKAVYTAGYKICNYHLATQEDLQNAVSVMWNRDLLVAESRALGTDSIARCSCNTGVYYCESRRKYYPVSFIGPTFQYMEANEYYPARYQSHMLIGHGFASPGDCGGILRCQHGVIGIITAGGEGLVAFSDIRDLYAYEEEAMEQ
296. GFGHQNKAVYTAGYKICNYHLATQEDLQNAVSVMWNRDLLVAESRALGTDSIARCSCNTGVYYCESRRKYYPVSFIGPTFQYMEANEYYPARYQSHMLIGHGFASPGDCGGILRCQHGVIGIITAGGEGLVAFSDIRDLYAYEEEAMEQ
297. GFGHQNKAVYTAGYKICNYHLATQEDLQNAVSVMWNRDLLVAESRALGTDSIARCSCNTGVYYCESRRKYYPVSFIGPTFQYMEANEYYPARYQSHMLIGHGFASPGDCGGILRCQHGVIGIITAGGEGLVAFSDIRDLYAYEEEAMEQ
298. GFGHQNKAVYTAGYKICNYHLATQEDLQNAVSVMWNRDLLVAESRALGTDSIARCSCNTGVYYCESRRKYYPVSFIGPTFQYMEANEYYPARYQSHMLIGHGFASPGDCGGILRCQHGVIGIITAGGEGLVAFSDIRDLYAYEEEAMEQ
299. GFGHQNKAVYTAGYKICNYHLATQEDLQNAVSVMWNRDLLVAESRALGTDSIARCSCNTGVYYCESRRKYYPVSFIGPTFQYMEANEYYPARYQSHMLIGHGFASPGDCGGILRCQHGVIGIITAGGEGLVAFSDIRDLYAYEEEAMEQ
300. GFGHQNKAVYTAGYKICNYHLATQEDLQNAVSVMWNRDLLVAESRALGTDSIARCSCNTGVYYCESRRKYYPVSFIGPTFQYMEANEYYPARYQSHMLIGHGFASPGDCGGILRCQHGVIGIITAGGEGLVAFSDIRDLYAYEEEAMEQ
301. GFGHQNKAVYTAGYKICNYHLATQEDLQNAVSVMWNRDLLVAESRALGTDSIARCSCNTGVYYCESRRKYYPVSFIGPTFQYMEANEYYPARYQSHMLIGHGFASPGDCGGILRCQHGVIGIITAGGEGLVAFSDIRDLYAYEEEAMEQ
302. GFGHQNKAVYTAGYKICNYHLATQEDLQNAVSVMWNRDLLVAESRALGTDSIARCSCNTGVYYCESRRKYYPVSFIGPTFQYMEANEYYPARYQSHMLIGHGFASPGDCGGILRCQHGVIGIITAGGEGLVAFSDIRDLYAYEEEAMEQ
303. GFGHQNKAVYTAGYKICNYHLATQEDLQNAVSVMWNRDLLVAESRALGTDSIARCSCNTGVYYCESRRKYYPVSFIGPTFQYMEANEYYPARYQSHMLIGHGFASPGDCGGILRCQHGVIGIITAGGEGLVAFSDIRDLYAYEEEAMEQ
304. GFGHQNKAVYTAGYKICNYHLATQEDLQNAVSVMWNRDLLVAESRALGTDSIARCSCNTGVYYCESRRKYYPVSFIGPTFQYMEANEYYPARYQSHMLIGHGFASPGDCGGILRCQHGVIGIITAGGEGLVAFSDIRDLYAYEEEAMEQ
305. GFGHQNKAVYTAGYKICNYHLATQEDLQNAVSVMWNRDLLVAESRALGTDSIARCSCNTGVYYCESRRKYYPVSFIGPTFQYMEANEYYPARYQSHMLIGHGFASPGDCGGILRCQHGVIGIITAGGEGLVAFSDIRDLYAYEEEAMEQ
306. GFGHQNKAVYTAGYKICNYHLATQEDLQNAVSVMWNRDLLVAESRALGTDSIARCSCNTGVYYCESRRKYYPVSFIGPTFQYMEANEYYPARYQSHMLIGHGFASPGDCGGILRCQHGVIGIITAGGEGLVAFSDIRDLYAYEEEAMEQ
307. GFGHQNKAVYTAGYKICNYHLATQEDLQNAVSVMWNRDLLVAESRALGTDSIARCSCNTGVYYCESRRKYYPVSFIGPTFQYMEANEYYPARYQSHMLIGHGFASPGDCGGILRCQHGVIGIITAGGEGLVAFSDIRDLYAYEEEAMEQ
308. GFGHQNKAVYTAGYKICNYHLATQEDLQNAVSVMWNRDLLVAESRALGTDSIARCSCNTGVYYCESRRKYYPVSFIGPTFQYMEANEYYPARYQSHMLIGHGFASPGDCGGILRCQHGVIGIITAGGEGLVAFSDIRDLYAYEEEAMEQ
309. GFGHQNKAVYTAGYKICNYHLATQEDLQNAVSVMWNRDLLVAESRALGTDSIARCSCNTGVYYCESRRKYYPVSFIGPTFQYMEANEYYPARYQSHMLIGHGFASPGDCGGILRCQHGVIGIITAGGEGLVAFSDIRDLYAYEEEAMEQ
310. GFGHQNKAVYTAGYKICNYHLATQEDLQNAVSVMWNRDLLVAESRALGTDSIARCSCNTGVYYCESRRKYYPVSFIGPTFQYMEANEYYPARYQSHMLIGHGFASPGDCGGILRCQHGVIGIITAGGEGLVAFSDIRDLYAYEEEAMEQ
311. GFGHQNKAVYTAGYKICNYHLATQEDLQNAVSVMWNRDLLVAESRALGTDSIARCSCNTGVYYCESRRKYYPVSFIGPTFQYMEANEYYPARYQSHMLIGHGFASPGDCGGILRCQHGVIGIITAGGEGLVAFSDIRDLYAYEEEAMEQ
312. GFGHQNKAVYTAGYKICNYHLATQEDLQNAVSVMWNRDLLVAESRALGTDSIARCSCNTGVYYCESRRKYYPVSFIGPTFQYMEANEYYPARYQSHMLIGHGFASPGDCGGILRCQHGVIGIITAGGEGLVAFSDIRDLYAYEEEAMEQ
313. GFGHQNKAVYTAGYKICNYHLATQEDLQNAVSVMWNRDLLVAESRALGTDSIARCSCNTGVYYCESRRKYYPVSFIGPTFQYMEANEYYPARYQSHMLIGHGFASPGDCGGILRCQHGVIGIITAGGEGLVAFSDIRDLYAYEEEAMEQ
314. GFGHQNKAVYTAGYKICNYHLATQEDLQNAVSVMWNRDLLVAESRALGTDSIARCSCNTGVYYCESRRKYYPVSFIGPTFQYMEANEYYPARYQSHMLIGHGFASPGDCGGILRCQHGVIGIITAGGEGLVAFSDIRDLYAYEEEAMEQ
315. GFGHQNKAVYTAGYKICNYHLATQEDLQNAVSVMWNRDLLVAESRALGTDSIARCSCNTGVYYCESRRKYYPVSFIGPTFQYMEANEYYPARYQSHMLIGHGFASPGDCGGILRCQHGVIGIITAGGEGLVAFSDIRDLYAYEEEAMEQ
316. GFGHQNKAVYTAGYKICNYHLATQEDLQNAVSVMWNRDLLVAESRALGTDSIARCSCNTGVYYCESRRKYYPVSFIGPTFQYMEANEYYPARYQSHMLIGHGFASPGDCGGILRCQHGVIGIITAGGEGLVAFSDIRDLYAYEEEAMEQ
317. GFGHQNKAVYTAGYKICNYHLATQEDLQNAVSVMWNRDLLVAESRALGTDSIARCSCNTGVYYCESRRKYYPVSFIGPTFQYMEANEYYPARYQSHMLIGHGFASPGDCGGILRCQHGVIGIITAGGEGLVAFSDIRDLYAYEEEAMEQ
318. GFGHQNKAVYTAGYKICNYHLATQEDLQNAVSVMWNRDLLVAESRALGTDSIARCSCNTGVYYCESRRKYYPVSFIGPTFQYMEANEYYPARYQSHMLIGHGFASPGDCGGILRCQHGVIGIITAGGEGLVAFSDIRDLYAYEEEAMEQ
319. GFGHQNKAVYTAGYKICNYHLATQEDLQNAVSVMWNRDLLVAESRALGTDSIARCSCNTGVYYCESRRKYYPVSFIGPTFQYMEANEYYPARYQSHMLIGHGFASPGDCGGILRCQHGVIGIITAGGEGLVAFSDIRDLYAYEEEAMEQ
320. GFGHQNKAVYTAGYKICNYHLATQEDLQNAVSVMWNRDLLVAESRALGTDSIARCSCNTGVYYCESRRKYYPVSFIGPTFQYMEANEYYPARYQSHMLIGHGFASPGDCGGILRCQHGVIGIITAGGEGLVAFSDIRDLYAYEEEAMEQ
321. GFGHQNKAVYTAGYKICNYHLATQEDLQNAVSVMWNRDLLVAESRALGTDSIARCSCNAGVYYCESRRKYYPVSFIGPTFQYMEANEYYPARYQSHMLIGHGFASPGDCGGILRCQHGVIGIITAGGEGLVAFSDIRDLYAYEEEAMEQ
322. GFGHQNKAVYTAGYKICNYHLATQEDLQNAVSVMWNRDLLVAESRALGTDSIARCSCNTGVYYCESRRKYYPVSFIGPTFQYMEANEYYPARYQSHMLIGHGFASPGDCGGILRCQHGVIGIITAGGEGLVAFSDIRDLYAYEEEAMEQ
323. GFGHQNKAVYTAGYKICNYHLATQEDLQNAVSVMWNRDLLVAESRALGTDSIARCSCNTGVYYCESRRKYYPVSFIGPTFQYMEANEYYPARYQSHMLIGHGFASPGDCGGILRCQHGVIGIITAGGEGLVAFSDIRDLYAYEEEAMEQ
324. GFGHQNKAVYTAGYKICNYHLATQEDLQNAVSVMWNRDLLVAESRALGTDSIARCSCNTGVYYCESRRKYYPVSFIGPTFQYMEANEYYPARYQSHMLIGHGFASPGDCGGILRCQHGVIGIITAGGEGLVAFSDIRDLYAYEEEAMEQ
325. GFGHQNKAVYTAGYKICNYHLATQEDLQNAVSVMWSRDLLVAESRALGTDSIARCSCNTGVYYCESRRKYYPVSFIGPTFQYMEANEYYPARYQSHMLIGHGFASPGDCGGILRCQHGVIGIITAGGEGLVAFSDIRDLYAYEEEAMEQ
326. GFGHQNKAVYTAGYKICNYHLATQEDLQNAVSVMWNRDLLVAESRALGTDSIARCSCNTGVYYCESRRKYYPVSFIGPTFQYMEANEYYPARYQSHMLIGHGFASPGDCGGILRCQHGVIGIITAGGEGLVAFSDIRDLYAYEEEAMEQ
327. GFGHQNKAVYTAGYKICNYHLATQEDLQNAVSVMWNRDLLVAESRALGTDSIARCSCNAGVYYCESRRKYYPVSFIGPTFQYMEANEYYPARYQSHMLIGHGFASPGDCGGILRCQHGVIGIITAGGEGLVAFSDIRDLYAYEEEAMEQ
328. GFGHQNKAVYTAGYKICNYHLATQEDLQNAVSVMWNRDLLVAESRALGTDSIARCSCNTGVYYCESRRKYYPVSFIGPTFQYMEANEYYPARYQSHMLIGHGFASPGDCGGILRCQHGVIGIITAGGEGLVAFSDIRDLYAYEEEAMEQ
329. GFGHQNKAVYTAGYKICNYHLATQEDLQNAVSVMWNRDLLVAESRALGTDSIARCSCNTGVYYCESRRKYYPVSFIGPTFQYMEANEYYPARYQSHMLIGHGFASPGDCGGILRCQHGVIGIITAGGEGLVAFSDIRDLYAYEEEAMEQ
330. GFGHQNKAVYAAGYKICNYHLATQEDLQNAVSVMWNRDLLVTESRAHGIDSIARCSCNTGVYYCESRRKYYPVSFIGPTFQYMEANEYYPARYQSHMLIGHGFASPGDCGGILRCQHGVIGIITAGGEGLVAFSDIRDLYAYEEEAMEQ
331. GFGHQNKAVYTAGYKICNYHLATQEDLQNAVSVMWNRDLLVAESRALGTDSIARCSCNTGVYYCESRRKYYPVSFIGPTFQYMEANEYYPARYQSHMLIGHGFASPGDCGGILRCQHGVIGIITAGGEGLVAFSDIRDLYAYEEEAMEQ
332. GFGHQNKAVYTAGYKICNYHLATQEDLQNAVSVMWNRDLLVAESRALGTDSIARCSCNTGVYYCESRRKYYPVSFIGPTFQYMEANEYYPARYQSHMLIGHGFASPGDCGGILRCQHGVIGIITAGGEGLVAFSDIRDLYAYEEEAMEQ
333. GFGHQNKAVYTAGYKICNYHLATQEDLQNAVSVMWNRDLLVAESRALGTDSIARCSCNTGVYYCESRRKYYPVSFIGPTFQYMEANEYYPARYQSHMLIGHGFASPGDCGGILRCQHGVIGIITAGGEGLVAFSDIRDLYAYEEEAMEQ
334. GFGHQNKAVYTAGYKICNYHLATQEDLQNAVSVMWNRDLLVAESRALGTDSIARCSCNTGVYYCESRRKYYPVSFIGPTFQYMEANEYYPARYQSHMLIGHGFASPGDCGGILRCQHGVIGIITAGGEGLVAFSDIRDLYAYEEEAMEQ
335. GFGHQNKAVYTAGYKICNYHLATQEDLQNAVSVMWNRDLLVAESRALGTDSIARCSCNTGVYYCESRRKYYPVSFIGPTFQYMEANEYYPARYQSHMLIGHGFASPGDCGGILRCQHGVIGIITAGGEGLVAFSDIRDLYAYEEEAMEQ
336. GFGHQNKAVYTAGYKICNYHLATQEDLQNAVSVMWNRDLLVAESRALGTDSIARCSCNTGVYYCESRRKYYPVSFIGPTFQYMEANEYYPARYQSHMLIGHGFASPGDCGGILRCQHGVIGIITAGGEGLVAFSDIRDLYAYEEEAMEQ
337. GFGHQNKAVYTAGYKICNYHLATQEDLQNAVSVMWNRDLLVAESRALGTDSIARCSCNTGVYYCESRRKYYPVSFIGPTFQYMEANEYYPARYQSHMLIGHGFASPGDCGGILRCQHGVIGIITAGGEGLVAFSDIRDLYAYEEEAMEQ
338. GFGHQNKAVYTAGYKICNYHLATQEDLQNAVSVMWNRDLLVAESRALGTDSIARCSCNTGVYYCESRRKYYPVSFIGPTFQYMEANEYYPARYQSHMLIGHGFASPGDCGGILRCQHGVIGIITAGGEGLVAFSDIRDLYAYEEEAMEQ
339. GFGHQNKAVYTAGYKICNYHLATQEDLQNAVSVMWNRDLLVAESRALGTDSIARCSCNTGVYYCESRRKYYPVSFIGPTFQYMEANEYYPARYQSHMLIGHGFASPGDCGGILRCQHGVIGIITAGGEGLVAFSDIRDLYAYEEEAMEQ
340. GFGHQNKAVYTAGYKICNYHLATQEDLQNAVSVMWNRDLLVAESRALGTDSIARCSCNTGVYYCESRRKYYPVSFIGPTFQYMEANEYYPARYQSHMLIGHGFASPGDCGGILRCQHGVIGIITAGGEGLVAFSDIRDLYAYEEEAMEQ
341. GFGHQNKAVYTTGYKICNYHLATTEDLQNAVSIMWNRDLLIAKSNAQGTDSIARCNCRAGVYYCESRRKYYPVSFVGPTFQYMEANEYYPARYQSHMLIGHGFASPGDCGGILRCQHGVIGIVTAGGEGLVAFSDIRDLHAYEEEAMEQ
342. GFGHQNKAVYTTGYKICNYHLATTEDLQNAVSIMWNRDLLIAKSNAQGTDSIARCNCRAGVYYCESRRKYYPVSFVGPTFQYMEANEYYPARYQSHMLIGHGFASPGDCGGILRCQHGVIGIVTAGGEGLVAFSDIRDLHAYEEEAMEQ
343. GFGHQNKAVYTAGYKICNYHLATQEDLQNAVSVMWNRDLLVAESRALGTDSIARCSCNTGVYYCESRRKYYPVSFIGPTFQYMEANEYYPARYQSHMLIGHGFASPGDCGGILRCQHGVIGIITAGGEGLVAFSDIRDLYAYEEEAMEQ
344. GFGHQNKAVYTAGYKICNYHLATQEDLQNAVSVLWNRDLLVAESKAQGTDSIARCTCNTGVYYCESRRKYYPISFVGPTFQYMEANDYYPARYQSHMLIGHGFASPGDCGGILRCQHGVIGIITAGGEGLVAFSDIRDLYAYEEEAMEQ
345. GFGHQNKAVYTAGYKICNYHLATQEDLQNAVSVLWNRDLLVAESKAQGTDSIARCTCNTGVYYCESRRKYYPISFVGPTFQYMEANNYYPARYQSHMLIGHGFASPGDCGGILRCQHGVIGIITAGGEGLVAFSDIRDLYAYEEEAMEQ
346. GFGHQNKAVYTAGYKICNYHLATQEDLQNAVSVMWNRDLLVAESRALGTDSIARCSCNTGVYYCESRRKYYPVSFIGPTFQYMEANEYYPARYQSHMLIGHGFASPGDCGGILRCQHGVIGIITAGGEGLVAFSDIRDLYAYEEEAMEQ
347. GFGHQNKAVYTAGYKICNYHLATQEDLQNAVSVMWNRDLLVAESRALGTDSIARCSCNTGVYYCESRRKYYPVSFIGPTFQYMEANEYYPARYQSHMLIGHGFASPGDCGGILRCQHGVIGIITAGGEGLVAFSDIRDLYAYEEEAMEQ
348. GFGHQNKAVYTAGYKICNYHLATQEDLQNAVSVMWNRDLLVAESRALGTDLIARCSCNTGVYYCESRRKYYPVSFIGPTFQYMEANEYYPARYQSHMLIGHGFASPGDCGGILRCQHGVIGIITAGGEGLVAFSDIRDLYAYEEEAMEQ
349. GFGHQNKAVYTAGYKICNYHLATQEDLQNAVSVMWNRDLLVAESRALGTDSIARCSCNTGVYYCESRRKYYPVSFIGPTFQYMEANEYYPARYQSHMLIGHGFASPGDCGGILRCQHGVIGIITAGGEGLVAFSDIRDLYAYEEEAMEQ
350. GFGHQNKAVYTAGYKICNYHLATQEDLQNAVSVMWNRDLLVTESRALGTDSIARCSCNTGVYYCESRRKYYPVSFIGPTFQYMEANEYYPARYQSHMLIGHGFASPGDCGGILRCQHGVIGIITAGGEGLVAFSDIRDLYAYEEEAMEQ
351. GFGHQNKAVYTAGYKICNYHLATQEDLQNAVSVMWNRDLLVAESRALGSDSIARCSCNTGVYYCESRRKYYPVSFIGPTFQYMEANEYYPARYQSHMLIGHGFASPGDCGGILRCQHGVIGIITAGGEGLVAFSDIRDLYAYEEEAMEQ
352. GFGHQNKAVYTAGYKICNYHLATQEDLQSAVNIMWDRDLLVAESRAQGTDSIARCTCKTGVYYCESKRRYYPVSFIGPTFQYMEANEYYPARYQSHMLIGHGFASPGDCGGILRCQHGVIGIITAGGEGLVAFSDIRDLYAYEEEAMEQ
353. GFGHQNKAVYTAGYKICNYHLATQEDLQNAVSVMWNRDLLVAESRALGTDSIARCSCNTGVYYCESKRKYYPVTFIGPTFQYMEANDYYPARYQSHMLIGHGFASPGDCGGILRCQHGVIGIITAGGEGLVAFSDIRDLYAYEEEAMEQ
354. GFGHQNKAVYTAGYKICNYHLATQEDLQNAVSVMWNRDLLVAESRALGTDSIARCSCNTGVYYCESRRKYYPVSFIGPTFQYMEANEYYPARYQSHMLIGHGFASPGDCGGILRCQHGVIGIITAGGEGLVAFSDIRDLYAYEEEAMEQ
355. GFGHQNKAVYTAGYKICNYHLATQEDLQNAVSVMWNRDLLVAESRTLGTDSIARCSCNTGVYYCESRRKYYPVSFIGPTFQYMEANEYYPARYQSHMLIGHGFASPGDCGGILRCQHGVIGIITAGGEGLVAFSDIRDLYAYEEEAMEQ
356. GFGHQNKAVYTAGYKICNYHLATQEDLQNAVSVMWNRDLLVAESRALGTDSIARCSCNTGVYYCESRRKYYPVSFIGPTFQYMEANEYYPARYQSHMLIGHGFASPGDCGGILRCQHGVIGIITAGGEGLVAFSDIRDLYAYEEEAMEQ
357. GFGHQNKAVYTAGYKICNYHLATPEDLQNAVSVMWNRDLLVAESRALGTDSIARCSCNTGVYYCESRRKYYPVSFIGPTFQYMEANEYYPARYQSHMLIGHGFASPGDCGGILRCQHGVIGIITAGGEGLVAFSDIRDLYAYEEEAMEQ
358. GFGHQNKAVYTAGYKICNYHLATQEDLQNAVSVMWNRDLLVVESRAQGTDSIARCNCNTGIYYCESRRKYYPVTFIGPTFQYMEANEYYPARYQSHMLIGHGFASPGDCGGILRCQHGVIGIVTAGGEGLVAFSDIRDLYAYEEEAMEQ
359. GFGHQNKAVYTAGYKICNYHLATQEDLQNAVSVMWNRDLLVAESRALGTDSIARCSCNTGVYYCESRRKYYPVSFIGPTFQYMEANEYYPARYQSHMLIGHGFASPGDCGGILRCQHGVIGIITAGGEGLVAFSDIRDLYAYEEEAMEQ
360. GFGHQNKAVYTAGYKICNYHLATQEDLQNAVAVMWNRDLLVTESKAQGTDLIARCNCNTGVYYCESRRKYYPVSFIGPTFQYMEANDYYPARYQSHMLIGHGFASPGDCGGILRCQHGVIGIITAGGEGLVAFSDIRDLYAYEEEAMEQ
361. GFGHQNKAVYTAGYKICNYHLATQEDLQNAVSVMWNRDLLVAESRALGTDSIARCSCNTGVYYCESRRKYYPVSFIGPTFQYMEANEYYPARYQSHMLIGHGFASPGDCGGILRCQHGVIGIITAGGEGLVAFSDIRDLYAYEEEAMEQ
362. GFGHQNKAVYTAGYKICNYHLATQEDLQNAVSVMWNRDLLVAESRALGTDSIARCSCNTGVYYCESRRKYYPVSFIGPTFQYMEANEYYPARYQSHMLIGHGFASPGDCGGILRCQHGVIGIITAGGEGLVAFSDIRDLYAYEEEAMEQ
363. GFGHQNKAVYTAGYKICNYHLATQEDLQNAVSVMWNRDLLVAESRALGTDSIARCSCNTGVYYCESRRKYYPVSFIGPTFQYMEANEYYPARYQSHMLIGHGFASPGDCGGILRCQHGVIGIITAGGEGLVAFSDIRDLYAYEEEAMEQ
364. GFGHQNKAVYTAGYKICNYHLATQEDLQNAVSVMWNRDLLVAESRALGTDSIARCSCNTGVYYCESRRKYYPVSFIGPTFQYMEANEYYPARYQSHMLIGHGFASPGDCGGILRCQHGVIGIITAGGEGLVAFSDIRDLYAYEEEAMEQ
365. GFGHQNKAVYTAGYKICNYHLATQEDLQNAVSVMWDRDLLVVESKASGIDSIARCSCNAGVYYCESRGKHYPVSFIGPTFQYMEANEYYPARYQSHMLIGHGFASPGDCGGILRCQHGVIGLVTAGGEGLVAFSDIRDLYAYEEEAMEQ
366. GFGHQNKAVYTAGYKICNYHLATQEDLQNAVSVMWNRDLLVAESRALGTDSIARCSCNTGVYYCESRRKYYPVSFIGPTFQYMEANEYYPARYQSHMLIGHGFASPGDCGGILRCQHGVIGIITAGGEGLVAFSDIRDLYAYEEEAMEQ
367. GFGHQNKAVYTAGYKICNYHLATQEDLQNAVSVMWNRDLLVAESRALGTDSIARCSCNTGVYYCESRRKYYPVSFIGPTFQYMEANEYYPARYQSHMLIGHGFASPGDCGGILRCQHGVIGIITAGGEGLVAFSDIRDLYAYEEEAMEQ
368. GFGHQNKAVYTAGYKICNYHLATQEDLQNAVSVMWNRDLLVAESRALGTDSIARCSCNTGVYYCESRRKYYPVSFIGPTFQYMEANEYYPARYQSHMLIGHGFASPGDCGGILRCQHGVIGIITAGGEGLVAFSDIRDLYAYEEEAMEQ
369. GFGHQNKAVYTAGYKICNYHLATQEDLQNAVSVMWNRDLLVAESRALGTDSIARCSCNTGVYYCESRRKYYPVSFIGPTFQYMEANEYYPARYQSHMLIGHGFASPGDCGGILRCQHGVIGIITAGGEGLVAFSDIRDLYAYEEEAMEQ
370. GFGHQNKAVYTAGYKICNYHLATQEDLQNAVSVMWNRDLLVAESRALGTDSIARCSCNTGVYYCESRRKYYPVSFIGPTFQYMEANEYYPARYQSHMLIGHGFASPGDCGGILRCQHGVIGIITAGGEGLVAFSDIRDLYAYEEEAMEQ
371. GFGHQNKAVYTAGYKICNYHLATLEDLQNAVSVMWNRDLLVAESRALGTDSIARCSCNTGVYYCESRRKYYPVSFIGPTFQYMEANEYYPARYQSHMLIGHGFASPGDCGGILRCQHGVIGIITAGGEGLVAFSDIRDLYAYEEEAMEQ
372. GFGHQNKAVYTAGYKICNYHLATQEDLQNAVSVMWNRDLLVAESRALGTDSIARCSCNTGVYYCESRRKYYPVSFIGPTFQYMEANEYYPARYQSHMLIGHGFASPGDCGGILRCQHGVIGIITAGGEGLVAFSDIRDLYAYEEEAMEQ
373. GFGHQNKAVYTAGYKICNYHLATQEDLQNAVSVMWNRDLLVAESRALGTDSIARCSCNTGVYYCESRRKYYPVSFIGPTFQYMEANEYYPARYQSHMLIGHGFASPGDCGGILRCQHGVIGIITAGGEGLVAFSDIRDLYAYEEEAMEQ
374. GFGHQNKAVYTAGYKICNYHLATQEDLQNAVSVMWNRDLLVAESRALGTDSIARCSCNTGVYYCESRRKHYPVSFIGPTFQYMEANEYYPARYQSHMLIGHGFASPGDCGGILRCQHGVIGIITAGGEGLVAFSDIRDLYAYEEEAMEQ
375. GFGHQNKAVYTAGYKICNYHLATQEDLQNAVSVMWNRDLLVVESRALGTDSIARCNCNTGVYYCESRRKYYPVSFIGPTFQYMEANEYYPARYQSHMLIGHGFASPGDCGGILRCQHGVIGIITAGGEGLVAFSDIRDLYAYEEEAMEQ
376. GFGHQNKAVYTAGYKICNYHLATQEDLQNAVSVMWNRDLLVAESRALGTDSIARCNCNTGVYYCESRRKYYPVSFIGPTFQYMEANEYYPARYQSHMLIGHGFASPGDCGGILRCQHGVIGIITAGGEGLVAFSDIRDLYAYEEEAMEQ
377. GFGHQNKAVYTAGYKICNYHLATQEDLQNAVSVMWNRDLLVAESRALGTDSIARCNCNTGVYYCESRRKYYPVSFIGPTFQYMEANEYYPARYQSHMLIGHGFASPGDCGGILRCQHGVIGIITAGGEGLVAFSDIRDLYAYEEEAMEQ
378. GFGHQNKAVYTAGYKICNYHLATQEDLQNAVSVMWNRDLLVVESRALGTDSIARCNCNTGVYYCESRRKYYPVSFIGPTFQYMEANEYYPARYQSHMLIGHGFASPGDCGGILRCQHGVIGIITAGGEGLVAFSDIRDLYAYEEEAMEQ
379. GFGHQNKAVYTAGYKICNYHLATQEDLQNAVSVMWNRDLLVVESRALGTDSIARCNCNTGVYYCESRRKYYPVSFIGPTFQYMEANEYYPARYQSHMLIGHGFASPGDCGGILRCQHGVIGIITAGGEGLVAFSDIRDLYAYEEEAMEQ
380. GFGHQNKAVYTAGYKICNYHLATQEDLQNAVSVMWNRDLLVVESRALGTDSIARCNCNTGVYYCESRRKYYPVSFIGPTFQYMEANEYYPARYQSHMLIGHGFASPGDCGGILRCQHGVIGIITAGGEGLVAFSDIRDLYAYEEEAMEQ
381. GFGHQNKAVYTAGYKICNYHLATQEDLQNAVSVMWNRDLLVAESRALGTDSIARCNCNTGVYYCESRRKYYPVSFIGPTFQYMEANEYYPARYQSHMLIGHGFASPGDCGGILRCQHGVIGIITAGGEGLVAFSDIRDLYAYEEEAMEQ
382. GFGHQNKAVYTAGYKICNYHLATQEDLQNAVSVMWNRDLLVAESRALGTDSIARCSCNTGVYYCESRRKYYPVSFIGPTFQYMEANEYYPARYQSHMLIGHGFASPGDCGGILRCQHGVIGIITAGGEGLVAFSDIRDLYAYEEEAMEQ
383. GFGHQNKAVYTAGYKICNYHLATQEDLQNAVSVMWNRDLLVAESRALGTDSIARCNCNTGVYYCESRRKYYPVSFIGPTFQYMEANEYYPARYQSHMLIGHGFASPGDCGGILRCQHGVIGIITAGGEGLVAFSDIRDLYAYEEEAMEQ
384. GFGHQNKAVYTAGYKICNYHLATQEDLQNAVNVMWNRDLLVAESRALGTDSIARCSCNTGVYYCESRRKYYPVSFIGPTFQYMEANEYYPARYQSHMLIGHGFASPGDCGGILRCQHGVIGIITAGGEGLVAFSDIRDLYAYEEEAMEQ
385. GFGHQNKAVYTAGYKICNYHLATQEDLQNAVSVMWNRDLLVVESRALGTDSIARCNCNTGVYYCESRRKYYPVSFIGPTFQYMEANEYYPARYQSHMLIGHGFASPGDCGGILRCQHGVIGIITAGGEGLVAFSDIRDLYVYEEEAMEQ
386. GFGHQNKAVYTAGYKICNYHLATQEDLQNAVSVMWNRDLLVAESRALGTDSIARCSCNTGVYYCESRRKYYPVSFIGPTFQYMEANEYYPARYQSHMLIGHGFASPGDCGGILRCQHGVIGIITAGGEGLVAFSDIRDLYAYEEEAMEQ
387. GFGHQNKAVYTAGYKICNYHLATQEDLQNAVSVMWNRDLLVVESRALGTDSIARCNCNTGVYYCESRRKYYPVSFIGPTFQYMEANEYYPARYQSHMLIGHGFASPGDCGGILRCQHGVIGIITAGGEGLVAFSDIRDLYAYEEEAMEQ
388. GFGHQNKAVYTAGYKICNYHLATQEDLQNAVSVMWNRDLLVVESRALGTDSIARCNCNTGVYYCESRRKYYPVSFIGPTFQYMEANEYYPARYQSHMLIGHGFASPGDCGGILRCQHGVIGIITAGGEGLVAFSDIRDLYAYEEEAMEQ
389. GFGHQNKAVYTAGYKICNYHLATQEDLQNAVSVMWNRDLLVVESRALGTDSIARCNCNTGVYYCESRRKYYPVSFIGPTFQYMEANEYYPARYQSHMLIGHGFASPGDCGGILRCQHGVIGIITAGGEGLVAFSDIRDLYAYEEEAMEQ
390. GFGHQNKAVYTAGYKICNYHLATQEDLQNAVSVMWNRDLLVAESRALGTDSIARCSCNTGVYYCESRRKYYPVSFIGPTFQYMEANEYYPARYQSHMLIGHGFASPGDCGGILRCQHGVIGIITAGGEGLVAFSDIRDLYAYEEEAMEQ
391. GFGHQNKAVYTAGYKICNYHLATQEDLQNAVSVMWNRDLLVAESRALGTDSIARCSCNTGVYYCESRRKYYPVSFIGPTFQYMEANEYYPARYQSHMLIGHGFASPGDCGGILRCQHGVIGIITAGGEGLVAFSDIRDLYAYEEEAMEQ
392. GFGHQNKAVYTAGYKICNYHLATQEDLQNAVNVMWNRDLLVAESRALGTDSIARCNCNTGVYYCESRRKYYPVSFIGPTFQYMEANEYYPARYQSHMLIGHGFASPGDCGGILRCQHGVIGIITAGGEGLVAFSDIRDLYAYEEEAMEQ
393. GFGHQNKAVYTAGYKICNYHLATQEDLQNAVSVMWNRDLLVVESRAQGTDSIARCSCNTGVYYCESRRKYYPVSFIGPTFQYMEANEYYPARYQSHMLIGHGFASPGDCGGILRCQHGVIGIITAGGEGLVAFSDIRDLYAYEEEAMEQ
394. GFGHQNKAVYTAGYKICNYHLATQEDLQNAVSVMWNRDLLVAESRALGTDSIARCSCNTGVYYCESRRKYYPVSFIGPTFQYMEANEYYPARYQSHMLIGHGFASPGDCGGILRCQHGVIGIITAGGEGLVAFSDIRDLYAYEEEAMEQ
395. GFGHQNKAVYTAGYKICNYHLATQEDLQNAVSVMWNRDLLVAESRALGTDSIARCSCNTGVYYCESRRKYYPVSFIGPTFQYMEANEYYPARYQSHMLIGHGFASPGDCGGILRCQHGVIGIITAGGEGLVAFSDIRDLYAYEEEAMEQ
396. GFGHQNKAVYTAGYKICNYHLATQEDLQNAVSVMWNRDLLVAESRALGTDSIARCSCNTGVYYCESRRKYYPVSFIGPTFQYMEANEYYPARYQSHMLIGHGFASPGDCGGILRCQHGVIGIITAGGEGLVAFSDIRDLYAYEEEAMEQ
397. GFGHQNKAVYTAGYKICNYHLATQEDLQNAVSVMWNRDLLVAESKAQGTDSIARCNCNTGVYYCESRRKYYPVSFIGPTFQYMEANDYYPARYQSHMLIGHGFASPGDCGGILRCQHGVIGIITAGGEGLVAFSDIRDLYAYEEEAMEQ
398. GFGHQNKAVYTAGYKICNYHLATQEDLQHAVNIMWNRDLLVAESKAQGTDSIARCNCNTGVYYCESRKKYYPVSFIGPTFQYMEANDYYPARYQSHMLIGHGFASPGDCGGILRCQHGVIGIITAGGEGLVAFSDIRDLHAYEEEAMEQ
399. GFGHQNKAVYTAGYKICNYHLATQEDLQNAVSVMWNRDLLVAESRALGTDSIARCSCNTGVYYCESRRKYYPVSFIGPTFQYMEANEYYPARYQSHMLIGHGFASPGDCGGILRCQHGVIGIITAGGEGLVAFSDIRDLYAYEEEAMEQ
400. GFGHQNKAVYTAGYKICNYHLATKEDLQNAVSIMWNRDLLVVESKAQGTDSIARCNCNAGVYYCESRRKYYPVSFVGPTFQYMEANDYYPARYQSHMLIGHGFASPGDCGGILRCQHGVIGIVTAGGEGLVAFSDIRDLYAYEEEAMEQ
401. GFGHQNKAVYTAGYKICNYHLATQEDLQNAVSVMWNRDLLVAESRALGTDSIARCSCNTGVYYCESRRKYYPVSFIGPTFQYMEANEYYPARYQSHMLIGHGFASPGDCGGILRCQHGVIGIITAGGEGLVAFSDIRDLYAYEEEAMEQ
402. GFGHQNKAVYTAGYKICNYHLATQEDLQNAVNIMWIRDLLVVESKAQGIDSIARCNCHTGVYYCESRRKYYPVSFTGPTFQYMEANEYYPARYQSHMLIGYGFASPGDCGGILRCQHGVIGIITAGGEGLVAFSDIRDLYAYEEEAMEQ
403. GFGHQNKAVYTAGYKICNYHLATQEDLRNAVSVMWNRDLLVTESRALGTDSIARCSCNTGVYYCESRRKYYPVSFIGPTFQYMEANEYYPARYQSHMLIGHGFASPGDCGGILRCQHGVIGIITAGGEGLVAFSDIRDLYAYEEEAMEQ
404. GFGHQNKAVYTAGYKICNYHLATQEDLQNAVSIMWNRDLLVAESRALGTDSIARCSCNTGLYYCESRRKYYPVSFIGPTFQYMEANEYYPARYQSHMLIGHGFASPGDCGGILRCQHGVIGIVTAGGEGLVAFSDIRDLYAYEEEAMEQ
405. GFGHQNKAVYTAGYKICNYHLATQEDLQNAVSIMWNRDLLVAESRALGTDSIARCSCNTGVYYCESRRKYYPVSFIGPTFQYMEANEYYPARYQSHMLIGHGFASPGDCGGILRCQHGVIGIVTAGGEGLVAFSDIRDLYAYEEEAMEQ
406. GFGHQNKAVYTAGYKICNYHLATQEDLQNAVSIMWNRDLLVAESRALGTDSIARCSCNTGVYYCESRRKYYPVSFIGPTFQYMEANEYYPARYQSHMLIGHGFASPGDCGGILRCQHGVIGIVTAGGEGLVAFSDIRDLYAYEEEAMEQ
407. GFGHQNKAVYTAGYKICNYHLATQEDLQNAVSIMWNRDLLVAESRALGTDSIARCSCNTGVYYCESRRKYYPVSFIGPTFQYMEANEYYPARYQSHMLIGHGFASPGDCGGILRCQHGVIGIVTAGGEGLVAFSDIRDLYAYEEEAMEQ
408. GFGHQNKAVYTAGYKICNYHLATQEDLQNAVSIMWDRDLLVVESRAPGTDSIARCSCNAGVYYCESRRKYYPVSFIGPTFQFMEANDYYPARYQSHMLIGHGFASPGDCGGILRCQHGVIGIVTAGGEGLVAFSDIRDLHAYEEEAMEQ
409. GFGHQNKAVYTAGYKICNYHLATQEDLQNAVSVMWDRDLLVVESRAPGTDSIARCSCNAGVYYCESRRKYYPVSFIGPTFQFMEANDYYPARYQSHMLIGHGFASPGDCGGILRCQHGVIGIITAGGEGLVAFSDIRDLHAYEEEAMEQ
410. GFGHQNKAVYTAGYKICNYHLATQEDLQNAVSIMWDRDLLVVESRAPGTDSIARCSCNAGVYYCESRRKYYPVSFIGPTFQFMEANDYYPARYQSHMLIGHGFASPGDCGGILRCQHGVIGIVTAGGEGLVAFSDIRDLHAYEEEAMEQ
411. GFGHQNKAVYTAGYKICNYHLATQEDLQNAVSVMWDRDLLVVESKAPGTDSIARCSCNAGVYYCESRRKYYPVSFVGPTFQFMEANDYYPARYQSHMLIGHGFASPGDCGGILRCQHGVIGIITAGGEGLVAFSDIRDLHAYEEEAMEQ
412. GFGHQNKAVYTAGYKICNYHLATQEDLQNAVSVMWDRDLLVVESRAPGTDSIARCSCNAGVYYCESRRKYYPVSFIGPTFQFMEANDYYPARYQSHMLIGHGFASPGDCGGILRCQHGVIGIITAGGEGLVAFSDIRDLHAYEEEAMEQ
413. GFGHQNKAVYTAGYKICNYHLATQEDFQNAVCVMWNRDHLVTESRASGTDSIARCRCNTGVYYCESRRKYYPVSFVGPTFQYMEANEYYPARYQSHMLIGHGFASPGDCGGILRCQHGVIGIITAGGEGLVAFSDIRDLYAYEEEAMEQ
414. GFGHQNKAVYTAGYKICNYHLATQEDLQNAVSVMWNRDLLVAESRALGTDSIARCSCNTGVYYCESRRKYYPVSFIGPTFQYMEANEYYPARYQSHMLIGHGFASPGDCGGILRCQHGVIGIITAGGEGLVAFSDIRDLYAYEEEAMEQ
415. GFGHQNKAVYTAGYKICNYHLATQEDMQNAVSIMWNRDLLIAESKAQGTDTIARCSCRTGVYYCESRRKYYPVSFVGPTFQYMEANDYYPARYQSHMLIGHGFASPGDCGGILRCQHGVIGIVTAGGGGLVAFSDIRDLYAYEEEAMEQ
416. GFGHQNKAVYTAGYKICNYHLATQEDLQNAVSVMWNRDLLVAESRALGTDSIARCSCNTGVYYCESRRKYYPVSFIGPTFQYMEANEYYPARYQSHMLIGHGFASPGDCGGILRCQHGVIGIITAGGEGLVAFSDIRDLYAYEEEAMEQ
417. GFGHQNKAVYTAGYKICNYHLATQEDLQNAVSVMWNRDLLVAESRALGTDSIARCSCNTGVYYCESRRKYYPVSFIGPTFQYMEANEYYPARYQSHMLIGHGFASPGDCGGILRCQHGVIGIITAGGEGLVAFSDIRDLYAYEEEAMEQ
418. GFGHQNKAVYTAGYKICNYHLATQEDLQNAVNIMWIRDLLVVESKAQGIDSIARCNCHTGVYYCESRRKYYPVSFTGPTFQYMEANEYYPARYQSHMLIGHGFASPGDCGGILRCQHGVIGIITAGGEGLVAFSDIRDLYAYEEEAMEQ
419. GFGHQNKAVYTAGYKICNYHLATQEDLQNAVSVMWNRDLLVAESRALGTDSIARCSCNTGVYYCESRRKYYPVSFIGPTFQYMEANEYYPARYQSHMLIGHGFASPGDCGGILRCQHGVIGIITAGGEGLVAFSDIRDLYAYEEEAMEQ
420. GFGHQNKAVYTAGYKICNYHLATQEDLQNAINIMWIRDLLVVESKAQGIDSIARCNCHTGVYYCESRRKYYPVSFVGPTFQYMEANEYYPARYQSHMLIGHGFASPGDCGGILRCQHGVIGIITAGGEGLVAFSDIRDLYAYEVEAMEQ
421. GFGHQNKAVYTAGYKICNYHLATQEDLQNAINIMWIRDLLVVESKAQGIDSIARCNCHTGVYYCESRRKYYPVSFVGPTFQYMEANEYYPARYQSHMLIGHGFASPGDCGGILRCQHGVIGIITAGGEGLVAFSDIRDLYAYEVEAMEQ
422. GFGHQNKAVYTAGYKICNYHLATQEDLQNAVNIMWIRDLLVVESKAQGIDSIARCNCHTGVYYCESRRKYYPVSFTGPTFQYMEANEYYPARYQSHMLIGHGFASPGDCGGILRCQHGVIGIITAGGEGLVAFSDIRDLYAYEEEAMEQ
423. GFGHQSKAVYTAGYKICNYHLATKEDLQNAVSIMWNRDLLVVESKAQGTDSIARCNCNAGVYYCESRRKYYPVSFVGPTFQYMEANDYYPARYQSHMLIGHGFASPGDCGGILRCQHGVIGIVTAGGEGLVAFSDIRDLYAYEEEAMEQ
424. GFGHQNKAVYTTGYKICNYHLATVEDLQNAVNIMWNRDLLVAKSKAQGTDSIARCNCKAGVYYCESRRKYYPVSFVGPTFQYMEANDYYPARYQSHMLIGCGFASPGDCGGILRCQHGVIGIVTAGGEGLVAFSDIRDLHAYEEEAMEQ
425. GFGHQNKAVYTAGYKICNYHLATKEDLQNAVSIMWNRDLLVVESKAQGTDSIARCNCNAGVYYCESRRKYYPVSFVGPTFQYMEANDYYPARYQSHMLIGHGYASPGDCGGILRCQHGVIGIVTAGGEGLVAFSDIRDLYAYEEEAMEQ
426. GFGHQNKAVYTAGYKICNYHLATKEDLQNAVSIMWNRDLLIVESKAQGTDSIARCNCNAGVYYCESRRKYYPVSFVGPTFQYMEANDYYPARYQSHMLIGHGFASPGDCGGILRCQHGVIGIVTAGGEGLVAFSDIRDLYAYEEEAMEQ
427. GFGHQNKAVYTAGYKICNYHLATKEDLQNAVSIMWNRDLLVVESKAQGTDSIARCNCNAGVYYCESRRKYYPVSFVGPTFQYMEANDYYPARYQSHMLIGHGFASPGDCGGILRCQHGVIGIVTAGGEGLVAFSDIRDLYAYEEEAMEQ
428. GFGHQNKAVYTAGYKICNYHLATKEDLQNAVSIMWNRDLLVVESKAQGTDSIARCNCNAGVYYCESRRKYYPVSFVGPTFQYMEANDYYPARYQSHMLIGHGFASPGDCGGILRCQHGVIGIVTAGGEGLVAFSDMRDLYAYEEEAMEQ
429. GFGHQNKAVYTAGYKICNYHLATKEDLQNAVSIMWNRDLLVVESKAQGTDSIARCNCNAGVYYCESRRKYYPVSFVGPTFQYMEANDYYPARYQSHMLIGHGFASPGDCGGILRCQHGVIGIVTAGGEGLVAFSDIRDLYAYEEEAMEQ
430. GFGHQNKAVYTAGYKICNYHLATKEDLQNAVSIMWNRDLLVVESKAQGTDSIARCNCNAGVYYCESRRKYYPVSFVGPTFQYMEANDYYPARYQSHMLIGHGFASPGDCGGILRCQHGVIGIVTAGGEGLVAFSDIRDLYAYEEEAMEQ
431. GFGHQNKAVYTAGYKICNYHLATKEDLQNAVSIMWNRDLLVVESKAQGTDSIARCNCNAGVYYCESRRKYYPVSFVGPTFQYMEANDYYPARYQSHMLIGHGFASPGDCGGILRCQHGVIGIVTAGGEGLVAFSDIRDLYAYEEEAMEQ
432. GFGHQNKAVYTAGYKICNYHLATKEDLQNAVSIMWDRDLLVVESKAQGTDSIARCNCNAGVYYCESRRKYYPVSFVGPTFQYMEANDYYPARYQSHMLIGHGFASPGDCGGILRCQHGVIGIVTAGGEGLVAFSDIRDLYAYEEEAMEQ
433. GFGHQNKAVYTAGYKICNYHLATKEDLQNAVSIMWNRDLLVVESKAQGTDSIARCNCNAGVYYCESRRKYYSVSFVGPTFQYMEANDYYPARYQSHMLIGHGFASPGDCGGILRCQHGVIGIVTAGGEGLVAFSDIRDLYAYEEEAMEQ
434. GFGHQNKAVYTAGYKICNYHLATKEDLQNAVSIMWNRDLLVVESKAQGTDSIARCNCNAGVYYCESRRKYYPVSFVGPTFQYMEANDYYPARYQSHMLIGHGFASPGDCGGILRCQHGVIGIVTAGGEGLVAFSDIRDLYAYEEEAMEQ
435. GFGHQNKAVYTAGYKICNYHLATKEDLQNAVSIMWNRDLLVVESKAQGTDSIARCNCNAGVYYCESRRKYYPVSFVGPTFQYMEANDYYPARYQSHMLIGHGFASPGDCGGILRCQHGVIGIVTAGGEGLVAFSDIRDLYAYEEEAMEQ
436. GFGHQNKAVYTAGYKICNYHLATKEDLQNAVSIMWNRDLLVVESKAQGTDSIARCNCNAGVYYCESRRKYYPVSFVGPTFQYMEANDYYPARYQSHMLIGHGFASPGDCGGILRCQHGVIGIVTAGGEGLVAFSDIRDLYAYEEEAMEQ
437. GFGHQNKAVYTAGYKICNYHLATKEDLQNAVSIMWNRDLLVVESKAQGTDSIARCNCNAGVYYCESRRKYYPVSFVGPTFQYMEANDYYPARYQSHMLIGHGFASPGDCGGILRCQHGVIGIVTAGGEGLVAFSDIRDLYAYEEEAMEQ
438. GFGHQNKAVYTAGYKICNYHLATKEDLQNAVSIMWNRDLLVVESKAQGTDSIARCNCNAGVYYCESRRKYYPVSFVGPTFQYMEANDYYPARYQSHMLIGHGFASPGDCGGILRCQHGVIGIVTAGGEGLVAFSDIRDLYAYEEEAMEQ
439. GFGHQNKAVYTAGYKICNYHLATKEDLQNAVSIMWNRDLLVVESKAQGTDSIARCNCNAGVYYCESRRKYYPVSFVGPTFQYMEANDYYPARYQSHMLIGHGFASPGDCGGILRCQHGVIGIVTAGGEGLVAFSDIRDLYAYEEEAMEQ
440. GFGHQNKAVYTAGYKICNYHLATKEDLQNAVSIMWNRDLLVVESKAQGTDSIARCNCNAGVYYCESRRKYYPVSFVGPTFQYMEANDYYPARYQSHMLIGHGFASPGDCGGILRCQHGVIGIVTAGGEGLVAFSDIRDLYAYEEEAMEQ
441. GFGHQNKAVYTAGYKICNYHLATKEDLQNAVSIMWNRDLLVVESKAQGTDSIARCNCNAGVYYCESRRKYYPVSFVGPTFQYMEANDYYPARYQSHMLIGHGFASPGDCGGILRCQHGVIGIVTAGGEGLVAFSDIRDLYAYEEEAMEQ
442. GFGHQNKAVYTAGYKVCNYHLATKEDLQNAVSIMWNRDLLVVESKAQGTDSIARCNCNAGVYYCESRRKYYPVSFVGPTFQYMEANDYYPARYQSHMLIGHGFASPGDCGGILRCQHGVIGIVTAGGEGLVAFSDIRDLYAYEEEAMEQ
443. GFGHQNKAVYTAGYKICNYHLATKEDLQNAVSIMWNRDLLVVESKAQGTDSIARCNCNAGVYYCESRRKYYPVSFVGPTFQYMEANDYYPARYQSHMLIGHGFASPGDCGGILRCQHGVIGIVTAGGEGLVAFSDIRDLYAYEEEAMEQ
444. GFGHQNKAVYTAGYKICNYHLATKEDLQNAVSIMWNRDLLVVESKAQGTDSIARCNCNAGVYYCESRRKYYPVSFVGPTFQYMEANDYYPARYQSHMLIGHGFASPGDCGGILRCQHGVIGIVTAGGEGLVAFSDIRDLYAYEEEAMEQ
445. GFGHQNKAVYTAGYKICNYHLATKEDLQNAVSIMWNRDLLVVESKAQGTDSIARCNCNAGVYYCESRRKYYPVSFVGPTFQYMEANDYYPARYQSHMLIGHGFASPGDCGGILRCQHGVIGIVTAGGEGLVAFSDIRDLYAYEEEAMEQ
446. GFGHQNKAVYTAGYKICNYHLATKEDLQNAVSIMWNRDLLVVESKAQGTDSIARCNCNAGVYYCESRRKYYPVSFVGPTFQYMEANDYYPARYQSHMLIGHGFASPGDCGGILRCQHGVIGIVTAGGEGLVAFSDIRDLYAYEEEAMEQ
447. GFGHQNKAVYTAGYKICNYHLATKEDLQNAVSIMWNRDLLVVESKAQGTDSIARCNCNAGVYYCESRRKYYPVSFVGPTFQYMEANDYYPARYQSHMLIGHGFASPGDCGGILRCQHGVIGIVTAGGEGLVAFSDIRDLYAYEEEAMEQ
448. GFGHQNKAVYTAGYKICNYHLATKEDLQNAVSIMWNRDLLVVESKAQGTDSIARCNCNAGVYYCESRRKYYPVSFVGPTFQYMEANDYYPARYQSHMLIGHGFASPGDCGGILRCQHGVIGIVTAGGEGLVAFSDIRDLYAYEEEAMEQ
449. GFGHQNKAVYTAGYKICNYHLATKEDLQNAVSIMWNRDLLVVESKAQGTDSIARCNCNAGVYYCESRRKYYPVSFVGPTFQYMEANDYYPARYQSHMLIGHGFASPGDCGGILRCQHGVIGIVTAGGEGLVAFSDIRDLYAYEEEAMEQ
450. GFGHQNKAVYTAGYKICNYHLATKEDLQNAVSIMWNRDLLVVESKAQGTDSIARCNCNAGVYYCESRRKYYPVSFVGPTFQYMEANDYYPARYQSHMLIGHGFASPGDCGGILRCQHGVIGIVTAGGEGLVAFSDIRDLYAYEEEAMEQ
451. GFGHQNKAVYTAGYKICNYHLATKEDLQNAVSIMWNRDLLVVESKAQGTDSIARCNCNAGVYYCESRRKYYPVSFVGPTFQYMEANDYYPARYQSHMLIGHGFASPGDCGGILRCQHGVIGIVTAGGEGLVAFSDIRDLYAYEEEAMEQ
452. GFGHQNKAVYTAGYKICNYHLATKEDLQNAVSIMWNRDLLVVESKAQGTDSIARCNCNAGVYYCESRRKYYPVSFVGPTFQYMEANDYYPARYQSHMLIGHGFASPGDCGGILRCQHGVIGIVTAGGEGLVAFSDIRDLYAYEEEAMEQ
453. GFGHQNKAVYTAGYKICNYHLATKEDLQNAVSIMWNRDLLVVESKAQGTDSIARCNCNAGVYYCESRRKYYPVSFVGPTFQYMEANDYYPARYQSHMLIGHGFASPGDCGGILRCQHGVIGIVTAGGEGLVAFSDIRDLYAYEEEAMEQ
454. GFGHQNKAVYTAGYKICNYHLATKEDLQNAVSIMWNRDLLVVESKAQGTDSIARCNCNAGVYYCESRRKYYPVSFVGPTFQYMEANDYYPARYQSHMLIGHGFASPGDCGGILRCQHGVIGIVTAGGEGLVAFSDIRDLYAYEEEAMEQ
455. GFGHQNKAVYTAGYKICNYHLATKEDLQNAVSIMWNRDLLVVESKAQGTDSIARCNCNAGVYYCESRRKYYPVSFVGPTFQYMEANDYYPARYQSHMLIGHGFASPGDCGGILRCQHGVIGIVTAGGEGLVAFSDIRDLYAYEEEAMEQ
456. GFGHQNKAVYTAGYKICNYHLATKEDLQNAVSIMWNRDLLVVESKAQGTDSIARCNCNAGVYYCESRRKYYPVSFVGPTFQYMEANDYYPARYQSHMLIGHGFASPGDCGGILRCQHGVIGIVTAGGEGLVAFSDIRDLYAYEEEAMEQ
457. GFGHQNKAVYTAGYKICNYHLATKEDLQNAVSIMWNRDLLVVESKAQGTDSIARCNCNAGVYYCESRRKYYPVSFVGPTFQYMEANDYYPARYQSHMLIGHGFASPGDCGGILRCQHGVIGIVTAGGEGLVAFSDIRDLYAYEEEAMEQ
458. GFGHQNKAVYTAGYKICNYHLATKEDLQNAVSIMWNRDLLVVESKAQGTDSIARCNCNAGVYYCESRRKYYPVSFVGPTFQYMEANDYYPARYQSHMLIGHGFASPGDCGGILRCQHGVIGIVTAGGEGLVAFSDIRDLYAYEEEAMEQ
459. GFGHQNKAVYTAGYKICNYHLATKEDLQNAVSIMWNRDLLVVESKAQGTDSIARCNCNAGVYYCESRRKYYPVSFVGPTFQYMEANDYYPARYQSHMLIGHGFASPGDCGGILRCQHGVIGIVTAGGEGLVAFSDIRDLYAYEEEAMEQ
460. GFGHQNKAVYTAGYKICNYHLATKEDLQNAVSIMWNRDLLVVESKAQGTDSIARCNCNAGVYYCESRRKYYPVSFVGPTFQYMEANDYYPARYQSHMLIGHGFASPGDCGGILRCQHGVIGIVTAGGEGLVAFSDIRDLYAYEEEAMEQ
461. GFGHQNKAVYTAGYKICNYHLATKEDLQNAVSIMWNRDLLVVESKAQGTDSIARCNCNAGVYYCESRRKYYPVSFVGPTFQYMEANDYYPARYQSHMLIGHGFASPGDCGGILRCQHGVIGIVTAGGEGLVAFSDIRDLYAYEEEAMEQ
462. GFGHQNKAVYTAGYKICNYHLATKEDLQNAVSIMWNRDLLVVESKAQGTDSIARCNCNAGVYYCESRRKYYPVSFVGPTFQYMEANDYYPARYQSHMLIGHGFASPGDCGGILRCQHGVIGIVTAGGEGLVAFSDIRDLYAYEEEAMEQ
463. GFGHQNKAVYTAGYKICNYHLATKEDLQNAVSIMWNRDLLVVESKAQGTDSIARCNCNAGVYYCESRRKYYPVSFVGPTFQYMEANDYYPARYQSHMLIGHGFASPGDCGGILRCQHGVIGIVTAGGEGLVAFSDIRDLYAYEEEAMEQ
464. GFGHQNKAVYTAGYKICNYHLATKEDLQNAVSIMWNRDLLVVESKAQGTDSIARCNCNAGVYYCESRRKYYPVSFVGPTFQYMEANDYYPARYQSHMLIGHGFASPGDCGGILRCQHGVIGIVTAGGEGLVAFSDIRDLYAYEEEAMEQ
465. GFGHQNKAVYTAGYKICNYHLATKEDLQNAVSIMWNRDLLVVESKAQGTDSIARCNCNAGVYYCESRRKYYPVSFVGPTFQYMEANDYYPARYQSHMLIGHGFASPGDCGGILRCQHGVIGIVTAGGEGLVAFSDIRDLYAYEEEAMEQ
466. GFGHQNKAVYTAGYKICNYHLATKEDLQNAVSIMWNRDLLVVESKAQGTDSIARCNCNAGVYYCESRRKYYPVSFVGPTFQYMEANDYYPARYQSHMLIGHGFASPGDCGGILRCQHGVIGIVTAGGEGLVAFSDIRDLYAYEEEAMEQ
467. GFGHQNKAVYTAGYKICNYHLATKEDLQNAVSIMWNRDLLVVESKAQGTDSIARCNCNAGVYYCESRRKYYPVSFVGPTFQYMEANDYYPARYQSHMLIGHGFASPGDCGGILRCQHGVIGIVTAGGEGLVAFSDIRDLYAYEEEAMEQ
468. GFGHQNKAVYTAGYKICNYHLATKEDLQNAVSIMWNRDLLVVESKAQGTDSIARCNCNAGVYYCESRRKYYPVSFVGPTFQYMEANDYYPARYQSHMLIGHGFASPGDCGGILRCQHGVIGIVTAGGEGLVAFSDIRDLYAYEEEAMEQ
469. GFGHQNKAVYTAGYKICNYHLATKEDLQNAVSIMWNRDLLVVESKAQGTDSIARCNCNAGVYYCESRRKYYPVSFVGPTFQYMEANDYYPARYQSHMLIGHGFASPGDCGGILRCQHGVIGIVTAGGEGLVAFSDIRDLYAYEEEAMEQ
470. GFGHQNKAVYTAGYKICNYHLATKEDLQNAVSIMWNRDLLVVESKAQGTDSIARCNCNAGVYYCESRRKYYPVSFVGPTFQYMEANDYYPARYQSHMLIGHGFASPGDCGGILRCQHGVIGIVTAGGEGLVAFSDIRDLYAYEEEAMEQ
471. GFGHQNKAVYTAGYKICNYHLATKEDLQNAVSIMWNRDLLVVESKAQGTDSIARCNCNAGVYYCESRRKYYPVSFVGPTFQYMEANDYYPARYQSHMLIGHGFASPGDCGGILRCQHGVIGIVTAGGEGLVAFSDIRDLYAYEEEAMEQ
472. GFGHQNKAVYTAGYKICNYHLATKEDLQNAVSIMWNRDLLVVESKAQGTDSIARCNCNAGVYYCESRRKYYPVSFVGPTFQYMEANDYYPARYQSHMLIGHGFASPGDCGGILRCQHGVIGIVTAGGEGLVAFSDIRDLYAYEEEAMEQ
473. GFGHQNKAVYTAGYKICNYHLATKEDLQNAVSIMWNRDLLVVESKAQGTDSIARCNCNAGVYYCESRRKYYPVSFVGPTFQYMEANDYYPARYQSHMLIGHGFASPGDCGGILRCQHGVIGIVTAGGEGLVAFSDIRDLYAYEEEAMEQ
474. GFGHQNKAVYTAGYKICNYHLATKEDLQNAVSIMWNRDLLVVESKAQGTDSIARCNCNAGVYYCESRRKYYPVSFVGPTFQYMEANDYYPARYQSHMLIGHGFASPGDCGGILRCQHGVIGIVTAGGEGLVAFSDIRDLYAYEEEAMEQ
475. GFGHQNKAVYTAGYKICNYHLATKEDLQNAVSIMWNRDLLVVESKAQGTDSIARCNCNAGVYYCESRRKYYPVSFVGPTFQYMEANDYYPARYQSHMLIGHGFASPGDCGGILRCQHGVIGIVTAGGEGLVAFSDIRDLYAYEEEAMEQ
476. GFGHQNKAVYTAGYKICNYHLATKEDLQNAVSIMWNRDLLVVESKAQGTDSIARCNCNAGVYYCESRRKYYPVSFVGPTFQYMEANDYYPARYQSHMLIGHGFASPGDCGGILRCQHGVIGIVTAGGEGLVAFSDIRDLYAYEEEAMEQ
477. GFGHQNKAVYTAGYKICNYHLATKEDLQNAVSIMWNRDLLVVESKAQGTDSIARCNCNAGVYYCESRRKYYPVSFVGPTFQYMEANDYYPARYQSHMLIGHGFASPGDCGGILRCQHGVIGIVTAGGEGLVAFSDIRDLYAYEEEAMEQ
478. GFGHQNKAVYTAGYKICNYHLATKEDLQNAVSIMWNRDLLVVESKAQGTDSIARCNCNAGVYYCESRRKYYPVSFVGPTFQYMEANDYYPARYQSHMLIGHGFASPGDCGGILRCQHGVIGIVTAGGEGLVAFSDIRDLYAYEEEAMEQ
479. GFGHQNKAVYTAGYKICNYHLATKEDLQNAVSIMWNRDLLVVESKAQGTDSIARCNCNAGVYYCESRRKYYPVSFVGPTFQYMEANDYYPARYQSHMLIGHGFASPGDCGGILRCQHGVIGIVTAGGEGLVAFSDIRDLYAYEEEAMEQ
480. GFGHQNKAVYTAGYKICNYHLATKEDLQNAVSIMWNRDLLVVESKAQGTDSIARCNCNAGVYYCESRRKYYPVSFVGPTFQYMEANDYYPARYQSHMLIGHGFASPGDCGGILRCQHGVIGIVTAGGEGLVAFSDIRDLYAYEEEAMEQ
481. GFGHQNKAVYTAGYKICNYHLATKEDLQNAVSIMWNRDLLVVESKAQGTDSIARCNCNAGVYYCESRRKYYPVSFVGPTFQYMEANDYYPARYQSHMLIGHGFASPGDCGGILRCQHGVIGIVTAGGEGLVAFSDIRDLYAYEEEAMEQ
482. GFGHQNKAVYTAGYKICNYHLATKEDLQNAVSIMWNRDLLVVESKAQGTDSIARCNCNAGVYYCESRRKYYPVSFVGPTFQYMEANDYYPARYQSHMLIGHGFASPGDCGGILRCQHGVIGIVTAGGEGLVAFSDIRDLYAYEEEAMEQ
483. GFGHQNKAVYTAGYKICNYHLATKEDLQNAVSIMWNRDLLVVESKAQGTDSIARCNCNAGVYYCESRRKYYPVSFVGPTFQYMEANDYYPARYQSHMLIGHGFASPGDCGGILRCQHGVIGIVTAGGEGLVAFSDIRDLYAYEEEAMEQ
484. GFGHQNKAVYTAGYKICNYHLATKEDLQNAVSIMWNRDLLVVESKAQGTDSIARCNCNAGVYYCESRRKYYPVSFVGPTFQYMEANDYYPARYQSHMLIGHGFASPGDCGGILRCQHGVIGIVTAGGEGLVAFSDIRDLYAYEEEAMEQ
485. GFGHQNKAVYTAGYKICNYHLATKEDLQNAVSIMWNRDLLVVESKAQGTDSIARCNCNAGVYYCESRRKYYPVSFVGPTFQYMEANDYYPARYQSHMLIGHGFASPGDCGGILRCQHGVIGIVTAGGEGLVAFSDIRDLYAYEEEAMEQ
486. GFGHQNKAVYTAGYKICNYHLATKEDLQNAVSIMWNRDLLVVESKAQGTDSIARCNCNAGVYYCESRRKYYPVSFVGPTFQYMEANDYYPARYQSHMLIGHGFASPGDCGGILRCQHGVIGIVTAGGEGLVAFSDIRDLYAYEEEAMEQ
487. GFGHQNKAVYTAGYKICNYHLATKEDLQNAVSIMWNRDLLVVESKAQGTDSIARCNCNAGVYYCESRRKYYPVSFVGPTFQYMEANDYYPARYQSHMLIGHGFASPGDCGGILRCQHGVIGIVTAGGEGLVAFSDIRDLYAYEEEAMEQ
488. GFGHQNKAVYTAGYKICNYHLATKEDLQNAVSIMWNRDLLVVESKAQGTDSIARCNCNAGVYYCESRRKYYPVSFVGPTFQYMEANDYYPARYQSHMLIGHGFASPGDCGGILRCQHGVIGIVTAGGEGLVAFSDIRDLYAYEEEAMEQ
489. GFGHQNKAVYTAGYKICNYHLATKEDLQNAVSIMWNRDLLVVESKAQGTDSIARCNCNAGVYYCESRRKYYPVSFVGPTFQYMEANDYYPARYQSHMLIGHGFASPGDCGGILRCQHGVIGIVTAGGEGLVAFSDIRDLYAYEEEAMEQ
490. GFGHQNKAVYTAGYKICNYHLATKEDLQNAVSIMWNRDLLVVESKAQGTDSIARCNCNAGVYYCESRRKYYPVSFVGPTFQYMEANDYYPARYQSHMLIGHGFASPGDCGGILRCQHGVIGIVTAGGEGLVAFSDIRDLYAYEEEAMEQ
491. GFGHQNKAVYTAGYKICNYHLATKEDLQNAVSIMWNRDLLVVESKAQGTDSIARCNCNAGVYYCESRRKYYPVSFVGPTFQYMEANDYYPARYQSHMLIGHGFASPGDCGGILRCQHGVIGIVTAGGEGLVAFSDIRDLYAYEEEAMEQ
492. GFGHQNKAVYTAGYKICNYHLATKEDLQNAVSIMWNRDLLVVESKAQGTDSIARCNCNAGVYYCESRRKYYPVSFVGPTFQYMEANDYYPARYQSHMLIGHGFASPGDCGGILRCQHGVIGIVTAGGEGLVAFSDIRDLYAYEEEAMEQ
493. GFGHQNKAVYTAGYKICNYHLATKEDLQNAVSIMWNRDLLVVESKAQGTDSIARCNCNAGVYYCESRRKYYPVSFVGPTFQYMEANDYYPARYQSHMLIGHGFASPGDCGGILRCQHGVIGIVTAGGEGLVAFSDIRDLYAYEEEAMEQ
494. GFGHQNKAVYTAGYKICNYHLATKEDLQNAVSIMWNRDLLVVESKAQGTDSIARCNCNAGVYYCESRRKYYPVSFVGPTFQYMEANDYYPARYQSHMLIGHGFASPGDCGGILRCQHGVIGIVTAGGEGLVAFSDIRDLYAYEEEAMEQ
495. GFGHQNKAVYTAGYKICNYHLATKEDLQNAVSIMWNRDLLVVESKAQGTDSIARCNCNAGVYYCESRRKYYPVSFVGPTFQYMEANDYYPARYQSHMLIGHGFASPGDCGGILRCQHGVIGIVTAGGEGLVAFSDIRDLYAYEEEAMEQ
496. GFGHQNKAVYTAGYKICNYHLATKEDLQNAVSIMWNRDLLVVESKAQGTDSIARCNCNAGVYYCESRRKYYPVSFVGPTFQYMEANDYYPARYQSHMLIGHGFASPGDCGGILRCQHGVIGIVTAGGEGLVAFSDIRDLYAYEEEAMEQ
497. GFGHQNKAVYTAGYKICNYHLATKEDLQNAVSIMWNRDLLVVESKAQGTDSIARCNCNAGVYYCESRRKYYPVSFVGPTFQYMEANDYYPARYQSHMLIGHGFASPGDCGGILRCQHGVIGIVTAGGEGLVAFSDIRDLYAYEEEAMEQ
498. GFGHQNKAVYTAGYKICNYHLATKEDLQNAVSIMWNRDLLVVESKAQGTDSIARCNCNAGVYYCESRRKYYPVSFVGPTFQYMEANDYYPARYQSHMLIGHGFASPGDCGGILRCQHGVIGIVTAGGEGLVAFSDIRDLYAYEEEAMEQ
499. GFGHQNKAVYTAGYKICNYHLATKEDLQNAVSIMWNRDLLVVESKAQGTDSIARCNCNAGVYYCESRRKYYPVSFVGPTFQYMEANDYYPARYQSHMLIGHGFASPGDCGGILRCQHGVIGIVTAGGEGLVAFSDIRDLYAYEEEAMEQ
500. GFGHQNKAVYTAGYKICNYHLATKEDLQNAVSIMWNRDLLVVESKAQGTDSIARCNCNAGVYYCESRRKYYPVSFVGPTFQYMEANDYYPARYQSHMLIGHGFASPGDCGGILRCQHGVIGIVTAGGEGLVAFSDIRDLYAYEEEAMEQ
501. GFGHQNKAVYTAGYKICNYHLATKEDLQNAVSIMWNRDLLVVESKAQGTDSIARCNCNAGVYYCESRRKYYPVSFVGPTFQYMEANDYYPARYQSHMLIGHGFASPGDCGGILRCQHGVIGIVTAGGEGLVAFSDIRDLYAYEEEAMEQ
502. GFGHQNKAVYTAGYKICNYHLATKEDLQNAVSIMWNRDLLVVESKAQGTDSIARCNCNAGVYYCESRRKYYPVSFVGPTFQYMEANDYYPARYQSHMLIGHGFASPGDCGGILRCQHGVIGIVTAGGEGLVAFSDIRDLYAYEEEAMEQ
503. GFGHQNKAVYTAGYKICNYHLATKEDLQNAVSIMWNRDLLVVESKAQGTDSIARCNCNAGVYYCESRRKYYPVSFVGPTFQYMEANDYYPARYQSHMLIGHGFASPGDCGGILRCQHGVIGIVTAGGEGLVAFSDIRDLYAYEEEAMEQ
504. GFGHQNKAVYTAGYKICNYHLATKEDLQNAVSIMWNRDLLVVESKAQGTDSIARCNCNAGVYYCESRRKYYPVSFVGPTFQYMEANDYYPARYQSHMLIGHGFASPGDCGGILRCQHGVIGIVTAGGEGLVAFSDIRDLYAYEEEAMEQ
505. GFGHQNKAVYTAGYKICNYHLATKEDLQNAVSIMWNRDLLVVESKAQGTDSIARCNCNAGVYYCESRRKYYPVSFVGPTFQYMEANDYYPARYQSHMLIGHGFASPGDCGGILRCQHGVIGIVTAGGEGLVAFSDIRDLYAYEEEAMEQ
506. GFGHQNKAVYTAGYKICNYHLATKEDLQNAVSIMWNRDLLVVESKAQGTDSIARCNCNAGVYYCESRRKYYPVSFVGPTFQYMEANDYYPARYQSHMLIGHGFASPGDCGGILRCQHGVIGIVTAGGEGLVAFSDIRDLYAYEEEAMEQ
507. GFGHQNKAVYTAGYKICNYHLATKEDLQNAVSIMWNRDLLVVESKAQGTDSIARCNCNAGVYYCESRRKYYPVSFVGPTFQYMEANDYYPARYQSHMLIGHGFASPGDCGGILRCQHGVIGIVTAGGEGLVAFSDIRDLYAYEEEAMEQ
508. GFGHQNKAVYTAGYKICNYHLATKEDLQNAVSIMWNRDLLVVESKAQGTDSIARCNCNAGVYYCESRRKYYPVSFVGPTFQYMEANDYYPARYQSHMLIGHGFASPGDCGGILRCQHGVIGIVTAGGEGLVAFSDIRDLYAYEEEAMEQ
509. GFGHQNKAVYTAGYKICNYHLATKEDLQNAVSIMWNRDLLVVESKAQGTDSIARCNCNAGVYYCESRRKYYPVSFVGPTFQYMEANDYYPARYQSHMLIGHGFASPGDCGGILRCQHGVIGIVTAGGEGLVAFSDIRDLYAYEEEAMEQ
510. GFGHQNKAVYTAGYKICNYHLATKEDLQNAVSIMWNRDLLVVESKAQGTDSIARCNCNAGVYYCESRRKYYPVSFVGPTFQYMEANDYYPARYQSHMLIGHGFASPGDCGGILRCQHGVIGIVTAGGEGLVAFSDIRDLYAYEEEAMEQ
511. GFGHQNKAVYTAGYKICNYHLATKEDLQNAVSIMWNRDLLVVESKAQGTDSIARCNCNAGVYYCESRRKYYPVSFVGPTFQYMEANDYYPARYQSHMLIGHGFASPGDCGGILRCQHGVIGIVTAGGEGLVAFSDIRDLYAYEEEAMEQ
512. GFGHQNKAVYTAGYKICNYHLATKEDLQNAVSIMWNRDLLVVESKAQGTDSIARCNCNAGVYYCESRRKYYPVSFVGPTFQYMEANDYYPARYQSHMLIGHGFASPGDCGGILRCQHGVIGIVTAGGEGLVAFSDIRDLYAYEEEAMEQ
513. GFGHQNKAVYTAGYKICNYHLATKEDLQNAVSIMWNRDLLVVESKAQGTDSIARCNCNAGVYYCESRRKYYPVSFVGPTFQYMEANDYYPARYQSHMLIGHGFASPGDCGGILRCQHGVIGIVTAGGEGLVAFSDIRDLYAYEEEAMEQ
514. GFGHQNKAVYTAGYKICNYHLATKEDLQNAVSIMWNRDLLVVESKAQGTDSIARCNCNAGVYYCESRRKYYPVSFVGPTFQYMEANDYYPARYQSHMLIGHGFASPGDCGGILRCQHGVIGIVTAGGEGLVAFSDIRDLYAYEEEAMEQ
515. GFGHQNKAVYTAGYKICNYHLATKEDLQNAVSIMWNRDLLVVESKAQGTDSIARCNCNAGVYYCESRRKYYPVSFVGPTFQYMEANDYYPARYQSHMLIGHGFASPGDCGGILRCQHGVIGIVTAGGEGLVAFSDIRDLYAYEEEAMEQ
516. GFGHQNKAVYTAGYKICNYHLATKEDLQNAVSIMWNRDLLVVESKAQGTDSIARCNCNAGVYYCESRRKYYPVSFVGPTFQYMEANDYYPARYQSHMLIGHGFASPGDCGGILRCQHGVIGIVTAGGEGLVAFSDIRDLYAYEEEAMEQ
517. GFGHQNKAVYTAGYKICNYHLATKEDLQNAVSIMWNRDLLVVESKAQGTDSIARCNCNAGVYYCESRRKYYPVSFVGPTFQYMEANDYYPARYQSHMLIGHGFASPGDCGGILRCQHGVIGIVTAGGEGLVAFSDIRDLYAYEEEAMEQ
518. GFGHQNKAVYTAGYKICNYHLATKEDLQNAVSIMWNRDLLVVESKAQGTDSIARCNCNAGVYYCESRRKYYPVSFVGPTFQYMEANDYYPARYQSHMLIGHGFASPGDCGGILRCQHGVIGIVTAGGEGLVAFSDIRDLYAYEEEAMEQ
519. GFGHQNKAVYTAGYKICNYHLATKEDLQNAVSIMWNRDLLVVESKAQGTDSIARCNCNAGVYYCESRRKYYPVSFVGPTFQYMEANDYYPARYQSHMLIGHGFASPGDCGGILRCQHGVIGIVTAGGEGLVAFSDIRDLYAYEEEAMEQ
520. GFGHQNKAVYTAGYKICNYHLATKEDLQNAVSIMWNRDLLVVESKAQGTDSIARCNCNAGVYYCESRRKYYPVSFVGPTFQYMEANDYYPARYQSHMLIGHGFASPGDCGGILRCQHGVIGIVTAGGEGLVAFSDIRDLYAYEEEAMEQ
521. GFGHQNKAVYTAGYKICNYHLATKEDLQNAVSIMWNRDLLVVESKAQGTDSIARCNCNAGVYYCESRRKYYPVSFVGPTFQYMEANDYYPARYQSHMLIGHGFASPGDCGGILRCQHGVIGIVTAGGEGLVAFSDIRDLYAYEEEAMEQ
522. GFGHQNKAVYTAGYKICNYHLATKEDLQNAVSIMWNRDLLVVESKAQGTDSIARCNCNAGVYYCESRRKYYPVSFVGPTFQYMEANDYYPARYQSHMLIGHGFASPGDCGGILRCQHGVIGIVTAGGEGLVAFSDIRDLYAYEEEAMEQ
523. GFGHQNKAVYTAGYKICNYHLATKEDLQNAVSIMWNRDLLVVESKAQGTDSIARCNCNAGVYYCESRRKYYPVSFVGPTFQYMEANDYYPARYQSHMLIGHGFASPGDCGGILRCQHGVIGIVTAGGEGLVAFSDIRDLYAYEEEAMEQ
524. GFGHQNKAVYTAGYKICNYHLATKEDLQNAVSIMWNRDLLVVESKAQGTDSIARCNCNAGVYYCESRRKYYPVSFVGPTFQYMEANDYYPARYQSHMLIGHGFASPGDCGGILRCQHGVIGIVTAGGEGLVAFSDIRDLYAYEEEAMEQ
525. GFGHQNKAVYTAGYKICNYHLATKEDLQNAVSIMWNRDLLVVESKAQGTDSIARCNCNAGVYYCESRRKYYPVSFVGPTFQYMEANDYYPARYQSHMLIGHGFASPGDCGGILRCQHGVIGIVTAGGEGLVAFSDIRDLYAYEEEAMEQ
526. GFGHQNKAVYTAGYKICNYHLATKEDLQNAVSIMWNRDLLVVESKARGTDSIARCNCNAGVYYCESRRKYYPVSFVGPTFQYMEANDYYPARYQSHMLIGHGFASPGDCGGILRCQHGVIGIVTAGGEGLVAFSDIRDLYAYEEEAMEQ
527. GFGHQNKAVYTAGYKICNYHLATKEDLQNAVSIMWNRDLLVVESKAQGTDSIARCNCNAGVYYCESRRKYYPVSFVGPTFQYMEANDYYPARYQSHMLIGHGFASPGDCGGILRCQHGVIGIVTAGGEGLVAFSDIRDLYAYEEEAMEQ
528. GFGHQNKAVYTAGYKICNYHLATKEDLQNAVSIMWNRDLLVVESKAQGTDSIARCNCNAGVYYCESRRKYYPVSFVGPTFQYMEANDYYPARYQSHMLIGHGFASPGDCGGILRCQHGVIGIVTAGGEGLVAFSDIRDLYAYEEEAMEQ
529. GFGHQNKAVYTAGYKICNYHLATKEDLLNAVSIMWNRDLLVVESKAQGTDSIARCNCNAGVYYCESRRKYYPVSFVGPTFQYMEANDYYPARYQSHMLIGHGFASPGDCGGILRCQHGVIGIVTAGGEGLVAFSDIRDLYAYEEEAMEQ
530. GFGHQNKAVYTAGYKICNYHLATQEDLQNAVSVMWNRDLLVAESRALGTDSIARCSCNTGVYYCESRRKYYPVSFIGPTFQYMEANEYYPARYQSHMLIGHGFASPGDCGGILRCQHGVIGIITAGGEGLVAFSDIRDLYAYEEEAMEQ
531. GFGHQNKAVYTAGYKICNYHLATQEDLQNAVSVMWNRDLLVAESRALGTDSIARCSCNTGVYYCESRRKYYPVSFIGPTFQYMEANEYYPARYQSHMLIGHGFASPGDCGGILRCQHGVIGIITAGGEGLVAFSDIRDLYAYEEEAMEQ
532. GFGHQNKAVYTAGYKICNYHLATQEDLQNAVSVMWNRDLLVAESRALGTDSIARCGCNTGVYYCESRRKYYPVSFIGPTFQYMEANEYYPARYQSHMLIGHGFASPGDCGGILRCQHGVIGIITAGGEGLVAFSDIRDLYAYEEEAMEQ
533. GFGHQNKAVYTAGYKICNYHLATQEDLQNAVSVMWNRDLLVAESRALGTDSIARCSCNTGVYYCESRRKHYPVSFIGPTFQYMEANEYYPARYQSHMLIGHGFASPGDCGGILRCQHGVIGIITAGGEGLVAFSDIRDLYAYEEEAMEQ
534. GFGHQNKAVYTAGYKICNYHLATQEDLQNAVSVMWNRDLLVAESRALGTDSIARCSCNTGVYYCESRRKYYPVSFIGPTFQYMEANEYYPARYQSHMLIGHGFASPGDCGGILRCQHGVIGIITAGGEGLVAFSDIRDLYAYEEEAMEQ
535. GFGHQNKAVYTAGYKICNYHLATQEDLQNAVSVMWNRDLLVAESRALGTDSIARCSCNTGVYYCESRRKYYPVSFIGPTFQYMEANEYYPARYQSHMLIGHGFASPGDCGGILRCQHGVIGIITAGGEGLVAFSDIRDLYAYEEEAMEQ
536. GFGHQNKAVYTAGYKICNYHLATQEDLQNAVSVMWNRDLLVAESRALGTDSIARCSCNTGVYYCESRRKYYPVSFIGPTFQYMEANEYYPARYQSHMLIGHGFASPGDCGGILRCQHGVIGIITAGGEGLVAFSDIRDLYAYEEEAMEQ
537. GFGHQNKAVYTAGYKICNYHLATQEDLQNAVSVMWNRDLLVAESRALGTDSIARCSCNTGVYYCESRRKYYPVSFIGPTFQYMEANEYYPARYQSHMLIGHGFASPGDCGGILRCQHGVIGIITAGGEGLVAFSDIRDLYAYEEEAMEQ
538. GFGHQNKAVYTAGYKICNYHLATQEDLQNAVSVMWNRDLLVAESRALGSDSIARCSCNTGVYYCESRRKYYPVSFIGPTFQYMEANEYYPARYQSHMLIGHGFASPGDCGGILRCQHGVIGIITAGGEGLVAFSDIRDLYAYEEEAMEQ
539. GFGHQNKAVYTAGYKICNYHLATQEDLQNAVSVMWNRDLLVAESRALGTDSIARCSCNTGVYYCESRRKYYPVSFIGPTFQYMEANEYYPARYQSHMLIGHGFASPGDCGGILRCQHGVIGIITAGGEGLVAFSDIRDLYAYEEEAMEQ
540. GFGHQNKAVYTAGYKICNYHLATQEDLQNAVSVMWSRDLLVTESRAQGIDSIARCNCSTGVYYCESRRRYYPVSFVGPTFQYMEANDYYPARYQSHMLIGHGFASPGDCGGILRCQHGVIGIITAGGEGLVAFSDIRDLYAYEEEAMEQ
541. GFGHQNKAVYTAGYKICNYHLATQEDLQNAVSVMWNRDLLVTESKAQGIDSIARCNCSTGVYYCESRRRYYPVSFVGPTFQYMEANDYYPARYQSHMLIGHGFASPGDCGGILRCQHGVIGIITAGGEGLVAFSDIRDLYAYEEEAMEQ
542. GFGHQNKAVYTAGYKICNYHLATKEDLQNAVSIMWNRDLLVVESNAQGTDSIARCNCNVGVYYCESRRKYYPVSFVGPTFQYMEANDYYPARYQSHMLIGHGFASPGDCGGILRCQHGVIGIVTAGGEGLVAFSDIRDLYAYEEEAMEQ
543. GFGHQNKAVYTAGYKICNYHLATKEDLQNAVSIMWNRDLLVVESKAQGTDSIARCNCNAGVYYCESRRKYYPVSFVGPTFQYMEANDYYPARYQSHMLIGHGFASPGDCGGILRCQHGVIGIVTAGGEGLVAFSDIRDLYAYEEEAMEQ
544. GFGHQNKAVYTAGYKICNYHLATKEDLQNAVSIMWNRDLLVVESKAQGTDSIARCNCNAGVYYCESRRKYYPVSFVGPTFQYMEANDYYPARYQSHMLIGHGFASPGDCGGILRCQHGVIGIVTAGGEGLVAFSDIRDLYAYEEEAMEQ
545. GFGHQNKAVYTAGYKICNYHLATKEDLQNAVSIMWNRDLLVVESKAQGTDSIARCNCNAGVYYCESRRKYYPVSFVGPTFQYMEANDYYPARYQSHMLIGHGFASPGDCGGILRCQHGVIGIVTAGGEGLVAFSDIRDLYAYEEEAMEQ
546. GFGHQNKAVYTAGYKICNYHLATQEDLQNAVSVMWNRDLLVAESRALGTDSIARCSCNTGVYYCESRRKYYPVSFIGPTFQYMEANEYYPARYQSHMLIGHGFASPGDCGGILRCQHGVIGIITAGGEGLVAFSDIRDLYAYEEEAMEQ
547. GFGHQNKAVYTAGYKICNYHLATQEDLQNAVSVMWSRDLLVTESKAQGIDSIARCNCNTGVYYCESRRRYYPVSFIGPTFQYMEANDYYPARYQSHMLIGHGFASPGDCGGILRCQHGVIGIITAGGEGLVAFSDIRDLYAYEEEAMEQ
548. GFGHQNKAVYTAGYKICNYHLATQEDLQNAVSVMWNRDLLVAESRALGTDSIARCSCNTGVYYCESRRKYYPVSFIGPTFQYMEANEYYPARYQSHMLIGHGFASPGDCGGILRCQHGVIGIITAGGEGLVAFSDIRDLYAYEEEAMEQ
549. GFGHQNKAVYTAGYKICNYHLATKEDLQNAVSIMWNRDLLVVESKAQGTDSIARCNCNAGVYYCESRRKYYPVSFVGPTFQYMEANDYYPARYQSHMLIGHGFASPGDCGGILRCQHGVIGIVTAGGEGLVAFSDIRDLYAYEEEAMEQ
550. GFGHQNKAVYTAGYKICNYHLATKEDLQNAVSIMWNRDLLVVESKAQGTDSIARCNCNAGVYYCESRRKYYPVSFVGPTFQYMEANDYYPARYQSHMLIGHGFASPGDCGGILRCQHGVIGIVTAGGEGLVAFSDIRDLYAYEEEAMEQ
551. GFGHQNKAVYTAGYKICNYHLATKEDLQNTVSIMWNRDLLVVESKAQGTDSIARCNCNAGVYYCESRRKYYPVSFVGPTFQYMEANDYYPARYQSHMLIGHGFASPGDCGGILRCQHGVIGIVTAGGEGLVAFSDIRDLYAYEEEAMEQ
552. GFGHQNKAVYTAGYKICNYHLATQEDLQNAVSVMWNRDLLVTESKAQGIDSIARCNCSTGVYYCESRSRYYPVSFVGPTFQYMEANDYYPARYQSHMLIGHGFASPGDCGGILRCQHGVIGIITAGGEGLVAFSDIRDLYAYEEEAMEQ
553. GFGHQNKAVYTAGYKICNYHLATKEDLQNAVSIMWNRDLLVVESKAQGTDSIARCNCNAGVYYCESRRKYYPVSFVGPTFQYMEANDYYPARYQSHMLIGHGFASPGDCGGILRCQHGVIGIVTAGGEGLVAFSDIRDLYAYEEEAMEQ

Consensus GFGHQNKAVYTAGYKICNYHLATKEDLQNAVNVMWNRDLLVTESRAQGTDSIARCNCNAGVYYCESRRKYYPVSFVGPTFQYMEANNYYPARYQSHMLIGHGFASPGDCGGILRCHHGVIGIITAGGEGLVAFSDIRDLYAYEEEAMEQ
