# Supplementary material for: Structure-Function Mutational Analysis and Prediction of the Potential Impact of High Risk Non-Synonymous Single-Nucleotide Polymorphism on Poliovirus 2A Protease Stability Using Comprehensive Informatics Approaches
Source: Genes (Basel). 2018 Apr 26;9(5):228. doi: 10.3390/genes9050228 (PMC5977168; doi:10.3390/genes9050228)

**Elastatinal Pose selection**


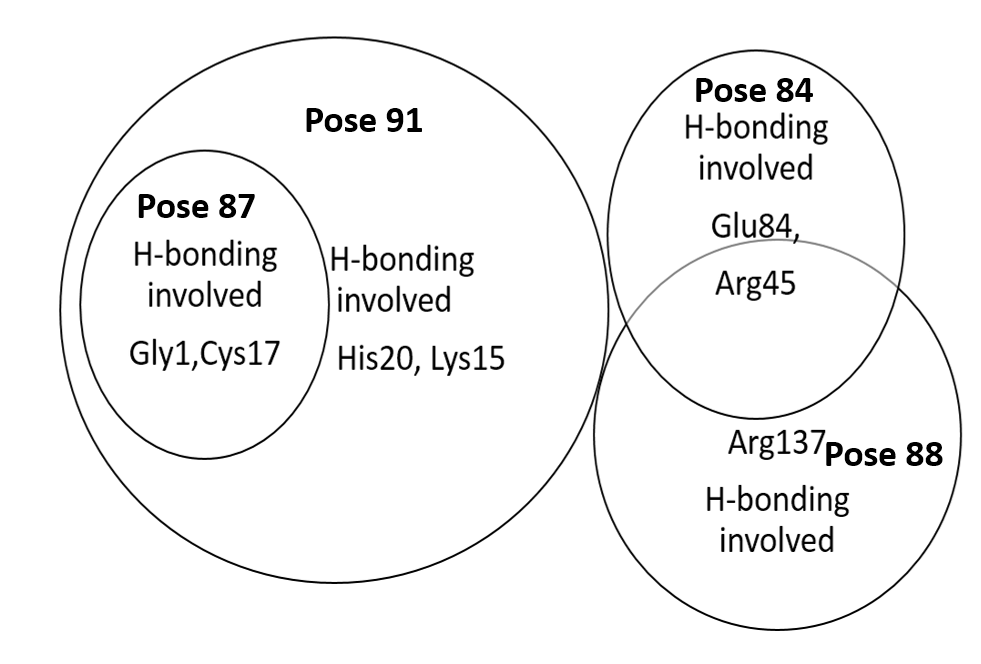


**Rupintrivir Pose selection**


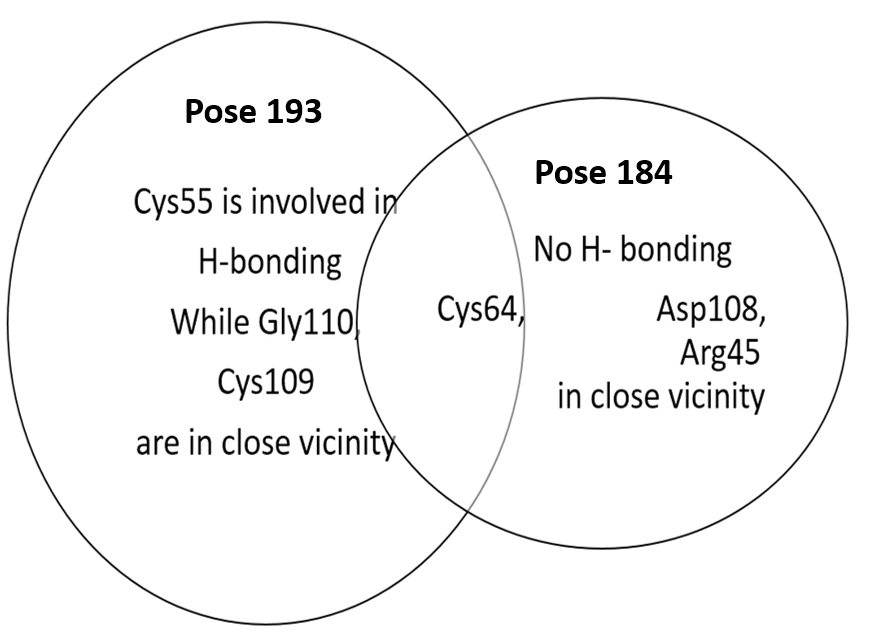


**MCPK Pose selection**


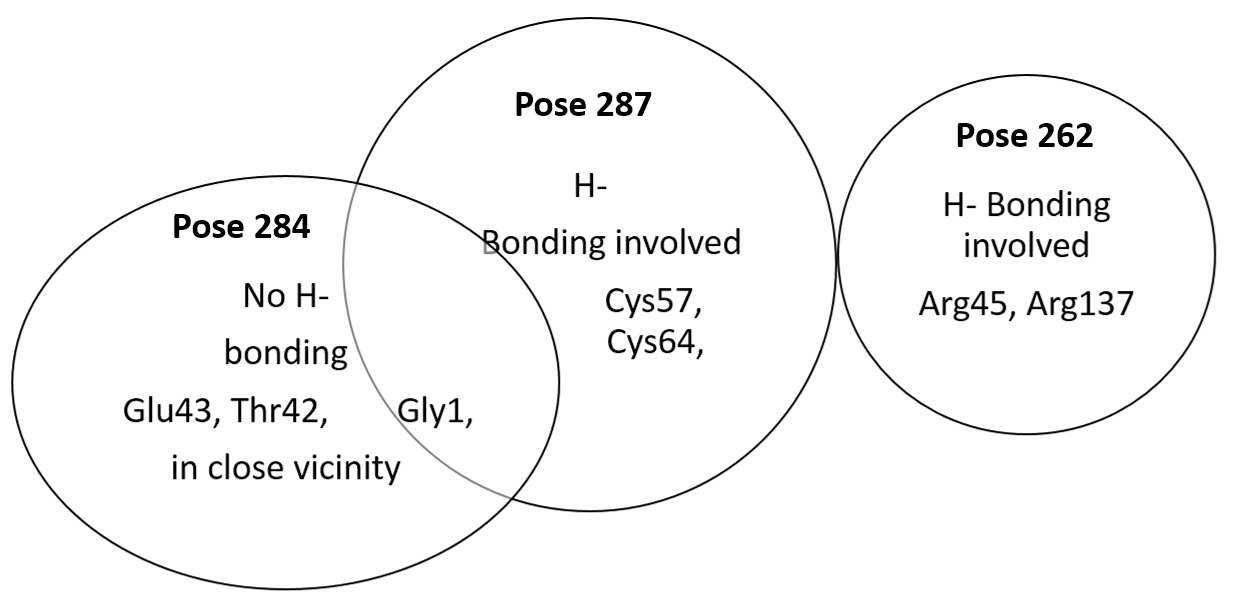


**z-VAD Pose selection**


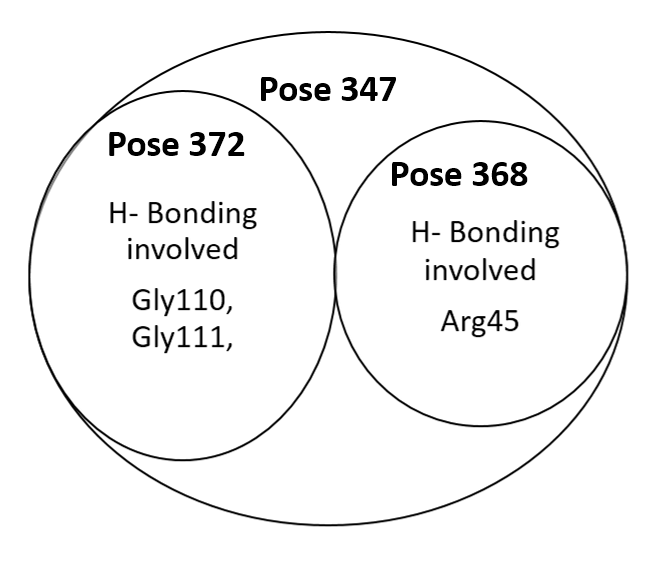

Supplement: Supplementary file 1 [file genes-09-00228-s001.zip › Supplementary material/Supplementary Fig. S4.docx]
