# Supplementary material for: Structure-Function Mutational Analysis and Prediction of the Potential Impact of High Risk Non-Synonymous Single-Nucleotide Polymorphism on Poliovirus 2A Protease Stability Using Comprehensive Informatics Approaches
Source: Genes (Basel). 2018 Apr 26;9(5):228. doi: 10.3390/genes9050228 (PMC5977168; doi:10.3390/genes9050228)
Supplement: Supplementary file 1 [file genes-09-00228-s001.zip › Supplementary material/Supplementary Table 1.docx]

**Supplementary Table 1.** Missense SNPs in PV2A^pro^ predicted to be damaging or neutral using PROVEAN, SNPs&Go, MetaSNP and PredictSNP. I-Mutant2.0 was used to calculate DDG score, RI and stability.

| **Mutation** | **PROVEAN** | **SNPs&GO** | **MetaSNP** | **PredictSNP** | **Deleterious** | **DDG** | **Stability** | **RI** |
| --- | --- | --- | --- | --- | --- | --- | --- | --- |
| G1A | Neutral | Neutral | Neutral | Neutral | Neutral |  |  |  |
| G1C | Damaging | Neutral | Neutral | Damaging | Neutral |  |  |  |
| G1D | Neutral | Neutral | Neutral | Damaging | Neutral |  |  |  |
| G1E | Neutral | Neutral | Neutral | Damaging | Neutral |  |  |  |
| G1F | Damaging | Neutral | Neutral | Damaging | Neutral |  |  |  |
| G1H | Damaging | Neutral | Neutral | Damaging | Neutral |  |  |  |
| G1I | Damaging | Neutral | Neutral | Damaging | Neutral |  |  |  |
| G1K | Neutral | Neutral | Neutral | Damaging | Neutral |  |  |  |
| G1L | Damaging | Neutral | Neutral | Damaging | Neutral |  |  |  |
| G1M | Neutral | Neutral | Neutral | Damaging | Neutral |  |  |  |
| G1N | Neutral | Neutral | Neutral | Damaging | Neutral |  |  |  |
| G1P | Neutral | Neutral | Damaging | Damaging | Neutral |  |  |  |
| G1Q | Neutral | Neutral | Neutral | Damaging | Neutral |  |  |  |
| G1R | Neutral | Neutral | Neutral | Damaging | Neutral |  |  |  |
| G1S | Neutral | Neutral | Neutral | Damaging | Neutral |  |  |  |
| G1T | Neutral | Neutral | Neutral | Damaging | Neutral |  |  |  |
| G1V | Neutral | Neutral | Neutral | Damaging | Neutral |  |  |  |
| G1W | Damaging | Neutral | Neutral | Damaging | Neutral |  |  |  |
| G1Y | Damaging | Neutral | Neutral | Damaging | Neutral |  |  |  |
| K15A | Damaging | Neutral | Damaging | Damaging | Damaging | -0.51 | Decrease | 7 |
| K15C | Damaging | Neutral | Damaging | Damaging | Damaging | -0.21 | Decrease | 1 |
| K15D | Damaging | Neutral | Damaging | Damaging | Damaging | -1.45 | Decrease | 5 |
| K15E | Damaging | Damaging | Damaging | Damaging | Damaging | -0.69 | Decrease | 5 |
| KI5F | Damaging | Neutral | Damaging | Damaging | Damaging | 1.09 | Increase | 3 |
| K15G | Damaging | Neutral | Damaging | Damaging | Damaging | -0.84 | Decrease | 7 |
| K15H | Damaging | Neutral | Damaging | Damaging | Damaging | -0.08 | Decrease | 7 |
| K15I | Damaging | Neutral | Neutral | Damaging | Neutral |  |  |  |
| K15L | Damaging | Neutral | Damaging | Damaging | Damaging | 0.47 | Increase | 1 |
| K15M | Damaging | Neutral | Neutral | Damaging | Neutral |  |  |  |
| K15N | Damaging | Neutral | Damaging | Damaging | Damaging | 0.11 | Decrease | 4 |
| K15P | Damaging | Damaging | Damaging | Damaging | Damaging | 0.7 | Increase | 5 |
| K15Q | Damaging | Neutral | Damaging | Damaging | Damaging | -0.13 | Decrease | 2 |
| K15R | Neutral | Neutral | Neutral | Neutral | Neutral |  |  |  |
| K15S | Damaging | Neutral | Neutral | Damaging | Neutral |  |  |  |
| K15T | Damaging | Neutral | Damaging | Damaging | Damaging | -0.54 | Decrease | 3 |
| K15V | Damaging | Neutral | Neutral | Damaging | Neutral |  |  |  |
| K15W | Damaging | Neutral | Damaging | Damaging | Damaging | -0.68 | Decrease | 6 |
| K15Y | Damaging | Neutral | Damaging | Damaging | Damaging | 0.71 | Increase | 4 |
| C17A | Neutral | Neutral | Damaging | Neutral | Neutral |  |  |  |
| C17D | Damaging | Damaging | Damaging | Damaging | Damaging | -1.3 | Decrease | 6 |
| C17E | Damaging | Damaging | Damaging | Damaging | Damaging | -0.28 | Decrease | 1 |
| C17F | Neutral | Neutral | Damaging | Damaging | Neutral |  |  |  |
| C17G | Damaging | Damaging | Damaging | Damaging | Damaging | -1.19 | Decrease | 8 |
| C17H | Damaging | Neutral | Damaging | Damaging | Damaging | -0.37 | Decrease | 5 |
| C17I | Neutral | Neutral | Neutral | Neutral | Neutral |  |  |  |
| C17K | Damaging | Neutral | Neutral | Neutral | Neutral |  |  |  |
| C17L | Neutral | Neutral | Neutral | Neutral | Neutral |  |  |  |
| C17M | Neutral | Neutral | Neutral | Neutral | Neutral |  |  |  |
| C17N | Damaging | Neutral | Damaging | Damaging | Damaging | -0.36 | Decrease | 6 |
| C17P | Damaging | Damaging | Neutral | Damaging | Damaging | -0.49 | Decrease | 1 |
| C17Q | Damaging | Neutral | Damaging | Damaging | Damaging | 0.06 | Decrease | 5 |
| C17R | Damaging | Neutral | Neutral | Damaging | Neutral |  |  |  |
| C17S | Damaging | Neutral | Damaging | Damaging | Damaging | -1 | Decrease | 6 |
| C17T | Neutral | Neutral | Neutral | Neutral | Neutral |  |  |  |
| C17V | Neutral | Neutral | Neutral | Neutral | Neutral |  |  |  |
| C17W | Damaging | Neutral | Damaging | Damaging | Damaging | -0.19 | Decrease | 4 |
| C17Y | Neutral | Neutral | Damaging | Damaging | Neutral |  |  |  |
| H20A | Damaging | Neutral | Damaging | Damaging | Damaging | -1.18 | Decrease | 7 |
| H20C | Damaging | Neutral | Damaging | Damaging | Damaging | 0.1 | Decrease | 2 |
| H20D | Damaging | Damaging | Damaging | Damaging | Damaging | -1.89 | Decrease | 8 |
| H20E | Damaging | Damaging | Damaging | Damaging | Damaging | -0.38 | Increase | 1 |
| H20F | Damaging | Neutral | Damaging | Damaging | Damaging | -0.05 | Decrease | 4 |
| H20G | Damaging | Neutral | Damaging | Damaging | Damaging | -0.57 | Decrease | 8 |
| H20I | Damaging | Neutral | Damaging | Damaging | Damaging | 0.1 | Decrease | 3 |
| H20K | Damaging | Damaging | Damaging | Damaging | Damaging | -0.77 | Decrease | 8 |
| H20L | Damaging | Neutral | Damaging | Damaging | Damaging | 0.26 | Increase | 0 |
| H20M | Damaging | Neutral | Damaging | Damaging | Damaging | 0.18 | Decrease | 3 |
| H20N | Damaging | Neutral | Damaging | Damaging | Damaging | -1.65 | Decrease | 8 |
| H20P | Damaging | Damaging | Damaging | Damaging | Damaging | -0.84 | Decrease | 0 |
| H20Q | Damaging | Neutral | Damaging | Damaging | Damaging | -0.9 | Decrease | 7 |
| H20R | Damaging | Neutral | Damaging | Damaging | Damaging | -0.83 | Decrease | 8 |
| H20S | Damaging | Damaging | Damaging | Damaging | Damaging | -1.14 | Decrease | 7 |
| H20T | Damaging | Neutral | Damaging | Damaging | Damaging | -1.19 | Decrease | 8 |
| H20V | Damaging | Neutral | Damaging | Damaging | Damaging | -0.3 | Decrease | 5 |
| H20W | Damaging | Neutral | Damaging | Damaging | Damaging | -0.34 | Decrease | 5 |
| H20Y | Damaging | Neutral | Damaging | Damaging | Damaging | 0.53 | Decrease | 0 |
| C55A | Damaging | Damaging | Damaging | Damaging | Damaging | -1.28 | Decrease | 8 |
| C55D | Damaging | Damaging | Damaging | Damaging | Damaging | -1.69 | Decrease | 8 |
| C55E | Damaging | Damaging | Damaging | Damaging | Damaging | -1.15 | Decrease | 5 |
| C55F | Damaging | Damaging | Damaging | Damaging | Damaging | -0.71 | Decrease | 6 |
| C55G | Damaging | Damaging | Damaging | Damaging | Damaging | -2.23 | Decrease | 8 |
| C55H | Damaging | Damaging | Damaging | Damaging | Damaging | -0.85 | Decrease | 7 |
| C55I | Damaging | Damaging | Damaging | Damaging | Damaging | -0.87 | Decrease | 5 |
| C55K | Damaging | Damaging | Damaging | Damaging | Damaging | -1.52 | Decrease | 7 |
| C55L | Damaging | Damaging | Damaging | Damaging | Damaging | -0.93 | Decrease | 4 |
| C55M | Damaging | Damaging | Damaging | Damaging | Damaging | -1.01 | Decrease | 5 |
| C55N | Damaging | Damaging | Damaging | Damaging | Damaging | -1.09 | Decrease | 7 |
| C55P | Damaging | Damaging | Damaging | Damaging | Damaging | -1.31 | Decrease | 4 |
| C55Q | Damaging | Damaging | Damaging | Damaging | Damaging | -1.18 | Decrease | 8 |
| C55R | Damaging | Damaging | Damaging | Damaging | Damaging | -1.14 | Decrease | 7 |
| C55S | Damaging | Damaging | Damaging | Damaging | Damaging | -1.86 | Decrease | 8 |
| C55T | Damaging | Damaging | Damaging | Damaging | Damaging | -1.91 | Decrease | 5 |
| C55V | Damaging | Damaging | Damaging | Damaging | Damaging | -1.05 | Decrease | 8 |
| C55W | Damaging | Damaging | Damaging | Damaging | Damaging | -0.85 | Decrease | 6 |
| C55Y | Damaging | Damaging | Damaging | Damaging | Damaging | -0.28 | Decrease | 4 |
| C64A | Damaging | Damaging | Damaging | Damaging | Damaging | -0.71 | Decrease | 7 |
| C64D | Damaging | Damaging | Damaging | Damaging | Damaging | -1.78 | Decrease | 7 |
| C64E | Damaging | Damaging | Damaging | Damaging | Damaging | -1.32 | Decrease | 5 |
| C64F | Damaging | Damaging | Damaging | Damaging | Damaging | 0.06 | Decrease | 5 |
| C64G | Damaging | Damaging | Damaging | Damaging | Damaging | -2.93 | Decrease | 8 |
| C64H | Damaging | Damaging | Damaging | Damaging | Damaging | -1.33 | Decrease | 8 |
| C64I | Damaging | Damaging | Damaging | Damaging | Damaging | -0.71 | Decrease | 7 |
| C64K | Damaging | Damaging | Damaging | Damaging | Damaging | -1.43 | Decrease | 7 |
| C64L | Damaging | Damaging | Damaging | Damaging | Damaging | -1.1 | Decrease | 6 |
| C64M | Damaging | Damaging | Damaging | Damaging | Damaging | -1.48 | Decrease | 6 |
| C64N | Damaging | Damaging | Damaging | Damaging | Damaging | -1.27 | Decrease | 8 |
| C64P | Damaging | Damaging | Damaging | Damaging | Damaging | -0.77 | Decrease | 2 |
| C64Q | Damaging | Damaging | Damaging | Damaging | Damaging | -1.34 | Decrease | 8 |
| C64R | Damaging | Damaging | Damaging | Damaging | Damaging | -0.94 | Decrease | 6 |
| C64S | Damaging | Damaging | Damaging | Damaging | Damaging | -2.93 | Decrease | 8 |
| C64T | Damaging | Damaging | Damaging | Damaging | Damaging | -2.4 | Decrease | 6 |
| C64V | Damaging | Damaging | Damaging | Damaging | Damaging | -0.36 | Decrease | 7 |
| C64W | Damaging | Damaging | Damaging | Damaging | Damaging | -0.59 | Decrease | 6 |
| C64Y | Damaging | Damaging | Damaging | Damaging | Damaging | 0.17 | Decrease | 3 |
| D108A | Damaging | Damaging | Damaging | Damaging | Damaging | -0.56 | Decrease | 2 |
| D108C | Damaging | Neutral | Damaging | Damaging | Damaging | -0.69 | Increase | 0 |
| D108E | Damaging | Damaging | Damaging | Damaging | Damaging | 0.22 | Increase | 6 |
| D108F | Damaging | Damaging | Damaging | Damaging | Damaging | -0.49 | Increase | 1 |
| D108G | Damaging | Neutral | Damaging | Damaging | Damaging | -1.07 | Decrease | 1 |
| D108H | Damaging | Neutral | Damaging | Damaging | Damaging | 0.13 | Decrease | 1 |
| D108I | Damaging | Neutral | Damaging | Damaging | Damaging | -0.76 | Increase | 3 |
| D108K | Damaging | Damaging | Damaging | Damaging | Damaging | -0.73 | Decrease | 0 |
| D108L | Damaging | Neutral | Damaging | Damaging | Damaging | -0.73 | Increase | 1 |
| D108M | Damaging | Neutral | Damaging | Damaging | Damaging | 0.04 | Increase | 4 |
| D108N | Damaging | Neutral | Damaging | Damaging | Damaging | -0.36 | Decrease | 1 |
| D108P | Damaging | Damaging | Damaging | Damaging | Damaging | -0.96 | Increase | 0 |
| D108Q | Damaging | Damaging | Damaging | Damaging | Damaging | -0.67 | Increase | 0 |
| D108R | Damaging | Neutral | Damaging | Damaging | Damaging | -0.51 | Decrease | 1 |
| D108S | Damaging | Damaging | Damaging | Damaging | Damaging | 0.49 | Increase | 7 |
| D108T | Damaging | Neutral | Damaging | Damaging | Damaging | -0.36 | Increase | 2 |
| D108V | Damaging | Damaging | Damaging | Damaging | Damaging | -0.79 | Increase | 2 |
| D108W | Damaging | Damaging | Damaging | Damaging | Damaging | -0.53 | Increase | 0 |
| D108Y | Damaging | Damaging | Damaging | Damaging | Damaging | -0.22 | Increase | 3 |
| C109A | Damaging | Damaging | Damaging | Damaging | Damaging | -0.66 | Decrease | 5 |
| C109D | Damaging | Damaging | Damaging | Damaging | Damaging | -0.59 | Decrease | 3 |
| C109E | Damaging | Damaging | Damaging | Damaging | Damaging | -0.34 | Decrease | 2 |
| C109F | Damaging | Damaging | Damaging | Damaging | Damaging | -0.57 | Decrease | 4 |
| C109G | Damaging | Damaging | Damaging | Damaging | Damaging | -1.37 | Decrease | 6 |
| C109H | Damaging | Damaging | Damaging | Damaging | Damaging | -0.34 | Decrease | 3 |
| C109I | Damaging | Damaging | Damaging | Damaging | Damaging | -0.98 | Decrease | 0 |
| C109K | Damaging | Damaging | Damaging | Damaging | Damaging | -1.07 | Decrease | 3 |
| C109L | Damaging | Damaging | Damaging | Damaging | Damaging | -1.16 | Decrease | 2 |
| C109M | Damaging | Damaging | Damaging | Damaging | Damaging | -0.94 | Decrease | 0 |
| C109N | Damaging | Damaging | Damaging | Damaging | Damaging | -0.82 | Decrease | 3 |
| C109P | Damaging | Damaging | Damaging | Damaging | Damaging | -1.1 | Decrease | 0 |
| C109Q | Damaging | Damaging | Damaging | Damaging | Damaging | -0.93 | Decrease | 3 |
| C109R | Damaging | Damaging | Damaging | Damaging | Damaging | -0.85 | Decrease | 3 |
| C109S | Damaging | Damaging | Damaging | Damaging | Damaging | -0.75 | Decrease | 2 |
| C109T | Damaging | Damaging | Damaging | Damaging | Damaging | -1.07 | Decrease | 1 |
| C109V | Damaging | Damaging | Damaging | Damaging | Damaging | -0.95 | Decrease | 3 |
| C109W | Damaging | Damaging | Damaging | Damaging | Damaging | -0.63 | Decrease | 2 |
| C109Y | Damaging | Damaging | Damaging | Damaging | Damaging | -0.3 | Decrease | 0 |
| G110A | Damaging | Damaging | Damaging | Damaging | Damaging | -1.03 | Decrease | 4 |
| G110C | Damaging | Damaging | Damaging | Damaging | Damaging | -0.97 | Decrease | 5 |
| G110D | Damaging | Damaging | Damaging | Damaging | Damaging | -0.68 | Decrease | 7 |
| G110E | Damaging | Damaging | Damaging | Damaging | Damaging | 0.16 | Decrease | 0 |
| G110F | Damaging | Damaging | Damaging | Damaging | Damaging | -0.84 | Decrease | 6 |
| G110H | Damaging | Damaging | Damaging | Damaging | Damaging | -1 | Decrease | 7 |
| G110I | Damaging | Damaging | Damaging | Damaging | Damaging | -0.64 | Decrease | 1 |
| G110K | Damaging | Damaging | Damaging | Damaging | Damaging | -1.11 | Decrease | 6 |
| G110L | Damaging | Damaging | Damaging | Damaging | Damaging | -0.89 | Decrease | 4 |
| G110M | Damaging | Neutral | Damaging | Damaging | Damaging | -0.28 | Decrease | 2 |
| G110N | Damaging | Damaging | Damaging | Damaging | Damaging | -0.46 | Decrease | 4 |
| G110P | Damaging | Damaging | Damaging | Damaging | Damaging | -0.76 | Decrease | 2 |
| G110Q | Damaging | Damaging | Damaging | Damaging | Damaging | -0.86 | Decrease | 7 |
| G110R | Damaging | Damaging | Damaging | Damaging | Damaging | -0.65 | Decrease | 6 |
| G110S | Damaging | Damaging | Damaging | Damaging | Damaging | -0.34 | Decrease | 2 |
| G110T | Damaging | Damaging | Damaging | Damaging | Damaging | -1.43 | Decrease | 7 |
| G110V | Damaging | Damaging | Damaging | Damaging | Damaging | -1.16 | Decrease | 0 |
| G110W | Damaging | Damaging | Damaging | Damaging | Damaging | -0.79 | Decrease | 6 |
| G110Y | Damaging | Damaging | Damaging | Damaging | Damaging | -0.96 | Decrease | 4 |
| C57A | Damaging | Damaging | Damaging | Damaging | Damaging | -1.4 | Decrease | 8 |
| C57D | Damaging | Damaging | Damaging | Damaging | Damaging | -1.84 | Decrease | 8 |
| C57E | Damaging | Damaging | Damaging | Damaging | Damaging | -1.44 | Decrease | 5 |
| C57F | Damaging | Damaging | Damaging | Damaging | Damaging | -0.82 | Decrease | 7 |
| C57G | Damaging | Damaging | Damaging | Damaging | Damaging | -2.75 | Decrease | 9 |
| C57H | Damaging | Damaging | Damaging | Damaging | Damaging | -1.06 | Decrease | 8 |
| C57I | Damaging | Damaging | Damaging | Damaging | Damaging | -1.08 | Decrease | 6 |
| C57K | Damaging | Damaging | Damaging | Damaging | Damaging | -1.83 | Decrease | 7 |
| C57L | Damaging | Damaging | Damaging | Damaging | Damaging | -1.18 | Decrease | 4 |
| C57M | Damaging | Damaging | Damaging | Damaging | Damaging | -1.35 | Decrease | 6 |
| C57N | Damaging | Damaging | Damaging | Damaging | Damaging | -1.8 | Decrease | 8 |
| C57P | Damaging | Damaging | Damaging | Damaging | Damaging | -1.44 | Decrease | 4 |
| C57Q | Damaging | Damaging | Damaging | Damaging | Damaging | -1.64 | Decrease | 9 |
| C57R | Damaging | Damaging | Damaging | Damaging | Damaging | -1.37 | Decrease | 8 |
| C57S | Damaging | Damaging | Damaging | Damaging | Damaging | -2.18 | Decrease | 8 |
| C57T | Damaging | Damaging | Damaging | Damaging | Damaging | -2.17 | Decrease | 5 |
| C57V | Damaging | Damaging | Damaging | Damaging | Damaging | -1.25 | Decrease | 8 |
| C57W | Damaging | Damaging | Damaging | Damaging | Damaging | -1.01 | Decrease | 7 |
| C57Y | Damaging | Damaging | Damaging | Damaging | Damaging | -0.43 | Decrease | 5 |

**Supplementary Figure S1**


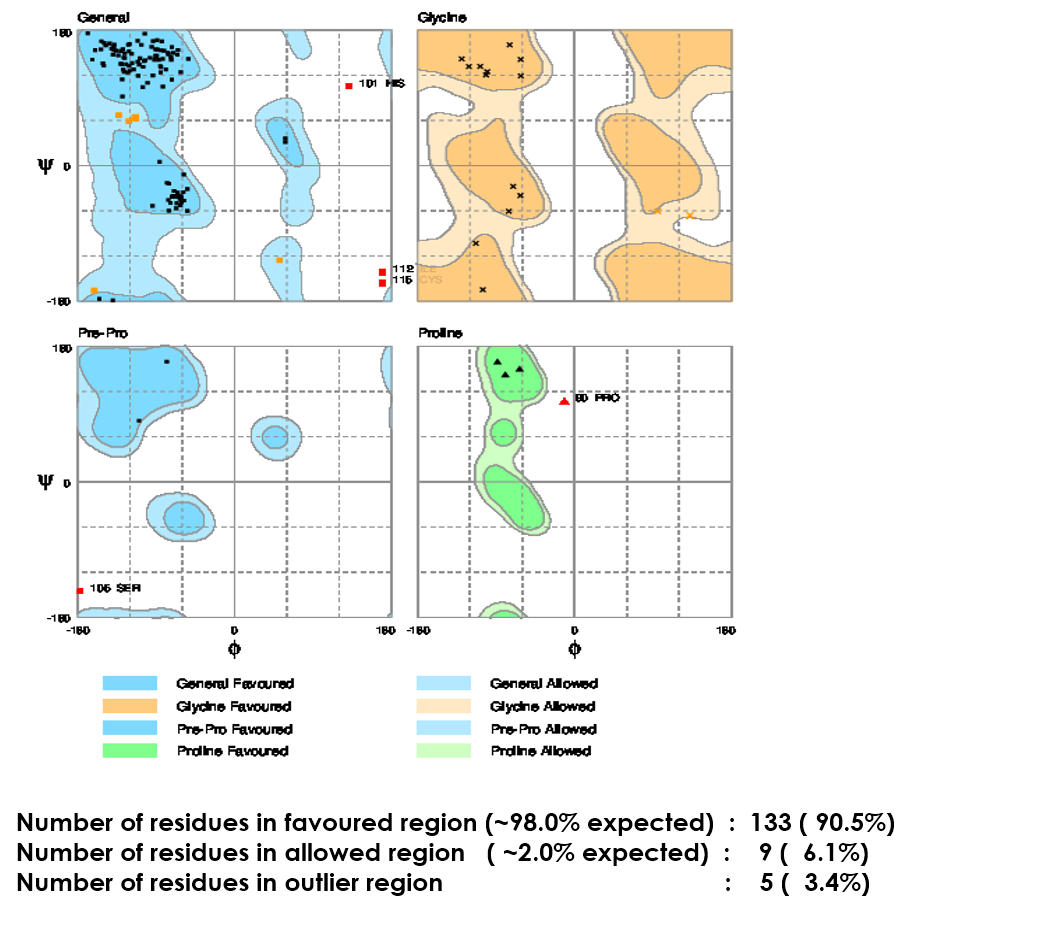


**Supplementary Fig. S1.** Ramachandran Plot analysis of poliovirus 2A protease.

Supplementary Figure 2





**Supplementary Fig. S2.** Secondary structure prediction of poliovirus 2A protease using SOPMA software.

**Supplementary Figure 3**


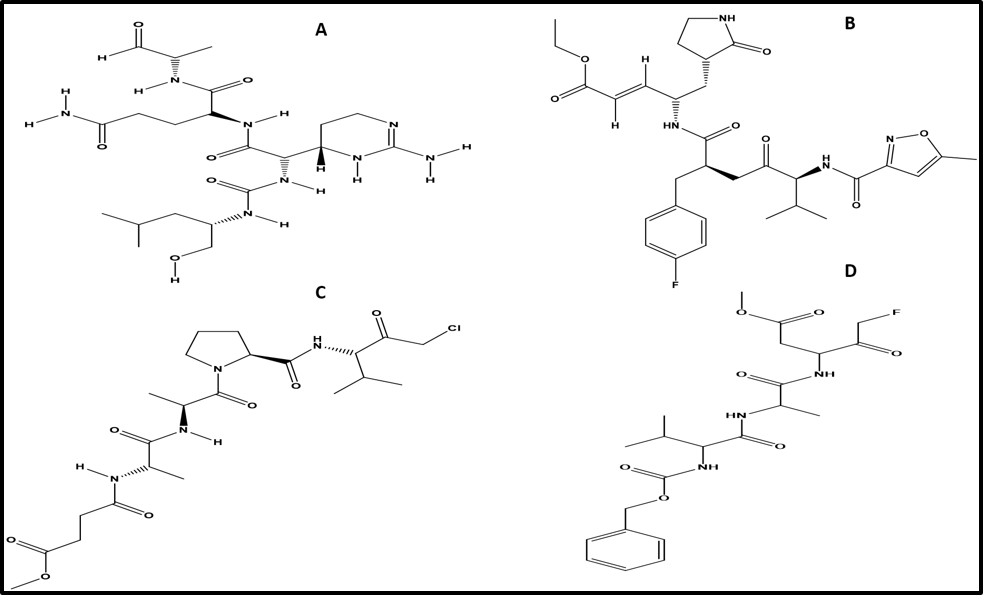


**Supplementary Fig. S3.** Chemical structures of ligands used for docking studies against poliovirus 2Apro by using ChemDraw software. A: Elastatinal (IC50=7µM), B: Rupintrivir (IC50-1.6µM), C: Methoxysuccinyl-ala-ala-pro-val-chloromethylketone (MCPK) (IC50=65µM), D: Z-VAD (OMe)-FMK (IC50=5.6µM).

Supplementary Figure 4

**Elastatinal Pose selection**


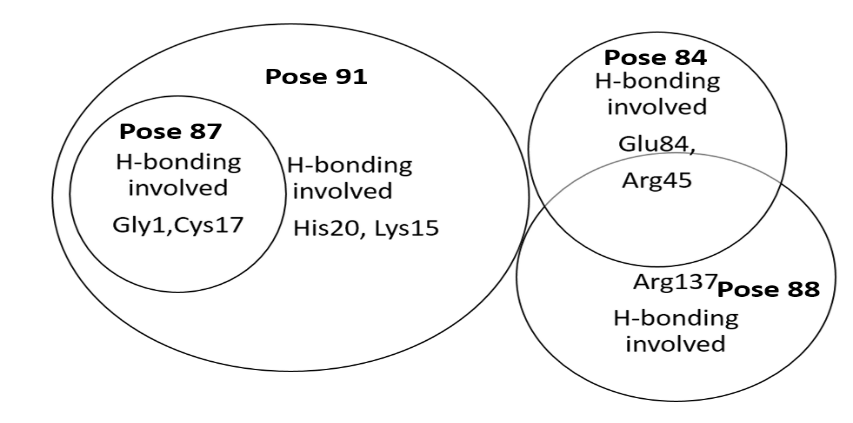


**Rupintrivir Pose selection**


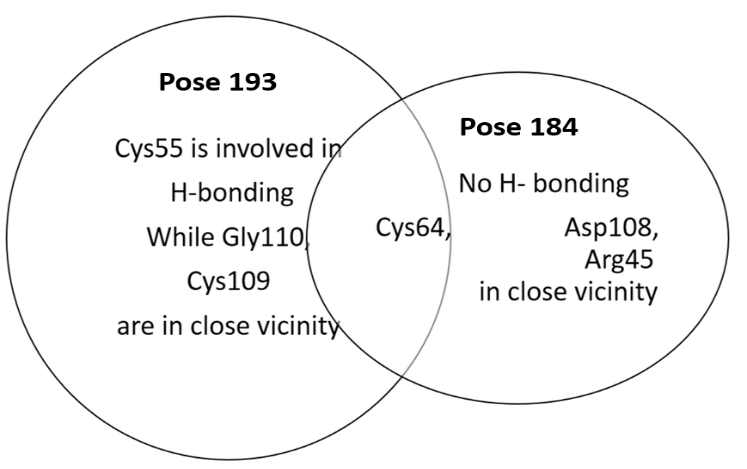


**MCPK Pose selection**


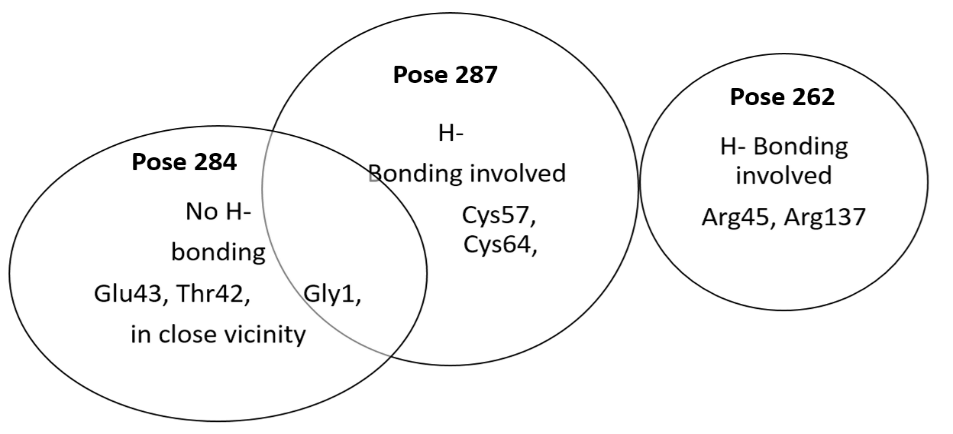


**z-VAD Pose selection**


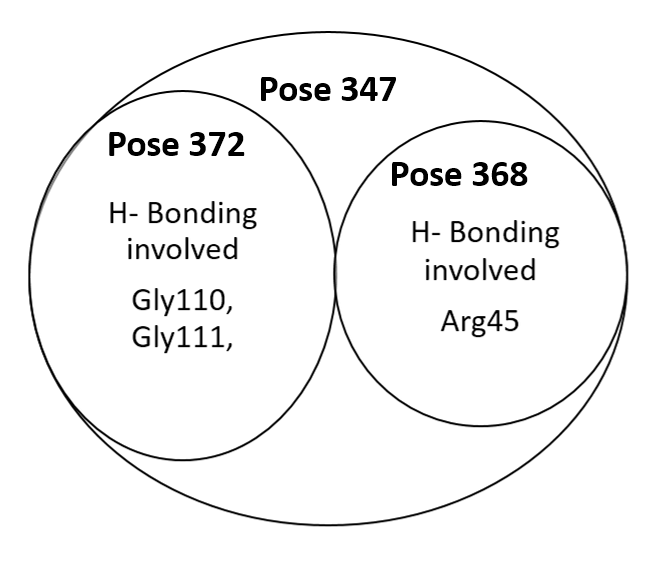


**Supplementary Fig. S4.** Final Pose selection of 2Aprotease binding after docking simulations
