# Supplementary material for: Structure-Function Mutational Analysis and Prediction of the Potential Impact of High Risk Non-Synonymous Single-Nucleotide Polymorphism on Poliovirus 2A Protease Stability Using Comprehensive Informatics Approaches
Source: Genes (Basel). 2018 Apr 26;9(5):228. doi: 10.3390/genes9050228 (PMC5977168; doi:10.3390/genes9050228)
Supplement: Supplementary file 1 [file genes-09-00228-s001.zip › Supplementary material/Supplementary Table 2.docx]

**Table 2.** Missense SNPs in poliovirus 2A protease predicted to be deleterious using PROVEAN, SNPs & GO Meta-SNP and Predict SNP. I-Mutant2.0, STRUM and EASE-MM web servers were used for stability analysis.

| **Mutation** | **Damaging SNPs** | | | | **Destabilizing SNPs** | | |
| --- | --- | --- | --- | --- | --- | --- | --- |
|  | **PROVEAN** | **SNPs & GO.** | **Meta-SNP** | **Predict SNP** | **I-Mutant** | **STRUM** | **EASE-MM** |
| K15A | **√** | – | **√** | **√** | **√** | **√** | **√** |
| K15C | **√** | – | **√** | **√** | **√** | **√** | – |
| K15D | **√** | – | **√** | **√** | – | **√** | **√** |
| K15E | **√** | **√** | **√** | **√** | **√** | **√** | **√** |
| K15F | **√** | – | **√** | **√** | – | **√** | **√** |
| K15G | **√** | – | **√** | **√** | – | **√** | **√** |
| K15H | **√** | – | **√** | **√** | – | **√** | **√** |
| K15l | **√** | – | **√** | **√** | **√** | **√** | **√** |
| K15N | **√** | – | **√** | **√** | **√** | **√** | **√** |
| K15P | **√** | **√** | **√** | **√** | **√** | **√** | **√** |
| K15Q | **√** | – | **√** | **√** | **√** | **√** | **√** |
| K15T | **√** | – | **√** | **√** | **√** | **√** | **√** |
| K15W | **√** | – | **√** | **√** | **√** | **√** | **√** |
| K15Y | **√** | – | **√** | **√** | **√** | **√** | **√** |
| C17D | **√** | **√** | **√** | **√** | **√** | **√** | **√** |
| C17E | **√** | **√** | **√** | **√** | **√** | **√** | **√** |
| C17G | **√** | **√** | **√** | **√** | **√** | **√** | **√** |
| C17H | **√** | – | **√** | **√** | **√** | **√** | **√** |
| C17N | **√** | – | **√** | **√** | **√** | **√** | **√** |
| C17P | **√** | **√** | – | **√** | **√** | **√** | **√** |
| C17Q | **√** | – | **√** | **√** | **√** | **√** | **√** |
| C17S | **√** | – | **√** | **√** | **√** | **√** | **√** |
| C17W | **√** | – | **√** | **√** | **√** | **√** | **√** |
| H20A | **√** | – | **√** | **√** | **√** | **√** | **√** |
| H20C | **√** | – | **√** | **√** | **√** | **√** | **√** |
| H20D | **√** | **√** | **√** | **√** | **√** | **√** | **√** |
| H20E | **√** | **√** | **√** | **√** | **√** | **√** | **√** |
| H20F | **√** | – | **√** | **√** | **√** | **√** | **√** |
| H20G | **√** | – | **√** | **√** | **√** | **√** | **√** |
| H20I | **√** | – | **√** | **√** | **√** | **√** | **√** |
| H20K | **√** | **√** | **√** | **√** | **√** | **√** | **√** |
| H20L | **√** | – | **√** | **√** | **√** | **√** | **√** |
| H20M | **√** | – | **√** | **√** | **√** | **√** | **√** |
| H20N | **√** | – | **√** | **√** | **√** | **√** | **√** |
| H20P | **√** | **√** | **√** | **√** | **√** | **√** | **√** |
| H20Q | **√** | – | **√** | **√** | **√** | **√** | **√** |
| H20R | **√** | – | **√** | **√** | **√** | **√** | **√** |
| H20S | **√** | **√** | **√** | **√** | **√** | **√** | **√** |
| H20T | **√** | – | **√** | **√** | **√** | **√** | **√** |
| H20V | **√** | – | **√** | **√** | **√** | **√** | **√** |
| H20W | **√** | – | **√** | **√** | **√** | **√** | **√** |
| H20Y | **√** | – | **√** | **√** | **√** | **√** | **√** |
| C55A | **√** | **√** | **√** | **√** | **√** | **√** | **√** |
| C55D | **√** | **√** | **√** | **√** | **√** | **√** | **√** |
| C55E | **√** | **√** | **√** | **√** | **√** | **√** | **√** |
| C55F | **√** | **√** | **√** | **√** | **√** | **√** | **√** |
| C55G | **√** | **√** | **√** | **√** | **√** | **√** | **√** |
| C55H | **√** | **√** | **√** | **√** | **√** | **√** | **√** |
| C55I | **√** | **√** | **√** | **√** | **√** | **√** | **√** |
| C55K | **√** | **√** | **√** | **√** | **√** | **√** | **√** |
| C55L | **√** | **√** | **√** | **√** | **√** | **√** | **√** |
| C55M | **√** | **√** | **√** | **√** | **√** | **√** | **√** |
| C55N | **√** | **√** | **√** | **√** | **√** | **√** | **√** |
| C55P | **√** | **√** | **√** | **√** | **√** | **√** | **√** |
| C55Q | **√** | **√** | **√** | **√** | **√** | **√** | **√** |
| C55R | **√** | **√** | **√** | **√** | **√** | **√** | **√** |
| C55S | **√** | **√** | **√** | **√** | **√** | **√** | **√** |
| C55T | **√** | **√** | **√** | **√** | **√** | **√** | **√** |
| C55V | **√** | **√** | **√** | **√** | **√** | **√** | **√** |
| C55W | **√** | **√** | **√** | **√** | **√** | **√** | **√** |
| C55Y | **√** | **√** | **√** | **√** | **√** | **√** | **√** |
| C57A | **√** | **√** | **√** | **√** | **√** | **√** | **√** |
| C57D | **√** | **√** | **√** | **√** | **√** | **√** | **√** |
| C57E | **√** | **√** | **√** | **√** | **√** | **√** | **√** |
| C57F | **√** | **√** | **√** | **√** | **√** | **√** | **√** |
| C57G | **√** | **√** | **√** | **√** | **√** | **√** | **√** |
| C57H | **√** | **√** | **√** | **√** | **√** | **√** | **√** |
| C57I | **√** | **√** | **√** | **√** | **√** | **√** | **√** |
| C57K | **√** | **√** | **√** | **√** | **√** | **√** | **√** |
| C57L | **√** | **√** | **√** | **√** | **√** | **√** | **√** |
| C57M | **√** | **√** | **√** | **√** | **√** | **√** | **√** |
| C57N | **√** | **√** | **√** | **√** | **√** | **√** | **√** |
| C57P | **√** | **√** | **√** | **√** | **√** | **√** | **√** |
| C57Q | **√** | **√** | **√** | **√** | **√** | **√** | **√** |
| C57R | **√** | **√** | **√** | **√** | **√** | **√** | **√** |
| C57S | **√** | **√** | **√** | **√** | **√** | **√** | **√** |
| C57T | **√** | **√** | **√** | **√** | **√** | **√** | **√** |
| C57V | **√** | **√** | **√** | **√** | **√** | **√** | **√** |
| C57W | **√** | **√** | **√** | **√** | **√** | **√** | **√** |
| C57Y | **√** | **√** | **√** | **√** | **√** | **√** | **√** |
| C64Y | **√** | **√** | **√** | **√** | **√** | **√** | **√** |
| D108A | **√** | **√** | **√** | **√** | **√** | **√** | **√** |
| D108C | **√** | – | **√** | **√** | **√** | **√** | **√** |
| D108E | **√** | **√** | **√** | **√** | **√** | **√** | **√** |
| D108F | **√** | **√** | **√** | **√** | **√** | **√** | **√** |
| D108G | **√** | – | **√** | **√** | **√** | **√** | **√** |
| D108H | **√** | – | **√** | **√** | **√** | **√** | **√** |
| D108I | **√** | – | **√** | **√** | **√** | **√** | **√** |
| D108K | **√** | **√** | **√** | **√** | **√** | **√** | **√** |
| D108L | **√** | **√** | **√** | **√** | **√** | **√** | **√** |
| D108M | **√** | **√** | **√** | **√** | **√** | **√** | **√** |
| D108N | **√** | **√** | **√** | **√** | **√** | **√** | **√** |
| D108P | **√** | **√** | **√** | **√** | **√** | **√** | **√** |
| D108Q | **√** | **√** | **√** | **√** | **√** | **√** | **√** |
| D108R | **√** | – | **√** | **√** | **√** | **√** | **√** |
| D108S | **√** | **√** | **√** | **√** | **√** | **√** | **√** |
| D108T | **√** | – | **√** | **√** | **√** | **√** | **√** |
| D108V | **√** | **√** | **√** | **√** | **√** | **√** | **√** |
| D108W | **√** | **√** | **√** | **√** | **√** | **√** | **√** |
| D108Y | **√** | **√** | **√** | **√** | **√** | **√** | **√** |
| C109A | **√** | **√** | **√** | **√** | **√** | **√** | **√** |
| C109D | **√** | **√** | **√** | **√** | **√** | **√** | **√** |
| C109E | **√** | **√** | **√** | **√** | **√** | **√** | **√** |
| C109F | **√** | **√** | **√** | **√** | **√** | **√** | **√** |
| C109G | **√** | **√** | **√** | **√** | **√** | **√** | **√** |
| C109H | **√** | **√** | **√** | **√** | **√** | **√** | **√** |
| C109I | **√** | **√** | **√** | **√** | **√** | **√** | **√** |
| C109K | **√** | **√** | **√** | **√** | **√** | **√** | **√** |
| C109L | **√** | **√** | **√** | **√** | **√** | **√** | **√** |
| C109M | **√** | **√** | **√** | **√** | **√** | **√** | **√** |
| C109N | **√** | **√** | **√** | **√** | **√** | **√** | **√** |
| C109P | **√** | **√** | **√** | **√** | **√** | **√** | **√** |
| C109Q | **√** | **√** | **√** | **√** | **√** | **√** | **√** |
| C109R | **√** | **√** | **√** | **√** | **√** | **√** | **√** |
| C109S | **√** | **√** | **√** | **√** | **√** | **√** | **√** |
| C109T | **√** | **√** | **√** | **√** | **√** | **√** | **√** |
| C109V | **√** | **√** | **√** | **√** | **√** | **√** | **√** |
| C109W | **√** | **√** | **√** | **√** | **√** | **√** | **√** |
| C109Y | **√** | **√** | **√** | **√** | **√** | **√** | **√** |
| G110A | **√** | **√** | **√** | **√** | **√** | **√** | **√** |
| G110C | **√** | **√** | **√** | **√** | **√** | **√** | **√** |
| G110D | **√** | **√** | **√** | **√** | **√** | **√** | **√** |
| G110E | **√** | **√** | **√** | **√** | **√** | **√** | **√** |
| G110F | **√** | **√** | **√** | **√** | **√** | **√** | **√** |
| G110H | **√** | **√** | **√** | **√** | **√** | **√** | **√** |
| G110I | **√** | **√** | **√** | **√** | **√** | **√** | **√** |
| G110K | **√** | **√** | **√** | **√** | **√** | **√** | **√** |
| G110L | **√** | **√** | **√** | **√** | **√** | **√** | **√** |
| G110M | **√** | – | **√** | **√** | **√** | **√** | **√** |
| G110N | **√** | **√** | **√** | **√** | **√** | **√** | **√** |
| G110P | **√** | **√** | **√** | **√** | **√** | **√** | **√** |
| G110Q | **√** | **√** | **√** | **√** | **√** | **√** | **√** |
| G110R | **√** | **√** | **√** | **√** | **√** | **√** | **√** |
| G110S | **√** | **√** | **√** | **√** | **√** | **√** | **√** |
| G110T | **√** | **√** | **√** | **√** | **√** | **√** | **√** |
| G110V | **√** | **√** | **√** | **√** | **√** | **√** | **√** |
| G110W | **√** | **√** | **√** | **√** | **√** | **√** | **√** |
| G110Y | **√** | **√** | **√** | **√** | **√** | **√** | **√** |

SNPs were predicted as destabilizing in nature if two or more than two algorithms showed a decrease in stability upon mutation. √, Yes; –, No
