# Supplementary figures and images for: Structure-Function Mutational Analysis and Prediction of the Potential Impact of High Risk Non-Synonymous Single-Nucleotide Polymorphism on Poliovirus 2A Protease Stability Using Comprehensive Informatics Approaches
Source: Genes (Basel). 2018 Apr 26;9(5):228. doi: 10.3390/genes9050228 (PMC5977168; doi:10.3390/genes9050228)

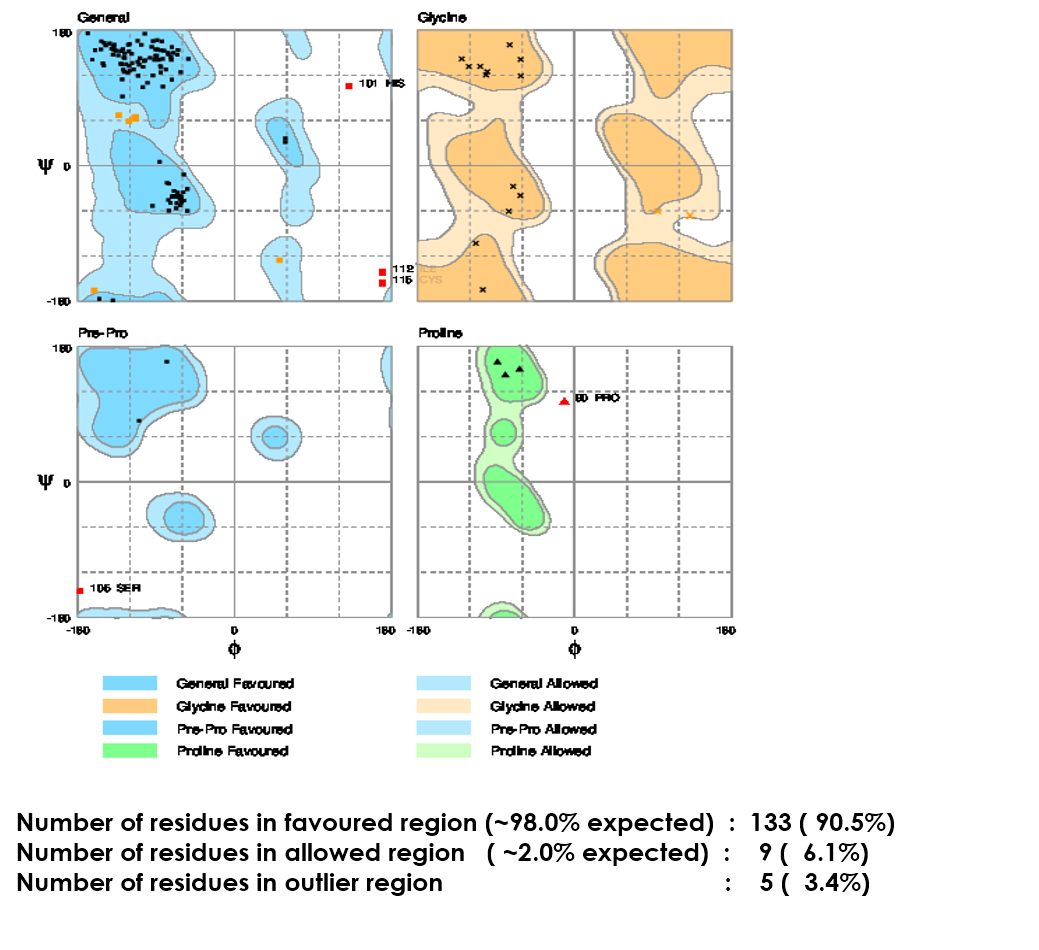

Supplement: Supplementary file 1 [file genes-09-00228-s001.zip › Supplementary material/Supplementary Fig. S1.tif]

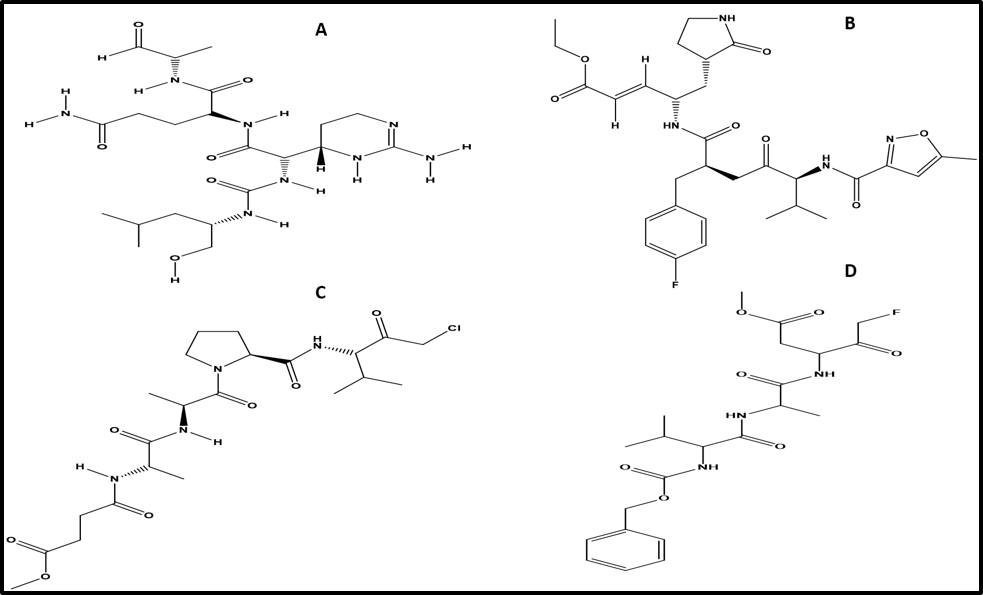

Supplement: Supplementary file 1 [file genes-09-00228-s001.zip › Supplementary material/Supplementary Fig. S3.tif]
